# Irreproducible results and unsupported conclusions in Ahmad et al. [BMC genomics (2020) 21:656]

**DOI:** 10.1186/s12864-023-09883-4

**Published:** 2023-12-18

**Authors:** Francisco J. Ruiz-Ruano, Juan Pedro M. Camacho

**Affiliations:** 1 School of Biological Sciences, University of East Anglia, Norwich Research Park, Norwich, NR4 7TU UK; 2 Department of Organismal Biology – Systematic Biology, Evolutionary Biology Centre, Uppsala University, Uppsala, SE-752 36 Sweden; 3 Centre for Molecular Biodiversity Research, Leibniz Institute for the Analysis of Biodiversity Change, Adenauerallee 127, 53113 Bonn, Germany; 4 Departamento de Genética, Universidad de Granada, Granada, 18071 Spain

**Keywords:** B chromosomes, Genes, Genomic DNA, Normalization, Transcriptome

## Results

### Misleading datasets

We tried to reproduce the results following paper’s methods and found a series of inconsistencies in the information contained in the Figures, Tables and Supplementary Datasets which made difficult this task. These were:

1. Some accession numbers in the coverage plots of Figs. S7 and S8 are not found in their “Supplementary_dataset_3” file (from here onwards abbreviated as “Sup_dataset_3”) (see our supplementary text note 1).
2. In trying to download the list of contigs annotated as genes in Sup_dataset_3, *A. correntinus* sheet, we realized that the repository links are wrong (see our supplementary text note 2).
3. The reads deposited in the Sequence Read Archive (SRA) had already been trimmed but it was not specified in the paper (see our supplementary text note 3).
4. The information about *A. mexicanus* and *A. correntinus* in Table 3 and Sup_dataset_3 was mistakenly interchanged (see our supplementary text note 4).

### Problems to reproduce the *A. correntinus* results

In addition, we found a crucial mistake in the calculation of 1B/0B coverage ratios in Sup_dataset_3, as size difference in the 0B and 1B libraries were not normalized. The 1B library (505,608,854 reads) was 30.37% larger than the 0B one (387,817,038 reads), so that, if all the reads were used in the mappings (and it was not specified to have used the same number of reads from both libraries), the 1B sequences had a starting advantage to reach the 1.5 minimum coverage ratio expected (which is based on assuming that the number of copies was equal for A and B chromosomes, so that a library with 1B would carry 50% more copies than a 0B one). Therefore, library size difference, alone, already implied 1.3 departure coverage ratios (in favour of the 1B library) and this could determine that many contigs reached the 1.5 coverage ratio, by chance, thus being false positives.

To test this possibility, we performed mappings of genomic DNA (gDNA) on two sets of reference transcripts, since the source for the gene annotation is unclear. We extracted 61 contigs from the database of the European Bioinformatics Institute (EBI) and 26 contigs downloaded from the database of the National Center for Biotechnology Information (NCBI) (see our Supplementary text note 2), with two different approaches. We first performed the mappings using the same software used in [1] (Bowtie2), as indicated in our Supplementary text note 5 (mapping results are shown in our Supplementary_file_1). For comparison, we also performed mappings with the SSAHA2 software, with the options we usually apply to map genomic reads on a transcriptome reference (see our Supplementary text note 6 and Supplementary_file_1). The results of our mappings, expressed as “reads mapped”, and without normalizing for library size differences (as it was apparently done in [1]), revealed that out of the 61 EBI contigs annotated as protein-coding genes in [1], all of which showed 1B/0B > 1.5 in its Sup_dataset_3, only 41 actually passed the 1.5 threshold using Bowtie2 and 48 passed it with SSAHA2 mappings (Table 1; see the “61contigs_notrim” sheet in our Supplementary_file_1). Therefore, even trying to follow paper’s indications (with the difficulties mentioned above), only 67% of the 61 EBI contigs supposedly residing on the B chromosome (according to [1]) actually passed the 1.5 threshold using Bowtie2 (79% using SSAHA2). Most importantly, when we normalized for library size, this figure decreased to 44% (see Table 1 and the “61contigs_notrim_norm” sheet in our Supplementary_file_1), indicating that almost half of the 61 contigs annotated as protein-coding genes in [1] were false positives due to the unequal size of the 0B and 1B libraries.

**Table 1:** Number of contigs passing the 1.5 coverage ratio threshold after the genomic mappings performed trying to reproduce the results in [1]. We first used the *A. mexicanus* transcriptome as reference to estimate 1B/0B coverage ratio for the contigs claimed by authors as being in the B chromosome of *A. correntinus* (61 and 26 contigs, the sequence of which we downloaded from EBI and NCBI, respectively). In different mappings, we expressed the results as “number of reads mapped” (as done in [1]) or in “number of copies per haploid genome” (as done in [2]). According to [1], all 61 and 26 contigs passed the 1.5 coverage ratio threshold. However, note the low figures of reproducibility in all our attempts, from using [1] reads as such, normalizing the results for 0B and 1B library size differences, or using equal number of reads from each library. In addition, our analysis using the transcriptome from two species (*A. scabripinnis* and *A. paranae*) being closely related to *A. correntinus*, yielded 630 contigs passing the 1.5 threshold but only one (corresponding to the *ipo11* gene) was also included in the 61 and 26 contigs considered B-genes in [1]

				No. contigs passing the 1.5 1B/0B threshold				
	Transcriptome			Ahmad et al. [1]	This study		Reproducibility	
Reference species	contigs	Expressed as	Library normalization	Bowtie2	Bowtie2	SSAHA2	Bowtie2	SSAHA2
*A. mexicanus*	61 EBI	No. reads mapped	No	61	41	48	67%	79%
			Yes		27	26	44%	43%
			No (equal no. 0B and 1B reads)		31	26	51%	43%
		No. copies	Yes		29	27	48%	44%
	26 NCBI	No. reads mapped	No	26	5	3	19%	12%
			Yes		2	3	8%	12%
			No (equal no. 0B and 1B reads)		2	3	8%	12%
		No. copies	Yes		2	3	8%	12%
*A. scabripinnis-A. paranae*		No. copies	Yes			630		*ipo11* only

In the case of the 26 NCBI contigs, normalization for unequal library sizes was even most important, as reproducibility was always lower than 20% (see Table 1 and the “26contigs” sheets in our Supplementary_file_1).

**Fig. 1:**
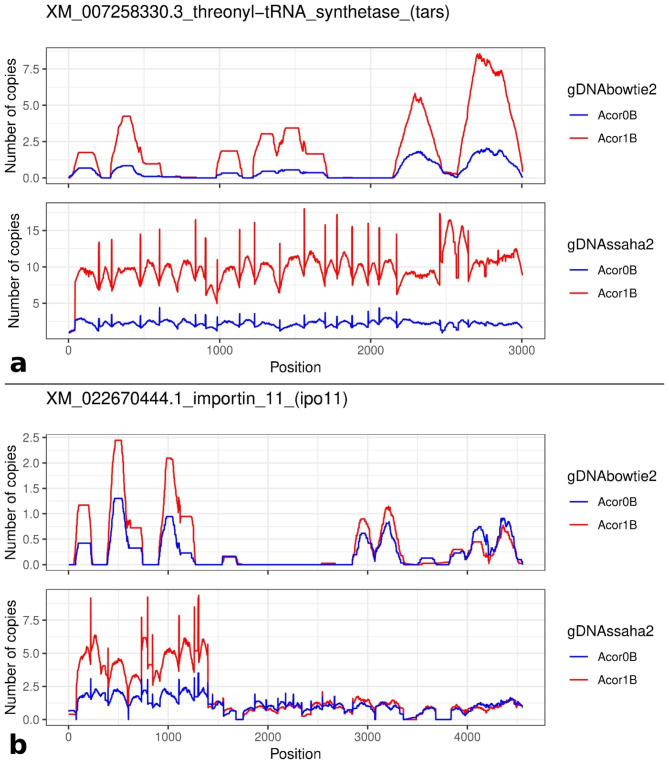
Coverage across two genes in 0B and 1B gDNA libraries of *A. correntinus*. We include here the results using Bowtie2 and SSAHA2 mappings for the *tars* (**a**) and *ipo11* (**b**) genes using the NCBI contigs as a reference. Note that, in general, coverage is 0 with Bowtie2 for the shortest exons due to not considering partially mapped reads as SSAHA2 does. By comparing the y-axis scales of both graphs within each gene, note how SSAHA2 detected about twice number of copies as Bowtie2. See fold change (1B/0B) values in our Supplementary_File_1, and coverage graphics for all genes in Supplementary_Files 2 and 3, which include the collections of 26 NCBI and the 61 EBI contigs, respectively

We repeated the mappings for the 1B library, using the same number of randomly chosen reads present in the 0B library (387,817,038), as an alternative to normalization for library size, and found about the same numbers of selected genes (see our Table 1 and Supplementary_file_2), indicating that either of the two methods to compensating library size differences would have worked. Likewise, after expressing coverage ratio as number of copies per haploid genome, thus implying normalization for library and genome size, the results were highly similar to those mentioned above for read mapping calculations with normalization (or using the same number of reads from both libraries) (see our Table 1 and Supplementary_files 3–5), with 48% (Bowtie2) and 44% (SSAHA2) reproducibility in the case of the 61 EBI contigs, and only 8% and 12% reproducibility, respectively, for the 26 NCBI contigs. In fact, only two genes (*tars* and *ipo11*) surpassed the 1.5 threshold in both contig lists (Fig. 1).

As a whole, our results demonstrate that more than half of the contigs claimed in [1] as showing overabundance in the 1B library (compared with the 0B one) were false positives due to not considering the fact that the 1B library contained 30% more reads than the 0B one. In addition, only three out of the 26 contigs that we identified by their annotation name in Sup_dataset_3, surpassed the 1.5 threshold coverage ratio, thus throwing serious doubts on the reliability of this paper results.

## Discussion

We demonstrate here that the results in [1] are impossible to reproduce due to multiple inconsistencies within the maintext, Figures, Tables and supplementary information. In addition, we have detected a methodological error that makes invalid the immense majority of their results, as it affects the calculation of coverage ratios in the three species analyzed. It was the absence of normalization to correct for size differences of the B-lacking and B-carrying libraries, since it introduced many false positives in the lists of B chromosome genes. This lack of normalization leads to a series of unsupported claimings in the paper, listed below, also including some important conceptual errors (see an extended version of this section in our Supplementary text note 7):

1. The first sentence in the Background section (page 2) includes a wrong definition on B chromosomes by saying that they lack the ability of meiotic pairing unlike the normal A chromosomes.
2. Some references are not appropriately used.
3. The null hypothesis enunciated on page 2 is invalid because selfish transmission has not been shown in any of the three species analyzed in this paper.
4. Claiming that “*considerable amount of genomic portions have been migrated from A chromosomes to B via transpositions, duplications and rearrangements events”* is not supported by authors’ data.
5. The following sentence, on page 3: *“It seems that B chromosomes tend to gain sequences that are crucial for their own establishment inside the cell”* is an anti-Darwinian post-adaptive statement.
6. The paper includes inappropriate data from microdissected B chromosomes to reach strong but unsupported conclusions on B chromosome gene content. Also, due to extremely low coverage and clear bias towards repetitive sequences of this material, we found inappropiate to infer GO functions without specifying the actual number of genes which they are based on.
7. The suggestion (in the beginning of page 17) that *“Bs might have played some role in shaping the genome evolution for effective adaptation in cave environment”* in the case of *A. mexicanus* is not supported by this paper results, and more when the list of B-genes for this species reported in [1] coincides only in one gene (*ncaph2*) with the list of B-genes recently reported by Imarazene et al. [3]. Even though both analyses would have dealt with different B chromosomes, and bearing also in mind our comments on point no. 6, we consider that the claim (in [1]) that *“B chromosomes plays a role in adaptation acting on metabolisms”* is untenable.
8. Finally, we find it highly inappropriate to convert the title of our 2019 bioRxiv preprint [4] (“Evolutionary success of a parasitic B chromosome rests on gene content**”)** literally into an “emerging hypothesis” (“evolutionary success of the B chromosome lies on its gene contents”) without mentioning the source (see page 16, column on the right in [1]).

Good reproducibility practices are recommended for validating bioinformatics analyses, including the use of workflow packages and managers [5]. In addition, it is the task of journals to improve the review system for this type of work.

### Electronic Supplementary Material

Below is the link to the electronic supplementary material.

Supplementary File 1: Comparison of the number of mapped reads reported in [1] and our own mappings, and the corresponding coverage ratio values (in red when >1.5) using Bowtie2 and SSAHA2, against the 61 and 26 transcriptome contigs downloaded from EBI and NCBI, respectively. Two sheets are shown for each contig collection, one without normalization for 0B and 1B library size differences between 0B and 1B, as donde in [1], and another performing such normalization. Note how the number of contigs passing the 1.5 1B/0B ratio (last row in each sheet) was much lower when normalization was performed.

Supplementary File 2: Comparison between the normalization for library size performed in Supplementary File 1 and using the same number of reads from the 0B and 1B libraries. Note the low difference in the figures obtained by the two methods in the Q and S columns, indicating that either normalization or using the same number of reads from each library could have yielded roughly similar results. Also note the exact coincidence in the result with SSAHA2 mapping (26 contigs passing the 1.5 threshold) and the slight difference with Bowtie2 (27 and 31) in the case of the 61 contigs collection, but exact coincidence for both types of mapping (2 and 3) for the 26 contigs collection.

Supplementary File 3: Gene abundances normalized by library and genome size, thus expressed in copy number per haploid genome. Two sheets are shown for the 61 EBI and the 26 NCBI contig collections, and a third sheet showing how only two genes (*tars* and *ipo11*) surpassed the 1.5 threshold in both contig collections.

Supplementary File 4: Coverage across the 26 NCBI contigs in 0B and 1B gDNA libraries of *A. correntinus* comparing the Bowtie2 and the SSAHA2 mappings. Average values and 1B/0B ratios are shown in Supplementary File 3.

Supplementary File 5: Coverage across the 61 EBI contigs in 0B and 1B gDNA libraries of *A. correntinus* comparing the Bowtie2 and the SSAHA2 mappings. Average values and 1B/0B ratios are shown in Supplementary File 3.

Supplementary File 6: Sequences of the 61 contigs from the *A. mexicanus* transcriptome retrieved from EBI used in this study.

Supplementary File 7: BLASTX best matches of the 61 EBI contigs against the NR database. Note that only 16 matches were coincident with those in [1] (shaded in green colour), 18 showed incongruent matches (shaded in brown colour) and 27 showed no matches (unshaded).

Supplementary File 8: Sequences of the 26 contigs from the *A. mexicanus* transcriptome retrieved from NCBI used in this study.

Supplementary File 9: Complementary text to better understand our methods, results and discussion.

## Data Availability

Data analyzed here were downloaded from supplementary information in [1] and several public databases.
