# Supplementary material for: Irreproducible results and unsupported conclusions in Ahmad et al. [BMC genomics (2020) 21:656]
Source: BMC Genomics. 2023 Dec 18;24:778. doi: 10.1186/s12864-023-09883-4 (PMC10726643; doi:10.1186/s12864-023-09883-4)
Supplement: Supplementary file 4 — Coverage across the 26 NCBI contigs in 0B and 1B gDNA libraries of A. correntinus comparing the Bowtie2 and the SSAHA2 mappings. Average values and 1B/0B ratios are shown in Supplementary File 3. [file 12864_2023_9883_MOESM4_ESM.pdf]

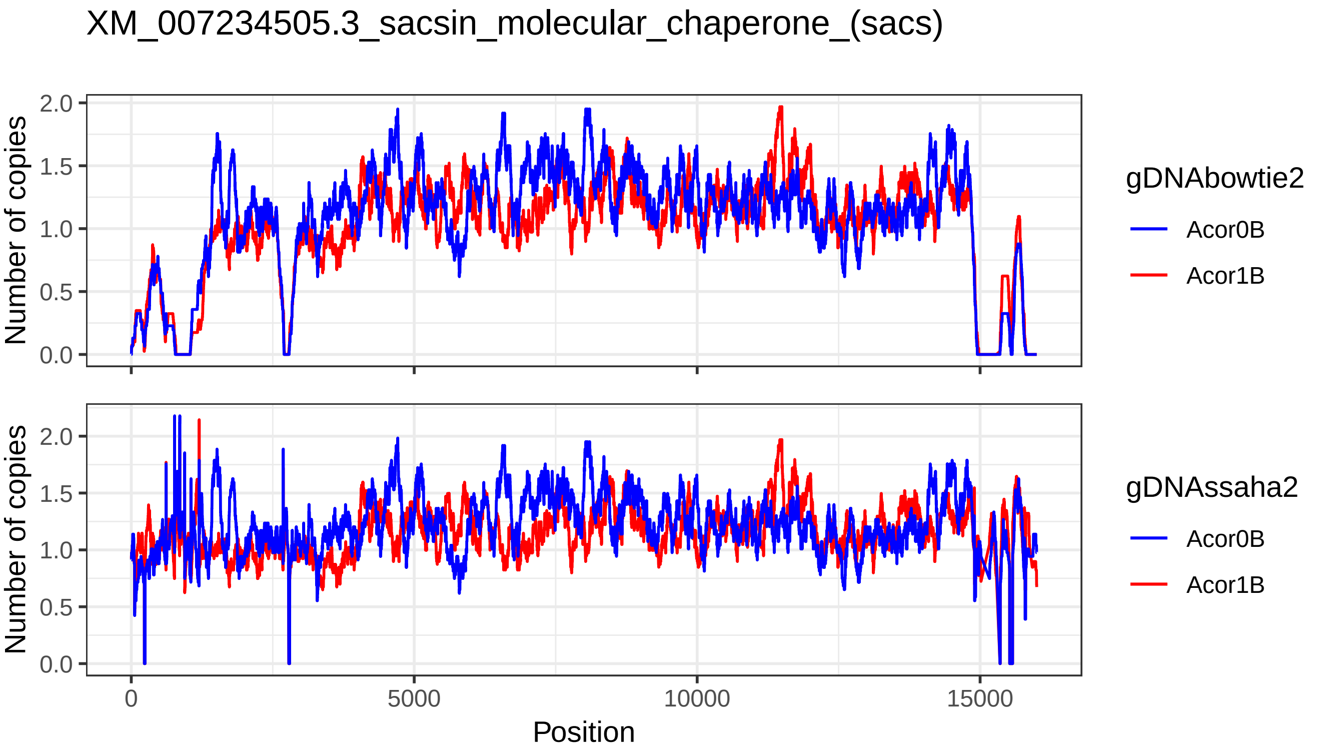

XM_007234505.3_sacsin_molecular_chaperone_(sacs)
Number of copies
2.0
1.5
1.0
0.5
0.0
gDNAbowtie2
Acor0B
Acor1B
gDNAssaha2
Acor0B
Acor1B
0
5000
10000
15000
Position

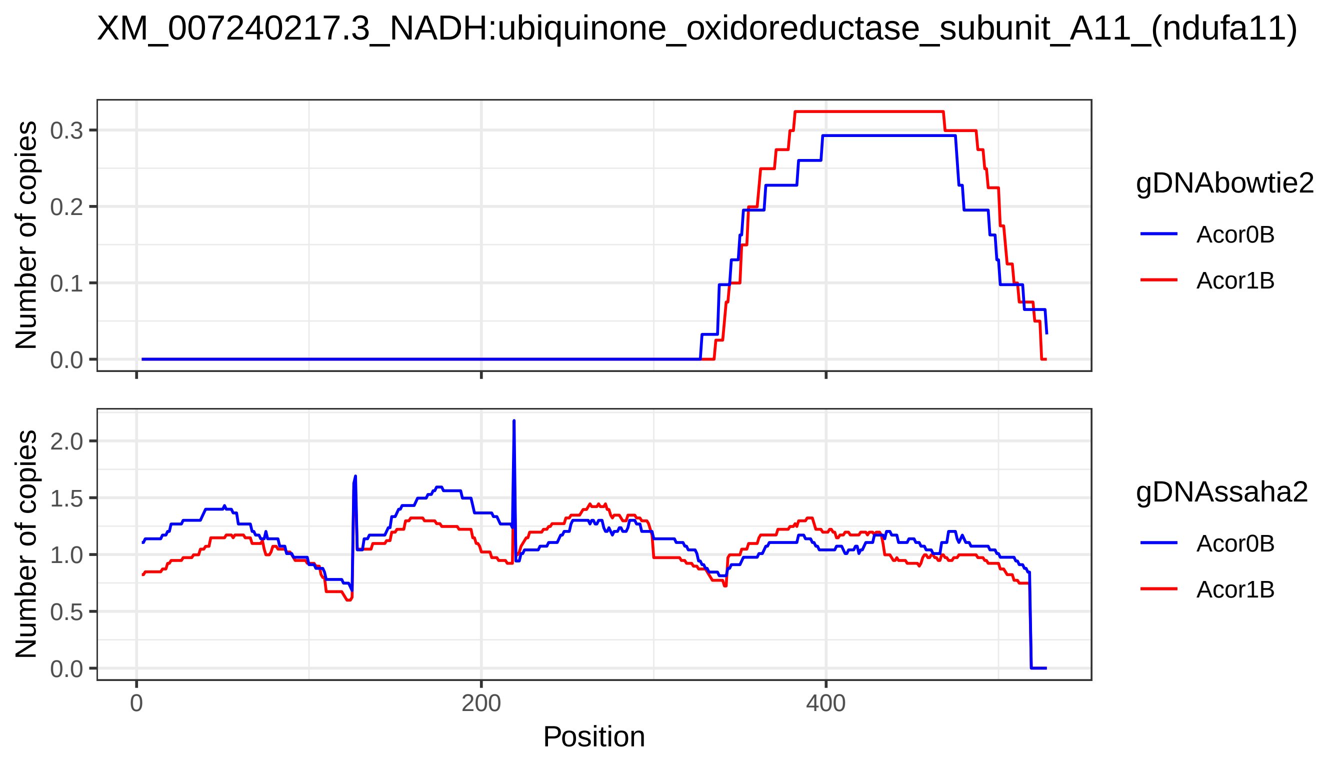
XM_007240217.3_NADH:ubiquinone_oxidoreductase_subunit_A11_(ndufa11)
Number of copies
0.3
0.2
0.1
0.0
gDNAbowtie2
Acor0B
Acor1B
Number of copies
2.0
1.5
1.0
0.5
0.0
gDNAssaha2
Acor0B
Acor1B
0
200
400
Position

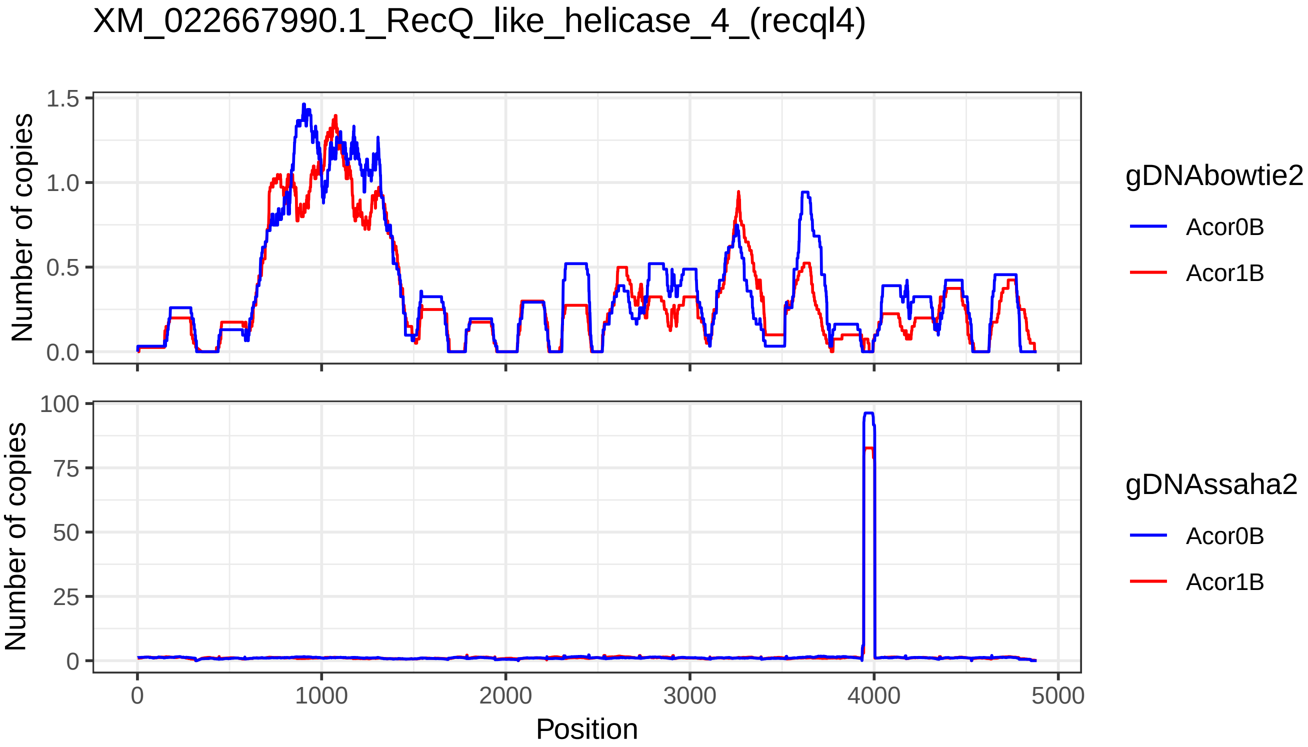

XM_022667990.1_RecQ_like_helicase_4_(recql4)
Number of copies
1.5
1.0
0.5
0.0
gDNAbowtie2
Acor0B
Acor1B
Number of copies
100
75
50
25
0
gDNAssaha2
Acor0B
Acor1B
0
1000
2000
3000
4000
5000
Position

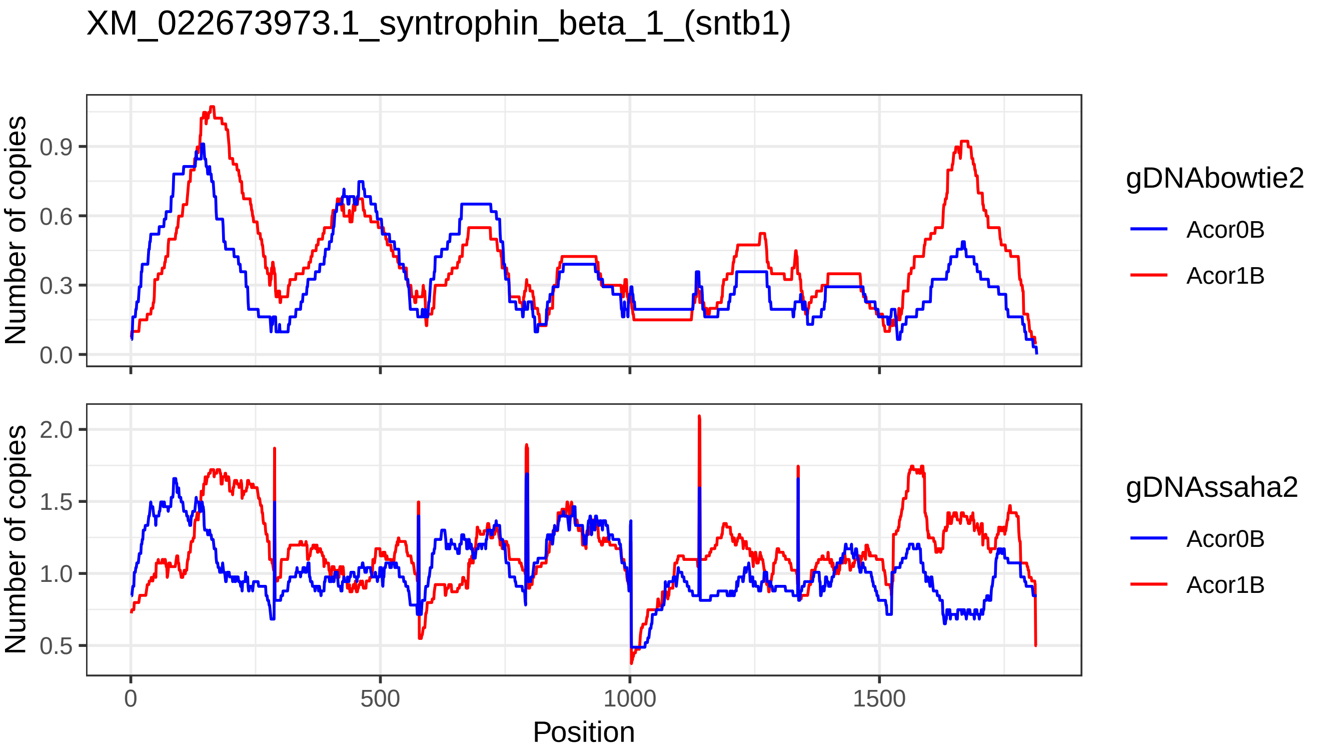

XM_022673973.1_syntrophin_beta_1_(sntb1)
Number of copies
0.0
0.3
0.6
0.9
gDNAbowtie2
Acor0B
Acor1B
Number of copies
0.5
1.0
1.5
2.0
gDNAssaha2
Acor0B
Acor1B
0
500
1000
1500
Position

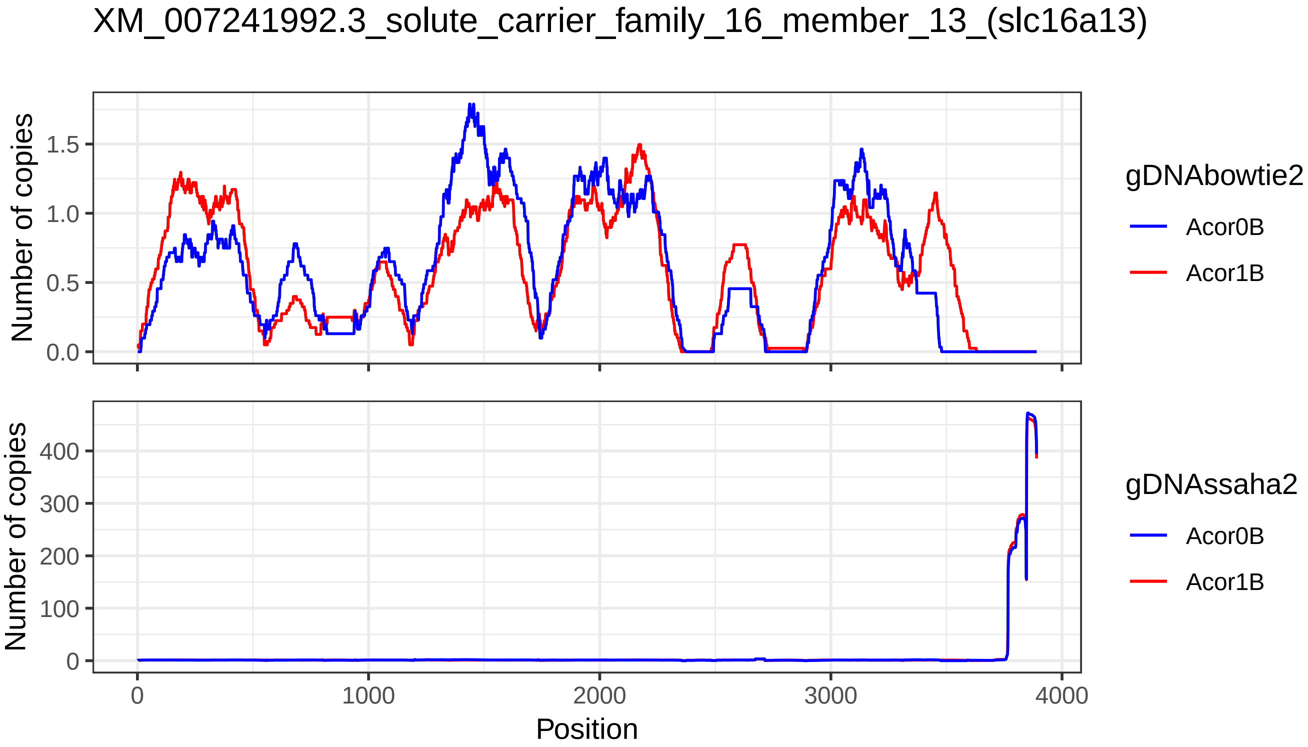
XM_007241992.3_solute_carrier_family_16_member_13_(slc16a13)
Number of copies
1.5
1.0
0.5
0.0
gDNAbowtie2
Acor0B
Acor1B
Number of copies
400
300
200
100
0
gDNAssaha2
Acor0B
Acor1B
0
1000
2000
3000
4000
Position

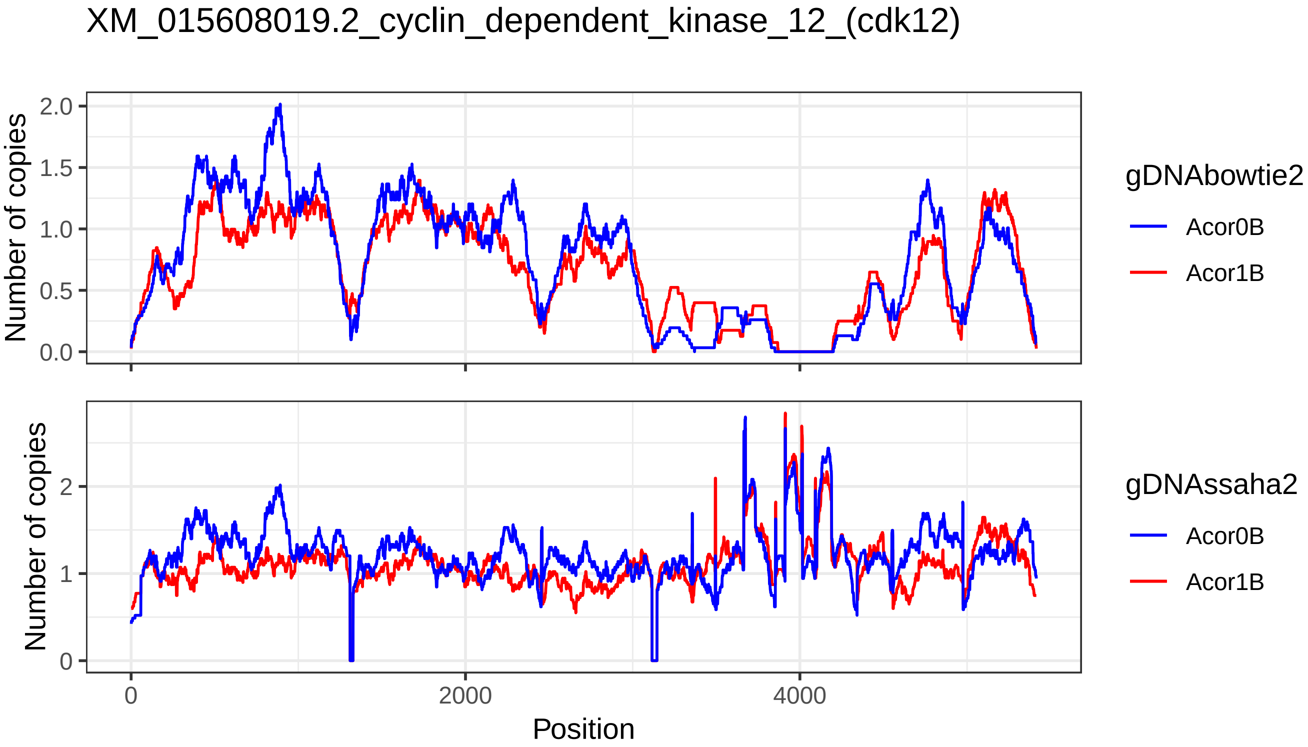
XM_015608019.2_cyclin_dependent_kinase_12_(cdk12)
Number of copies
2.0
1.5
1.0
0.5
0.0
gDNAbowtie2
Acor0B
Acor1B
Number of copies
2
1
0
gDNAssaha2
Acor0B
Acor1B
0
2000
4000
Position

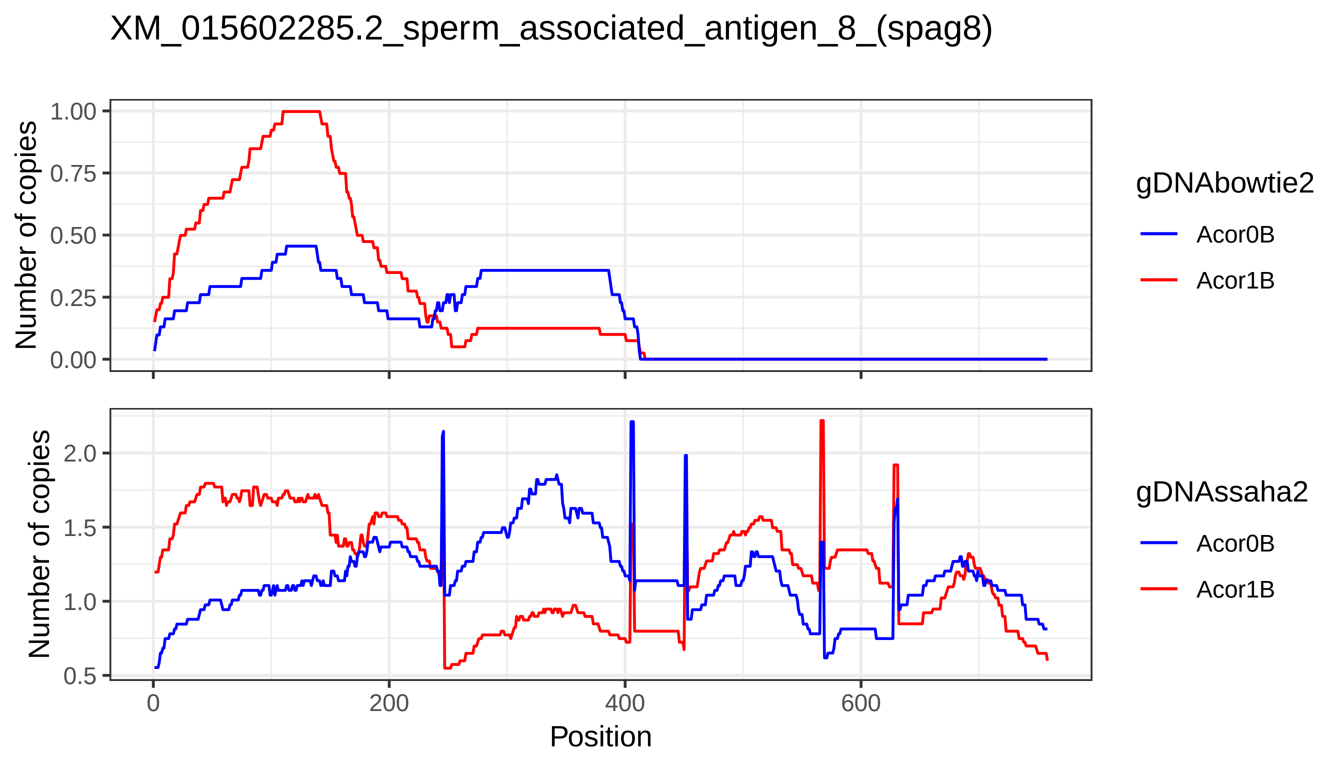

XM_015602285.2_sperm_associated_antigen_8_(spag8)
Number of copies
1.00
0.75
0.50
0.25
0.00
gDNAbowtie2
Acor0B
Acor1B
Number of copies
2.0
1.5
1.0
0.5
gDNAssaha2
Acor0B
Acor1B
0
200
400
600
Position

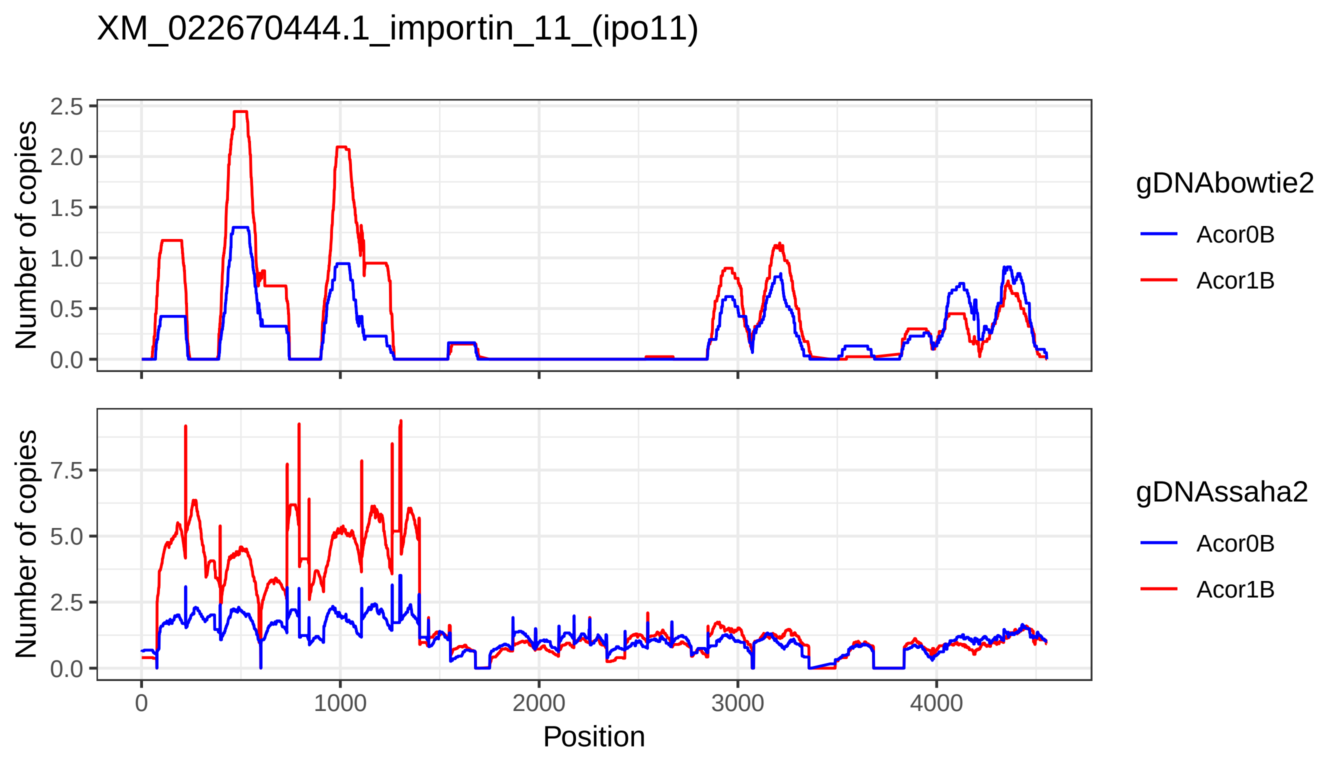

XM_022670444.1_importin_11_(ipo11)
Number of copies
2.5
2.0
1.5
1.0
0.5
0.0
gDNAbowtie2
Acor0B
Acor1B
Number of copies
7.5
5.0
2.5
0.0
gDNAssaha2
Acor0B
Acor1B
0
1000
2000
3000
4000
Position

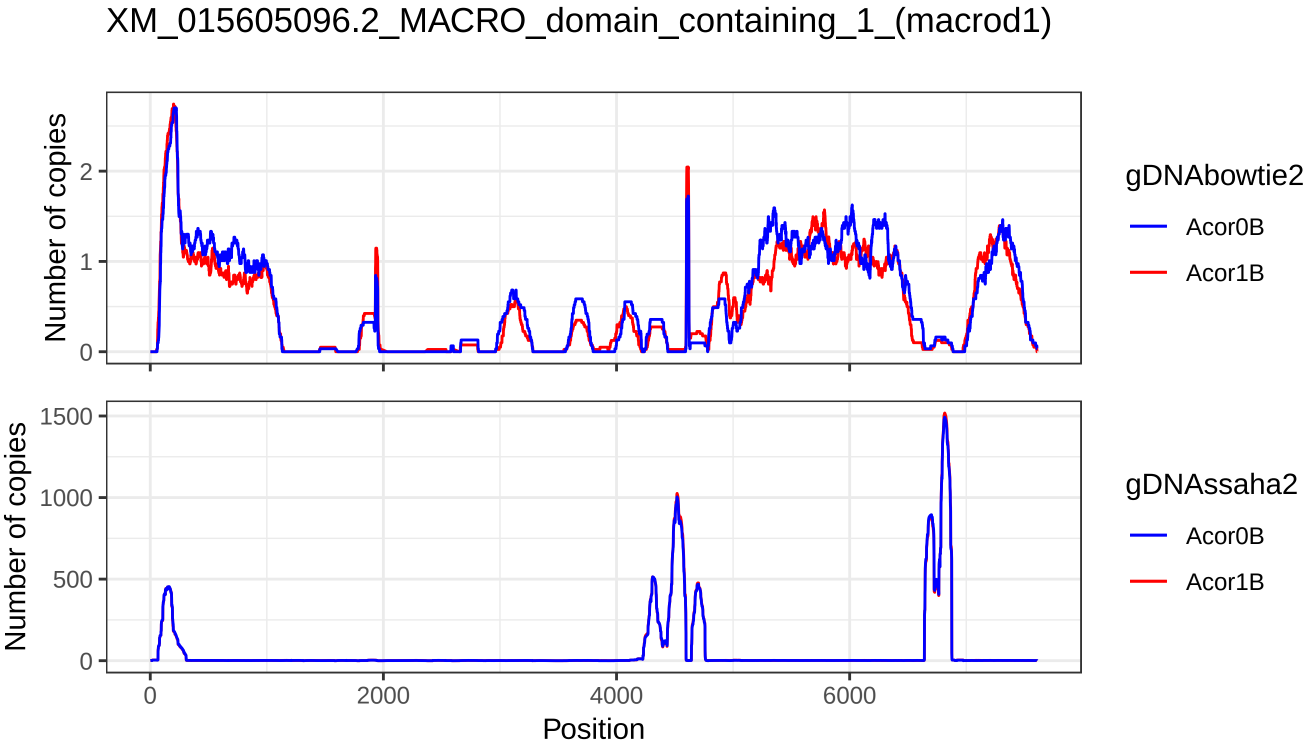
XM_015605096.2_MACRO_domain_containing_1_(macrod1)
Number of copies
0
1
2
gDNAbowtie2
Acor0B
Acor1B
Number of copies
0
500
1000
1500
gDNAssaha2
Acor0B
Acor1B
0
2000
4000
6000
Position

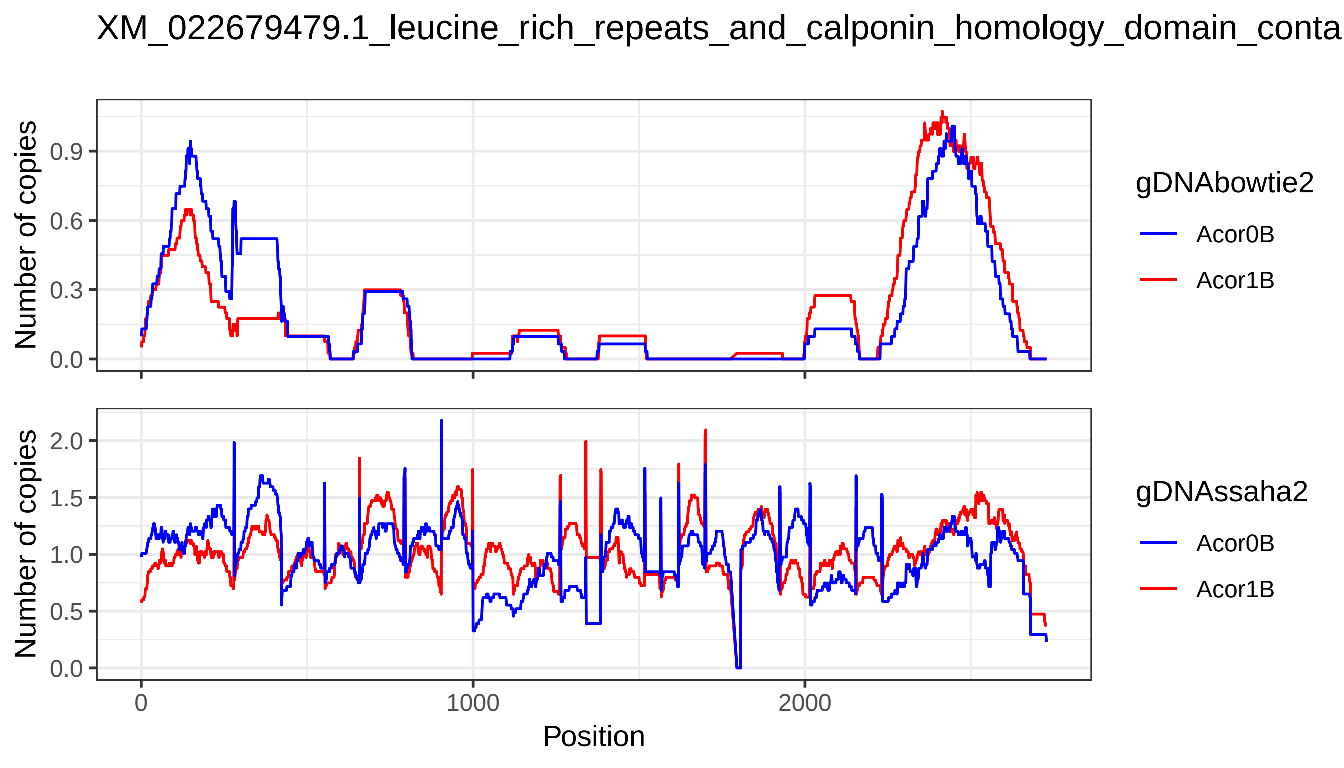

XM_022679479.1_leucine_rich_repeats_and_calponin_homology_domain_conta
Number of copies
0.0
0.3
0.6
0.9
gDNAbowtie2
Acor0B
Acor1B
Number of copies
0.0
0.5
1.0
1.5
2.0
gDNAssaha2
Acor0B
Acor1B
0
1000
2000
Position

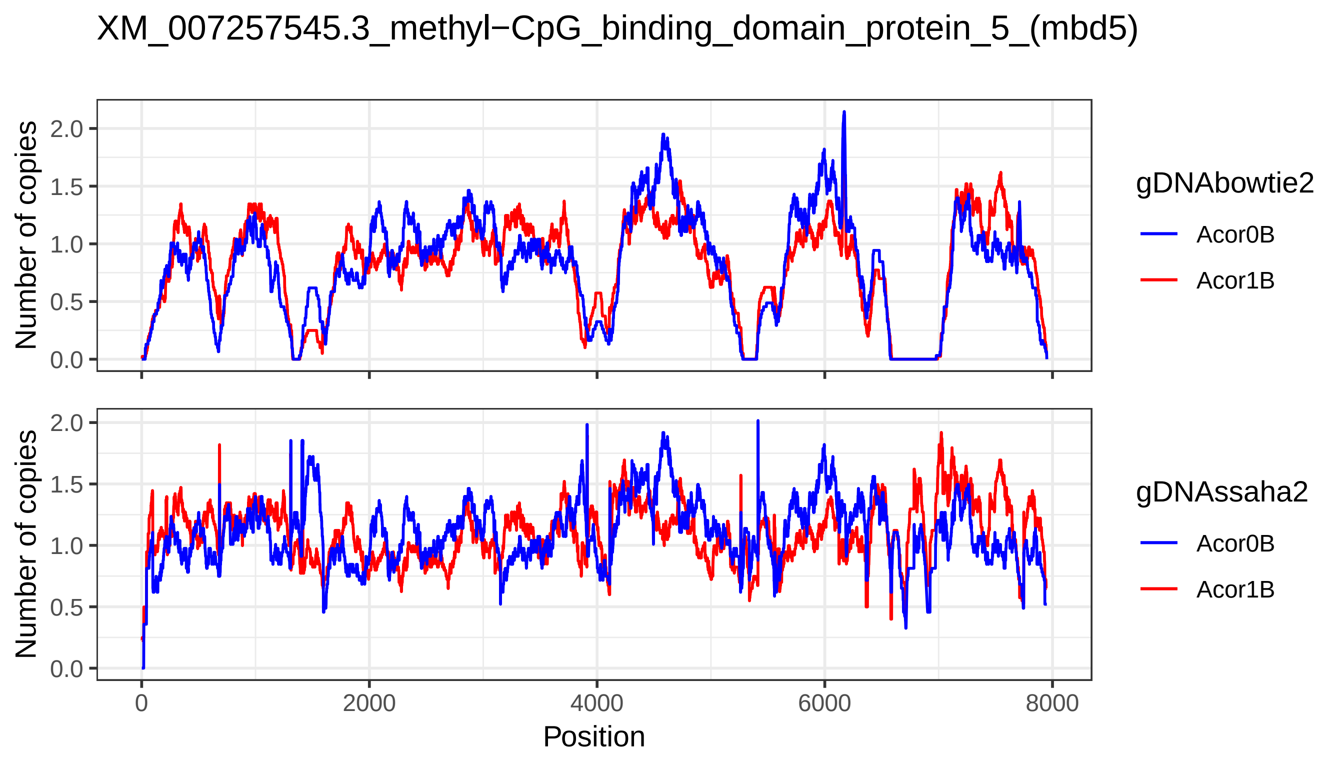

XM_007257545.3_methyl−CpG_binding_domain_protein_5_(mbd5)
Number of copies
2.0
1.5
1.0
0.5
0.0
gDNAbowtie2
Acor0B
Acor1B
gDNAssaha2
Acor0B
Acor1B
0
2000
4000
6000
8000
Position

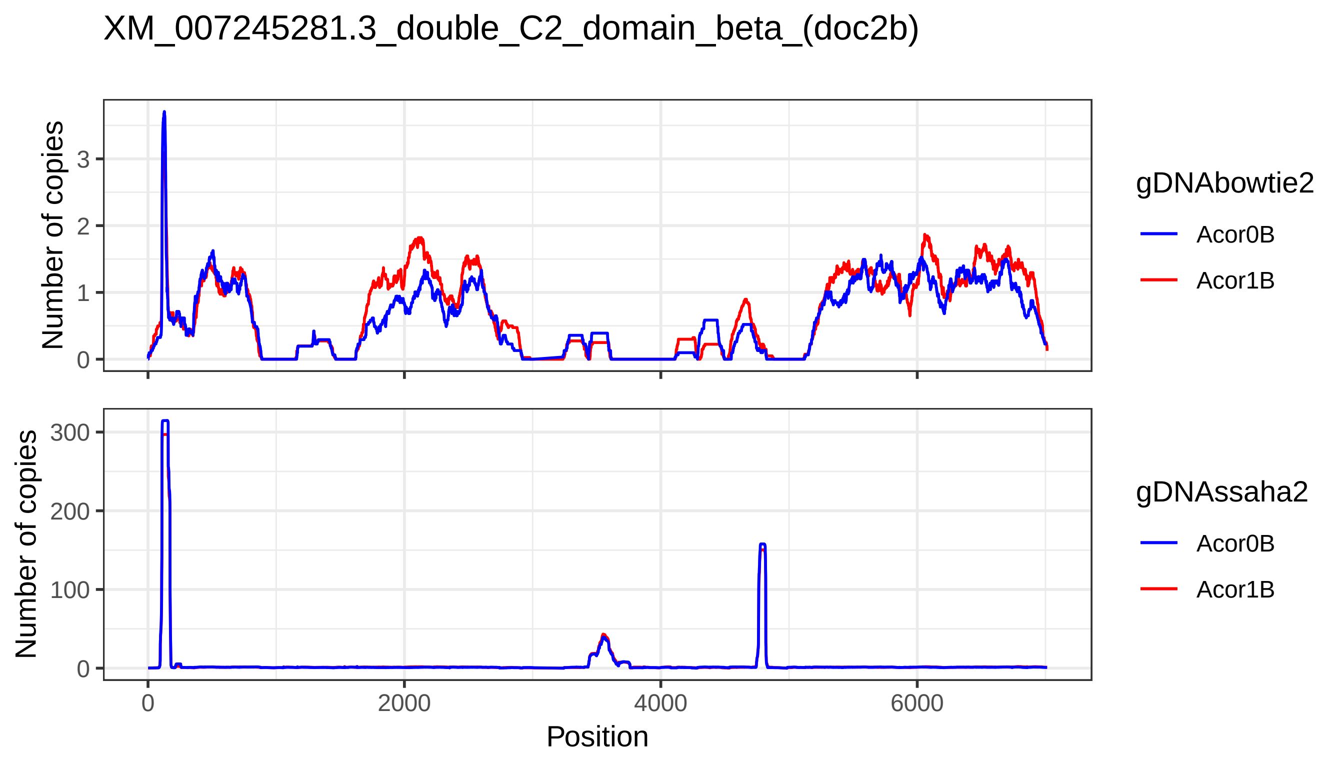
XM_007245281.3_double_C2_domain_beta_(doc2b)
Number of copies
0
1
2
3
gDNAbowtie2
Acor0B
Acor1B
Number of copies
0
100
200
300
gDNAssaha2
Acor0B
Acor1B
0
2000
4000
6000
Position

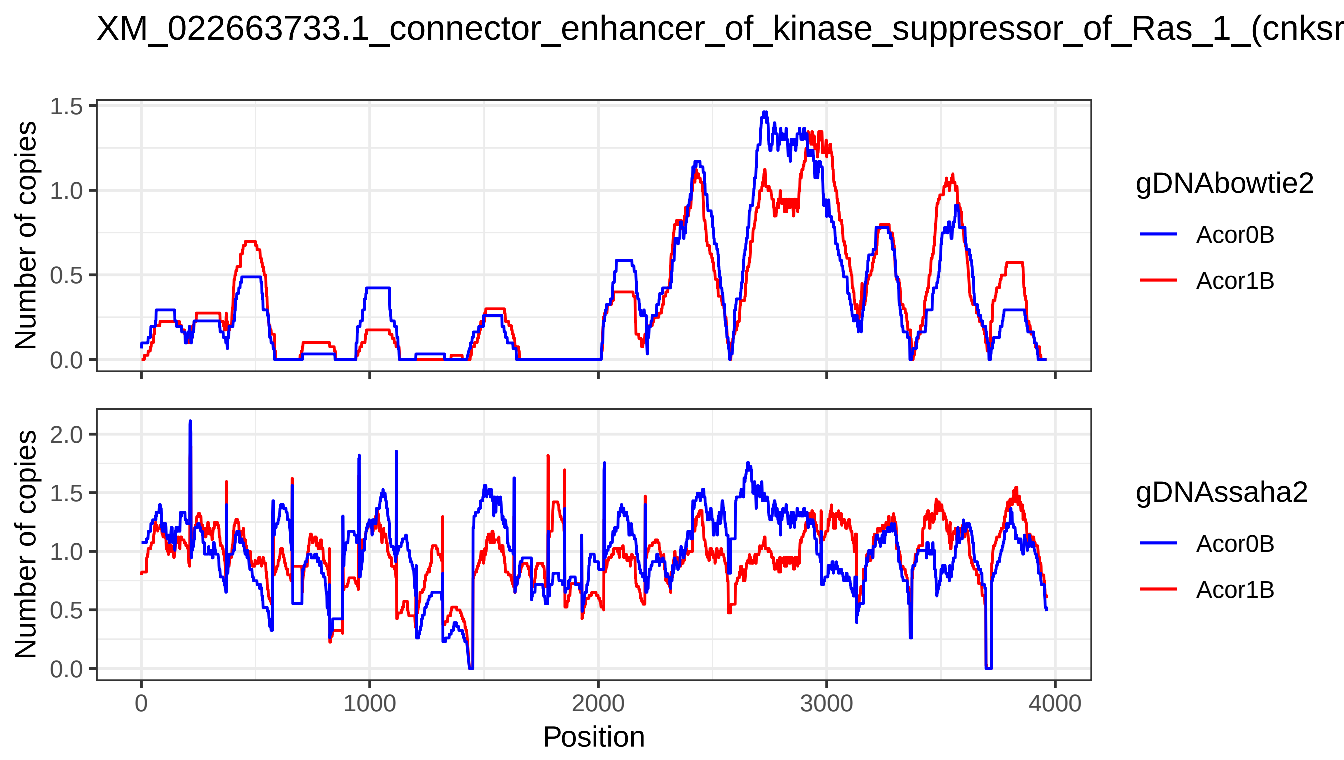

XM_022663733.1_connector_enhancer_of_kinase_suppressor_of_Ras_1_(cnksr
Number of copies
1.5
1.0
0.5
0.0
2.0
1.5
1.0
0.5
0.0
0
1000
2000
3000
4000
Position
gDNAbowtie2
Acor0B
Acor1B
gDNAssaha2
Acor0B
Acor1B

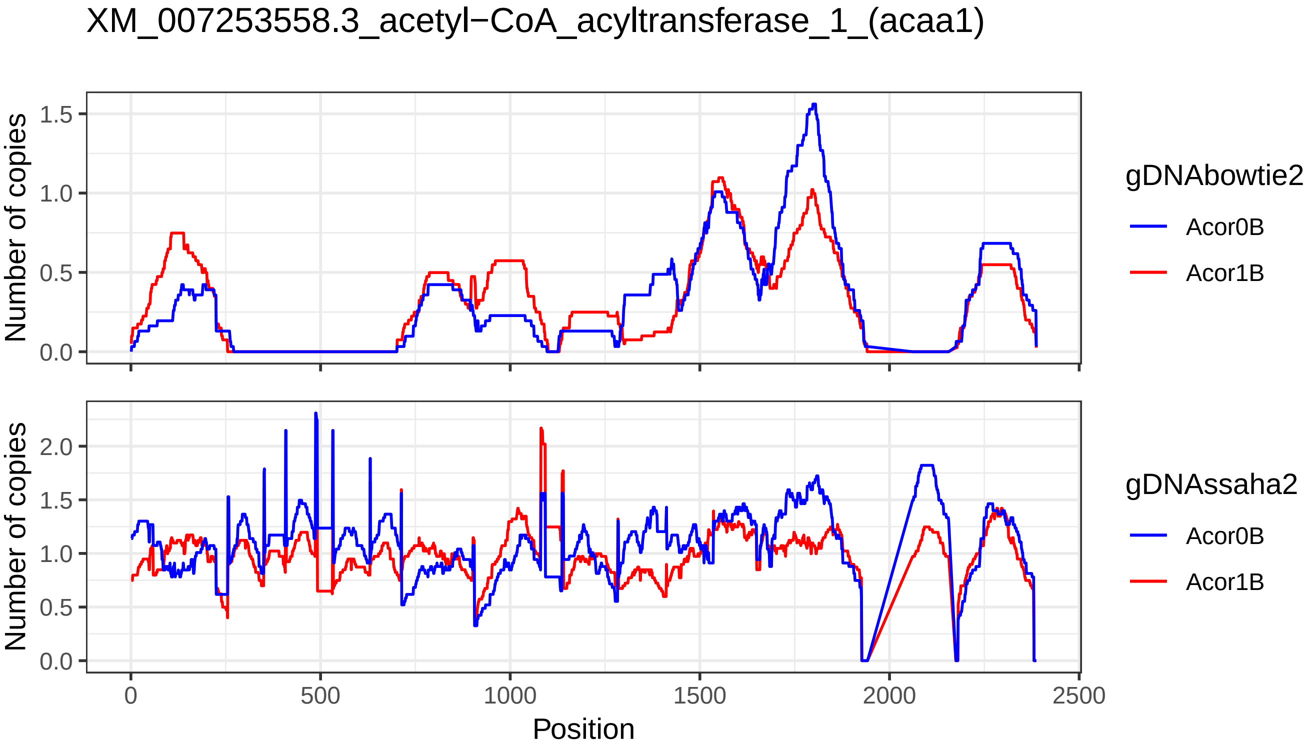

XM_007253558.3_acetyl−CoA_acyltransferase_1_(acaa1)
Number of copies
1.5
1.0
0.5
0.0
gDNAbowtie2
Acor0B
Acor1B
Number of copies
2.0
1.5
1.0
0.5
0.0
gDNAssaha2
Acor0B
Acor1B
0
500
1000
1500
2000
2500
Position

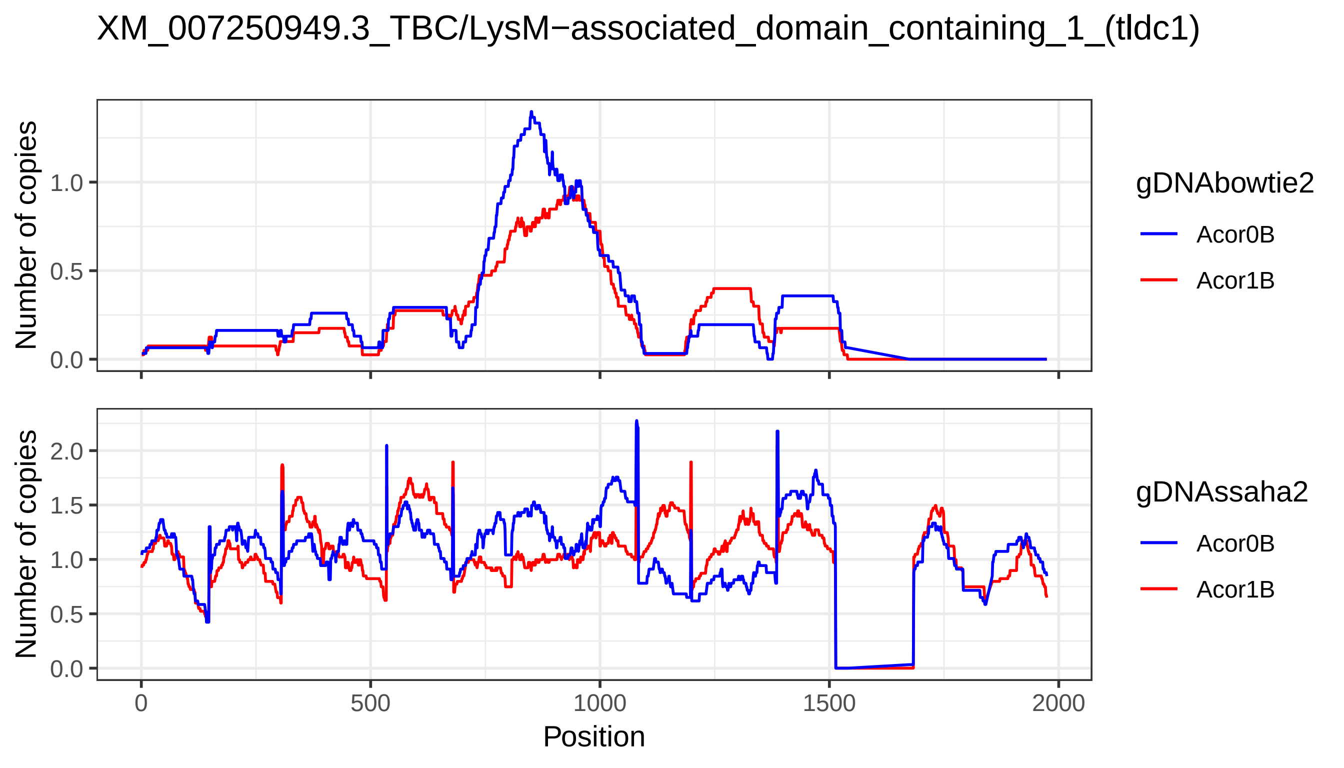
XM_007250949.3_TBC/LysM−associated_domain_containing_1_(tldc1)
Number of copies
1.0
0.5
0.0
gDNAbowtie2
Acor0B
Acor1B
Number of copies
2.0
1.5
1.0
0.5
0.0
gDNAssaha2
Acor0B
Acor1B
0
500
1000
1500
2000
Position

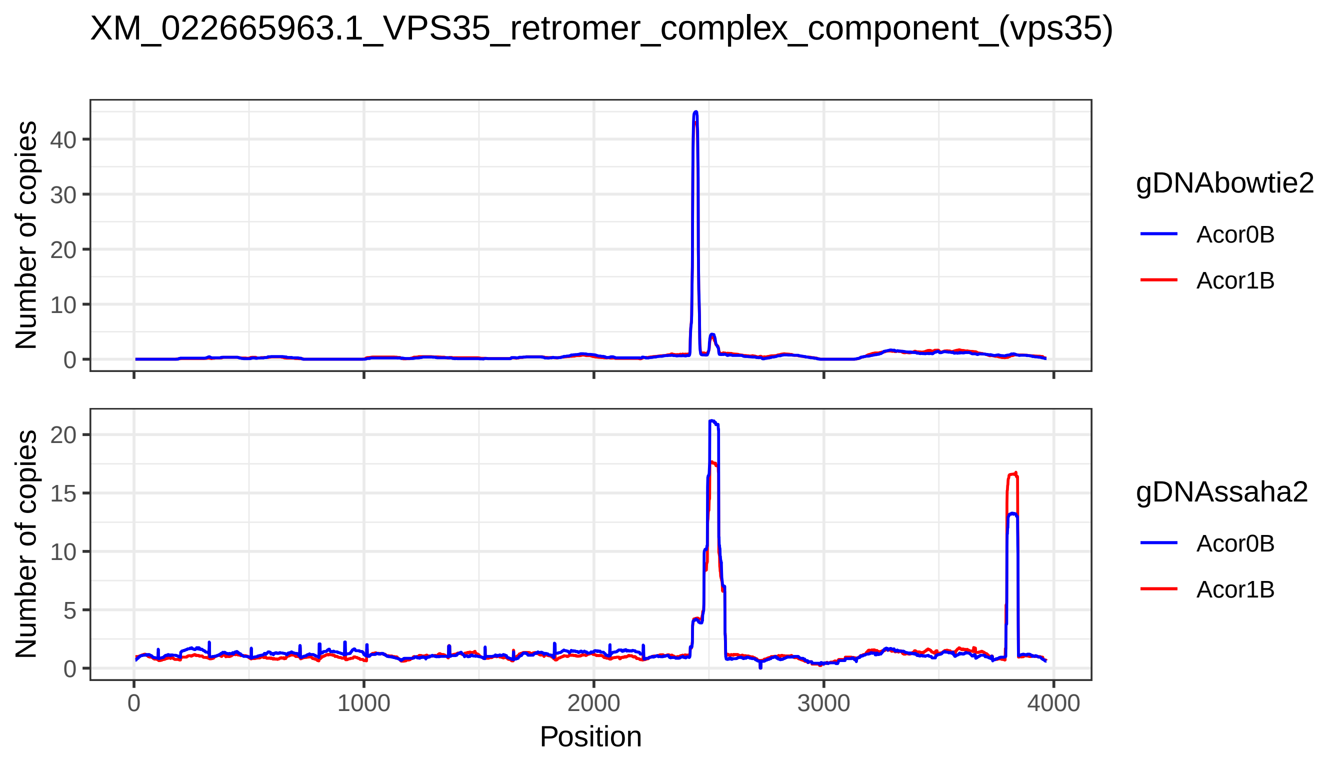

XM_022665963.1_VPS35_retromer_complex_component_(vps35)
Number of copies
40
30
20
10
0
gDNAbowtie2
Acor0B
Acor1B
Number of copies
20
15
10
5
0
gDNAssaha2
Acor0B
Acor1B
0
1000
2000
3000
4000
Position

XM_015606676.2_chondroitin_sulfate_synthase_3_(chsy3)

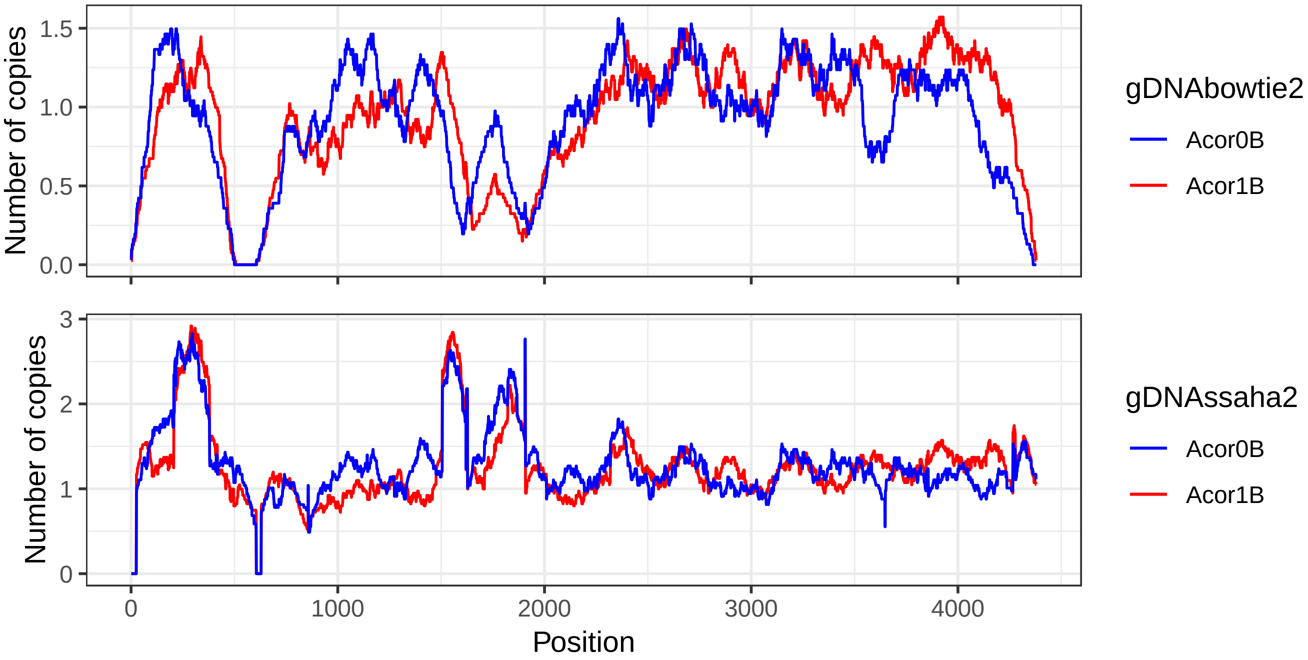

XM_015607831.2_rhomboid_like_3_(rhbdl3)

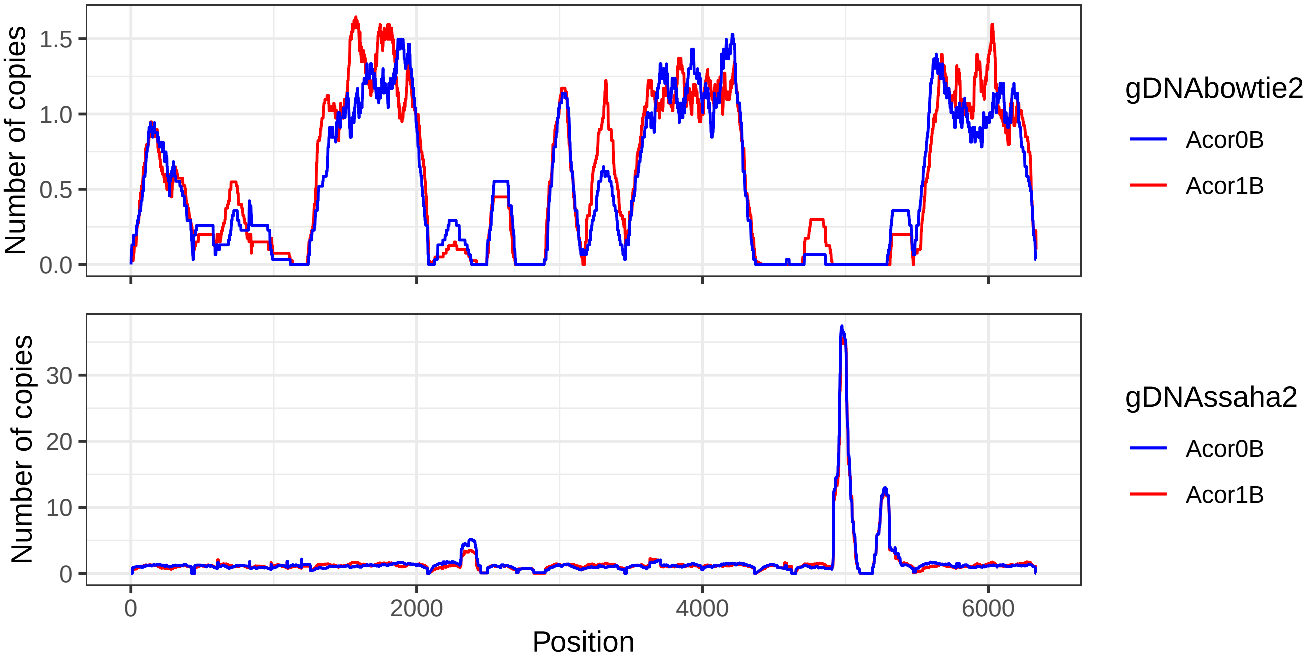

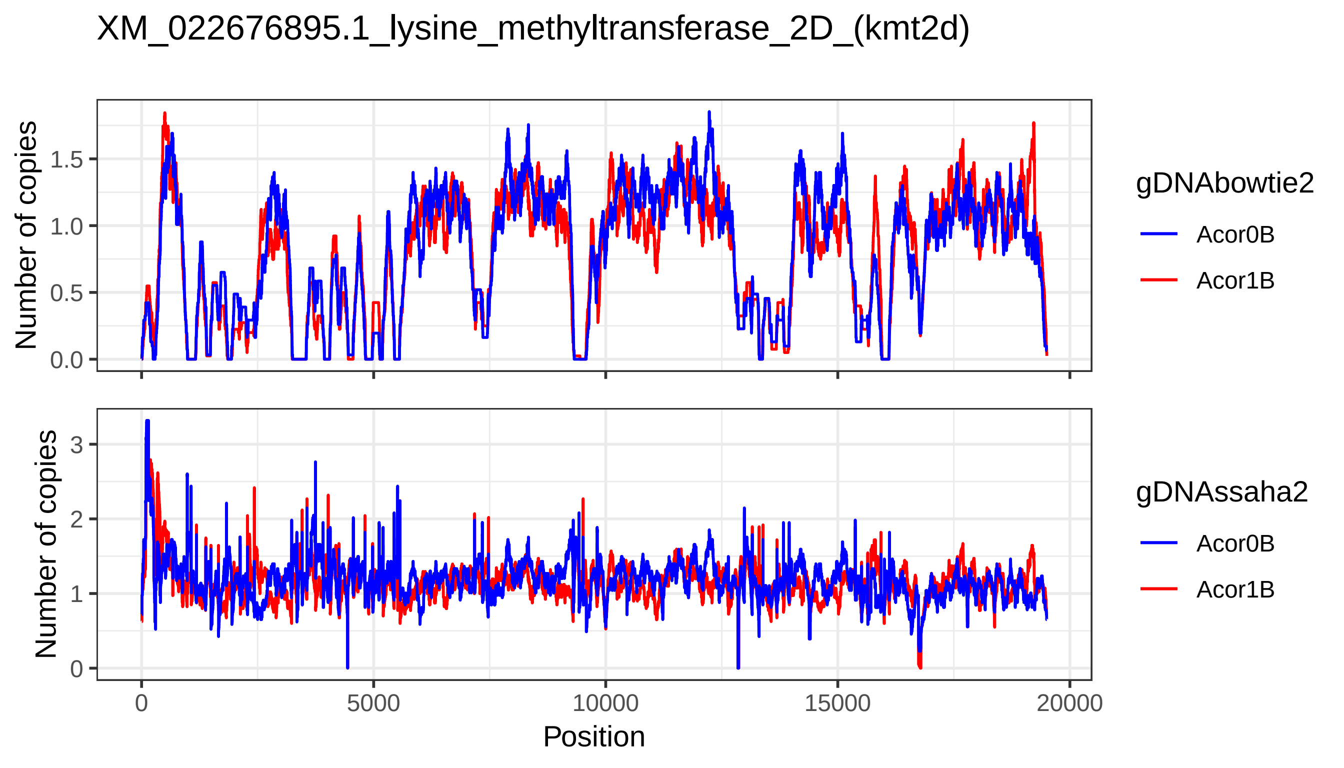
XM_022676895.1_lysine_methyltransferase_2D_(kmt2d)
Number of copies
1.5
1.0
0.5
0.0
3
2
1
0
0
5000
10000
15000
20000
Position
gDNAbowtie2
Acor0B
Acor1B
gDNAssaha2
Acor0B
Acor1B

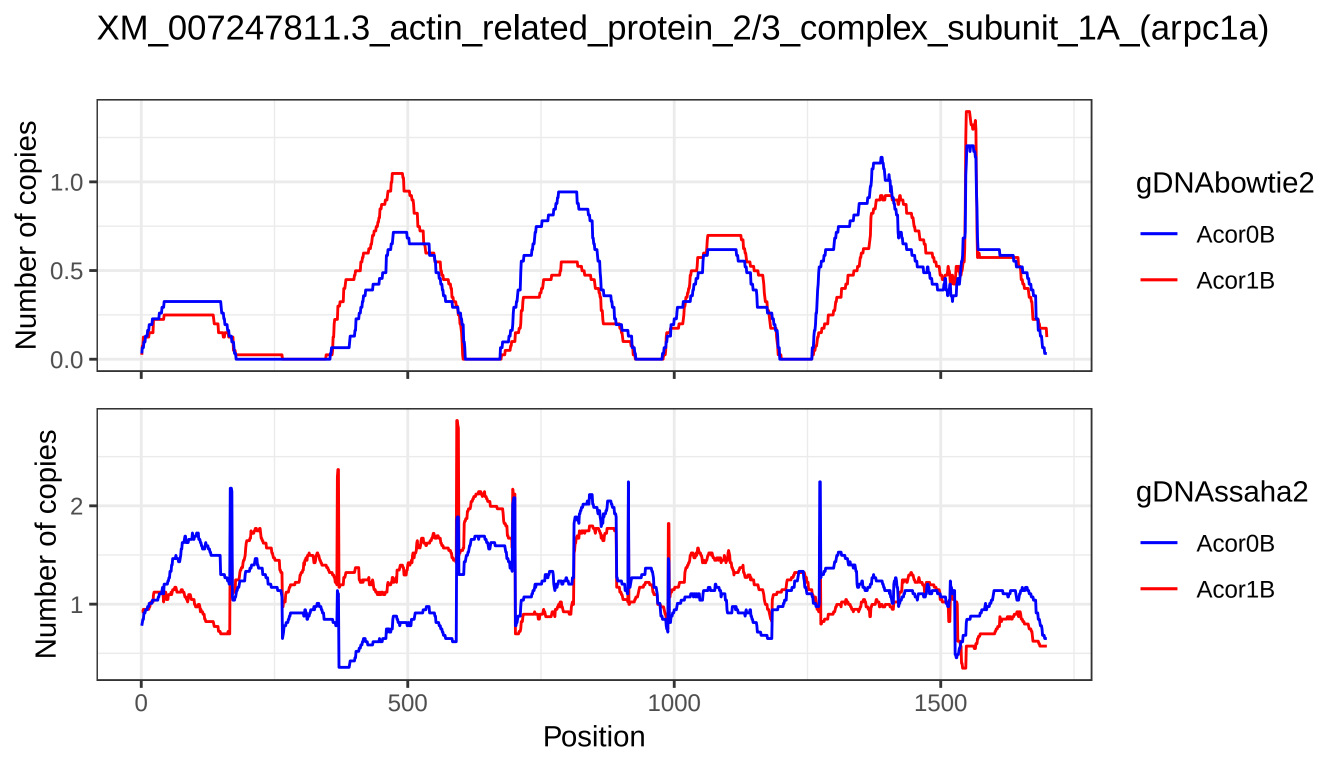
XM_007247811.3_actin_related_protein_2/3_complex_subunit_1A_(arpc1a)
Number of copies
1.0
0.5
0.0
Number of copies
2
1
0
500
1000
1500
Position
gDNAbowtie2
Acor0B
Acor1B
gDNAssaha2
Acor0B
Acor1B

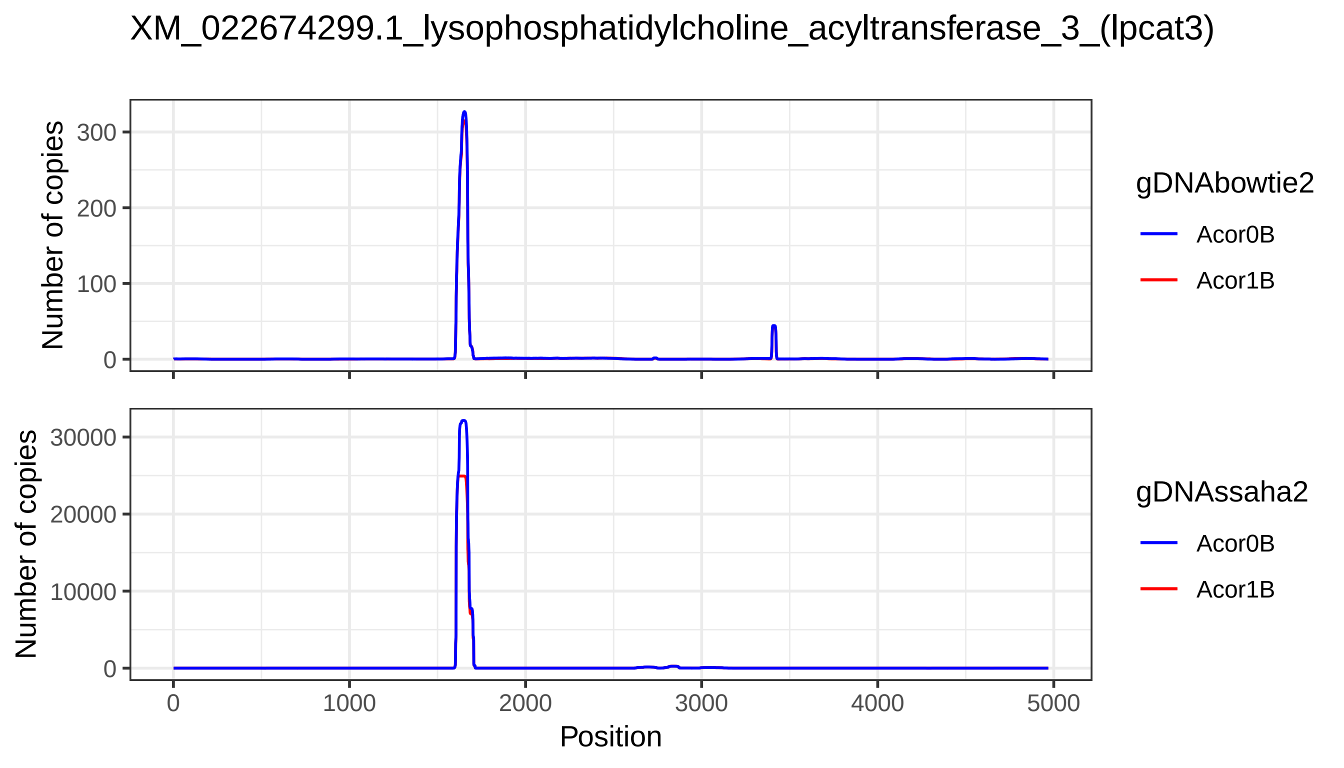

XM_022674299.1_lysophosphatidylcholine_acyltransferase_3_(lpcat3)
Number of copies
300
200
100
0
30000
20000
10000
0
1000
2000
3000
4000
5000
Position
gDNAbowtie2
Acor0B
Acor1B
gDNAssaha2
Acor0B
Acor1B

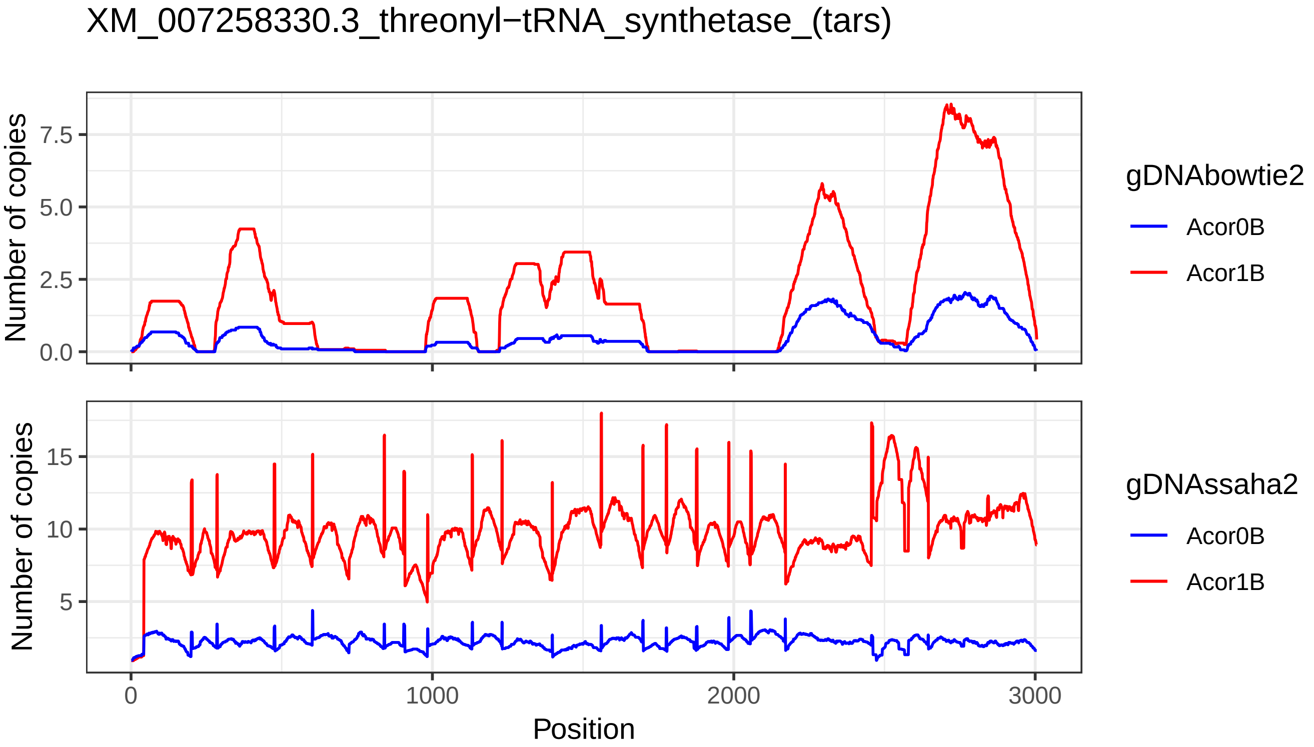
XM_007258330.3_threonyl−tRNA_synthetase_(tars)
Number of copies
0.0
2.5
5.0
7.5
gDNAbowtie2
Acor0B
Acor1B
Number of copies
5
10
15
gDNAssaha2
Acor0B
Acor1B
0
1000
2000
3000
Position

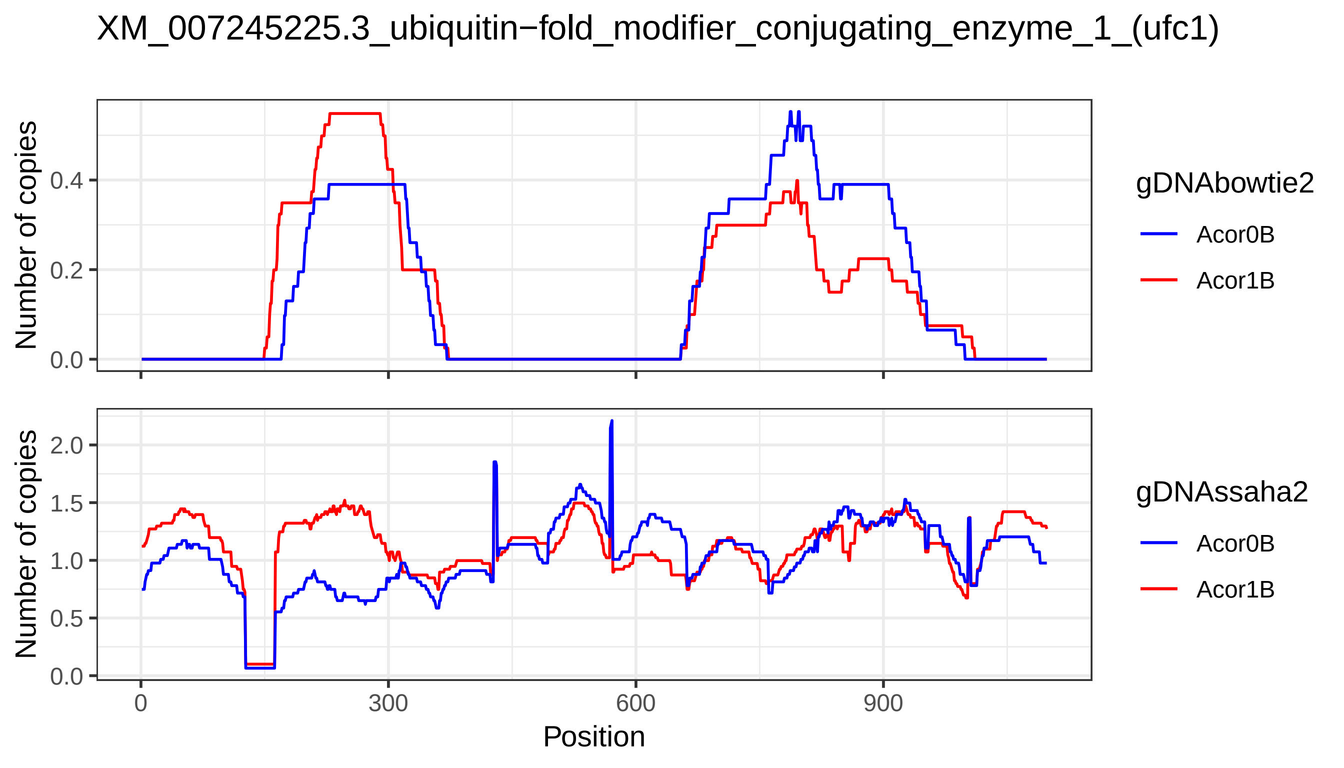
XM_007245225.3_ubiquitin−fold_modifier_conjugating_enzyme_1_(ufc1)
Number of copies
0.0
0.2
0.4
gDNAbowtie2
Acor0B
Acor1B
Number of copies
0.0
0.5
1.0
1.5
2.0
gDNAssaha2
Acor0B
Acor1B
0
300
600
900
Position

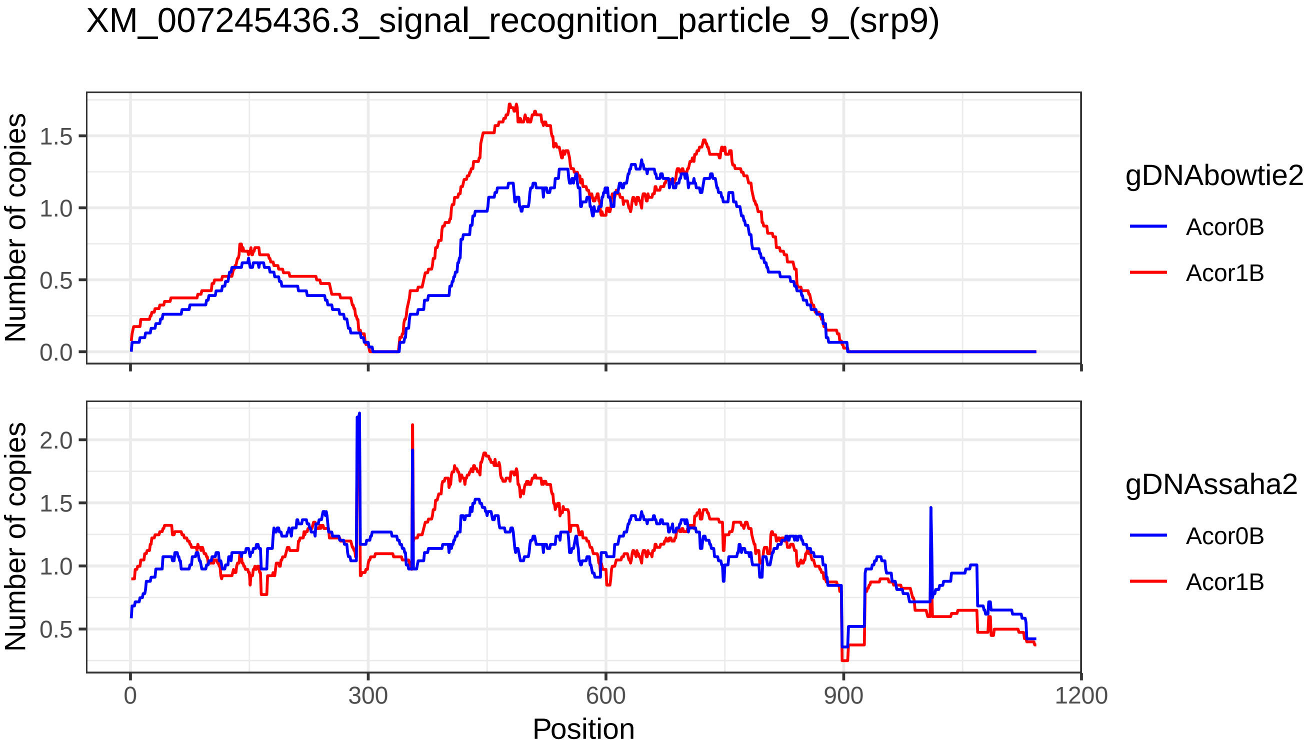
XM_007245436.3_signal_recognition_particle_9_(srp9)
Number of copies
1.5
1.0
0.5
0.0
gDNAbowtie2
Acor0B
Acor1B
2.0
1.5
1.0
0.5
gDNAssaha2
0
300
600
900
1200
Position

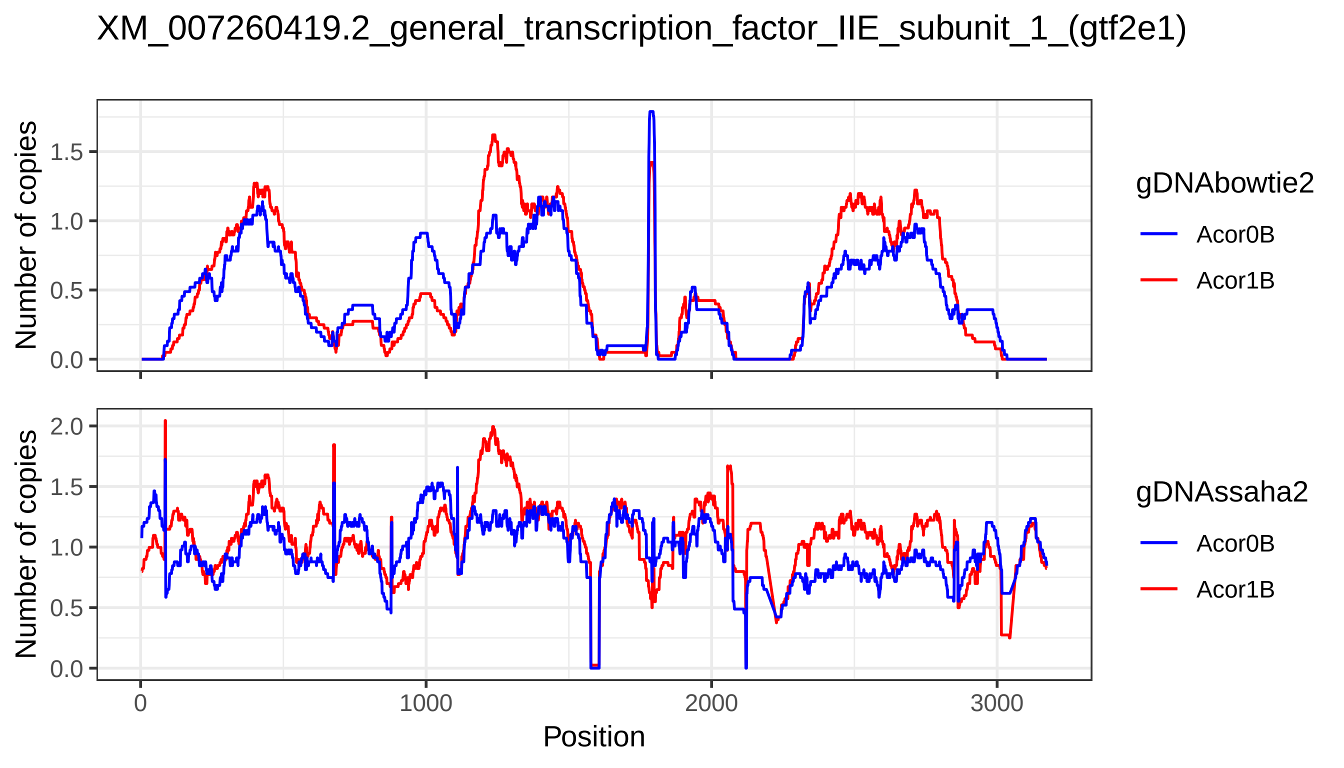
XM_007260419.2_general_transcription_factor_IIE_subunit_1_(gtf2e1)
Number of copies
Number of copies
Position
gDNAbowtie2
Acor0B
Acor1B
gDNAssaha2
Acor0B
Acor1B
0.0
0.5
1.0
1.5
2.0
0
1000
2000
3000

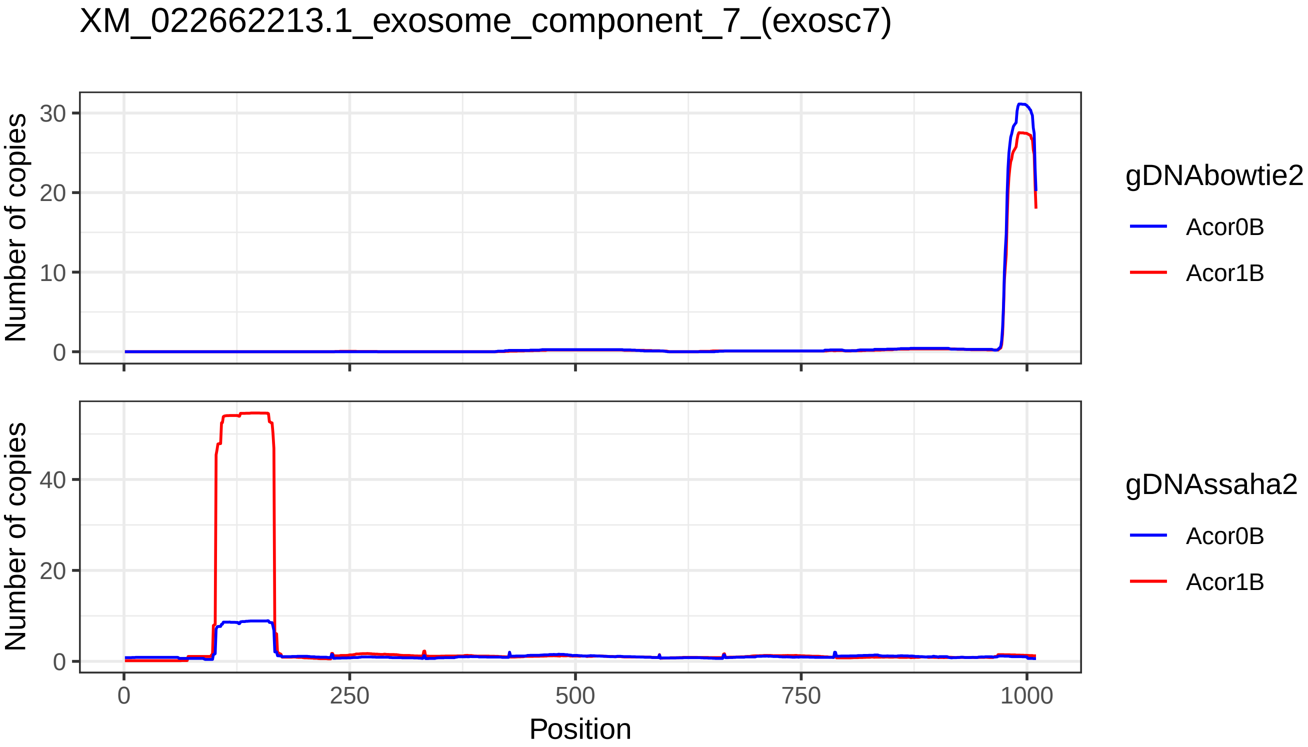
XM_022662213.1_exosome_component_7_(exosc7)
Number of copies
30
20
10
0
40
20
0
0
250
500
750
1000
Position
gDNAbowtie2
Acor0B
Acor1B
gDNAssaha2
Acor0B
Acor1B
