# Supplementary material for: Irreproducible results and unsupported conclusions in Ahmad et al. [BMC genomics (2020) 21:656]
Source: BMC Genomics. 2023 Dec 18;24:778. doi: 10.1186/s12864-023-09883-4 (PMC10726643; doi:10.1186/s12864-023-09883-4)
Supplement: Supplementary file 5 — Coverage across the 61 EBI contigs in 0B and 1B gDNA libraries of A. correntinus comparing the Bowtie2 and the SSAHA2 mappings. Average values and 1B/0B ratios are shown in Supplementary File 3. [file 12864_2023_9883_MOESM5_ESM.pdf]

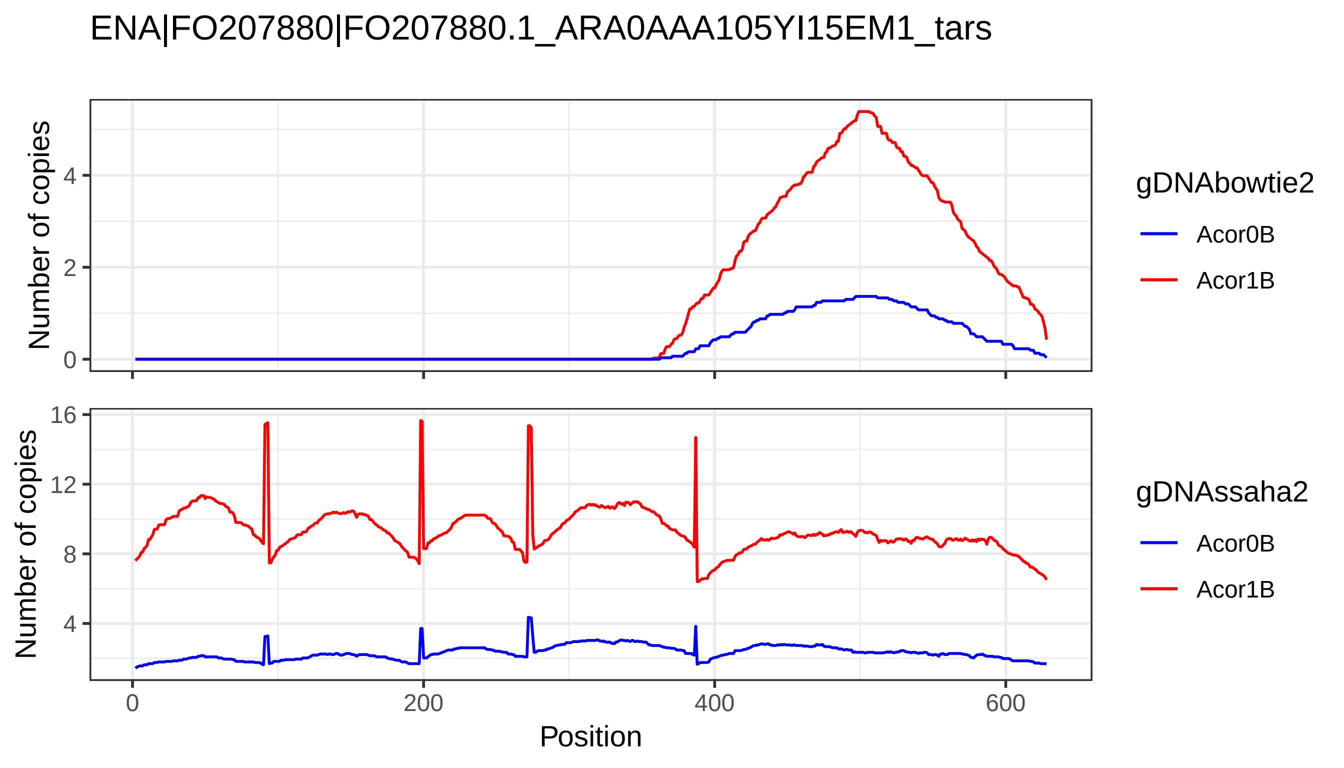
ENA|FO207880|FO207880.1_ARA0AAA105YI15EM1_tars
Number of copies
0
2
4
16
12
8
4
0
200
400
600
Position
gDNAbowtie2
Acor0B
Acor1B
gDNAssaha2
Acor0B
Acor1B

ENA|FO214127|FO214127.1_ARA0AAA12YF15EM1_exosc7

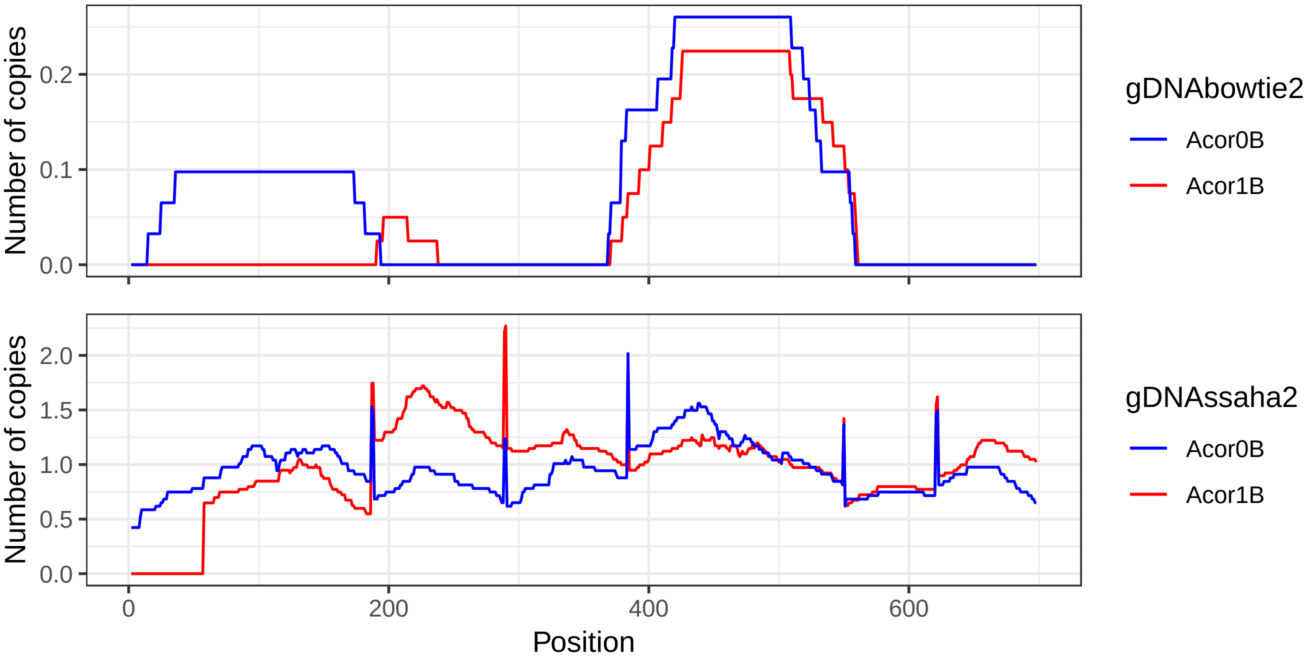

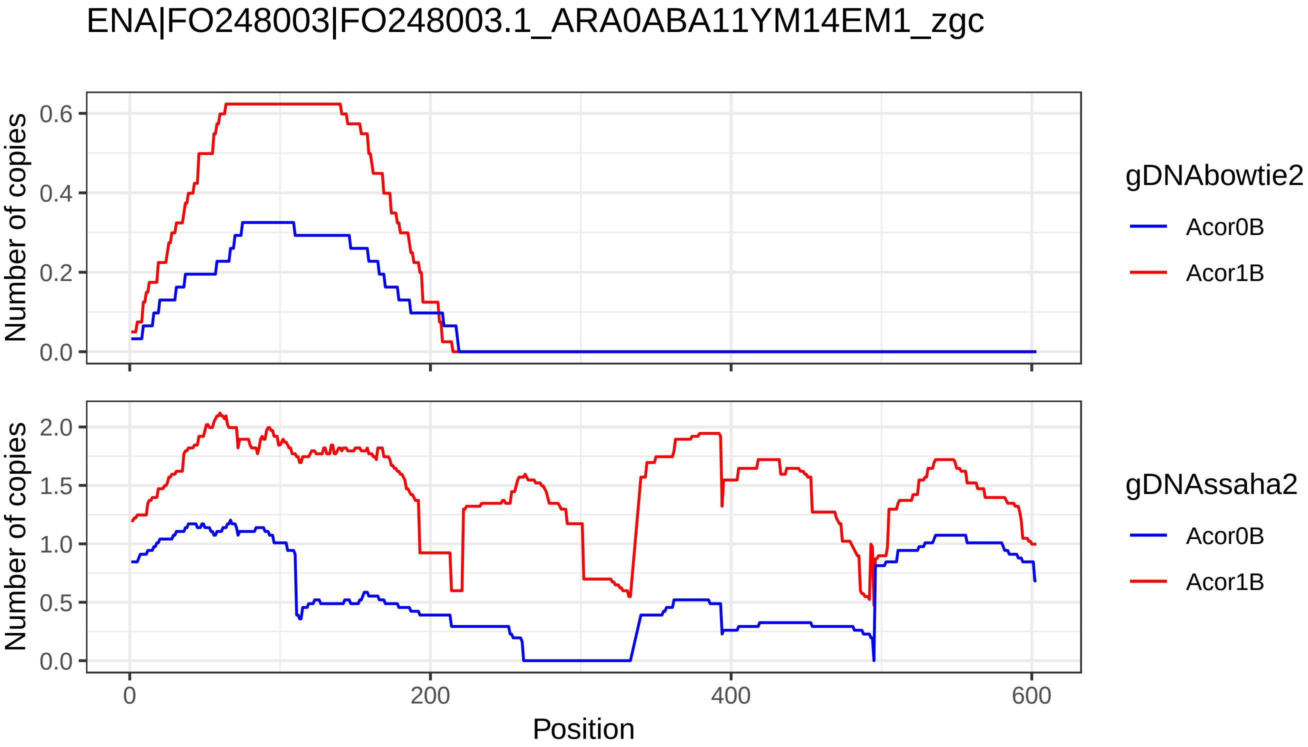
ENA|FO248003|FO248003.1_ARA0ABA11YM14EM1_zgc
Number of copies
0.0
0.2
0.4
0.6
gDNAbowtie2
Acor0B
Acor1B
Number of copies
0.0
0.5
1.0
1.5
2.0
gDNAssaha2
Acor0B
Acor1B
0
200
400
600
Position

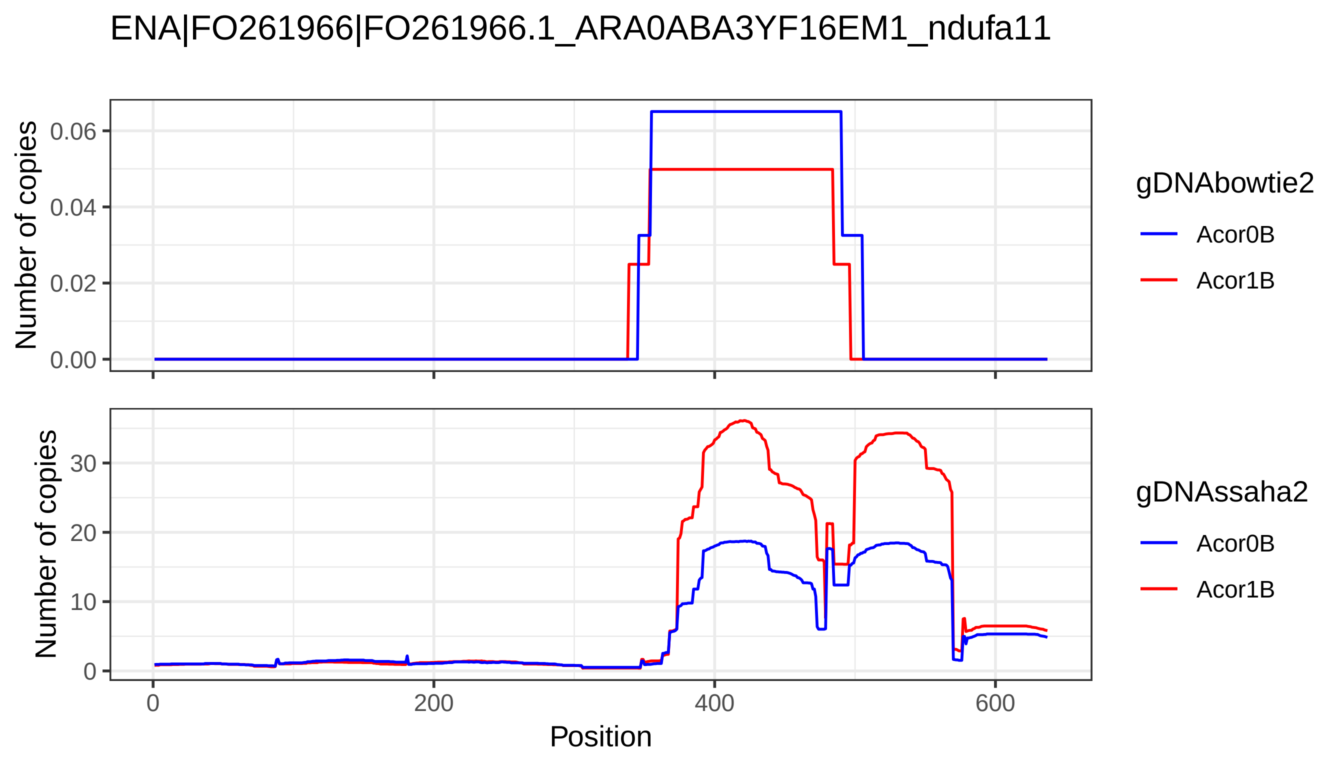

ENA|FO261966|FO261966.1_ARA0ABA3YF16EM1_ndufa11
Number of copies
0.06
0.04
0.02
0.00
gDNAbowtie2
Acor0B
Acor1B
Number of copies
30
20
10
0
gDNAssaha2
Acor0B
Acor1B
0
200
400
600
Position

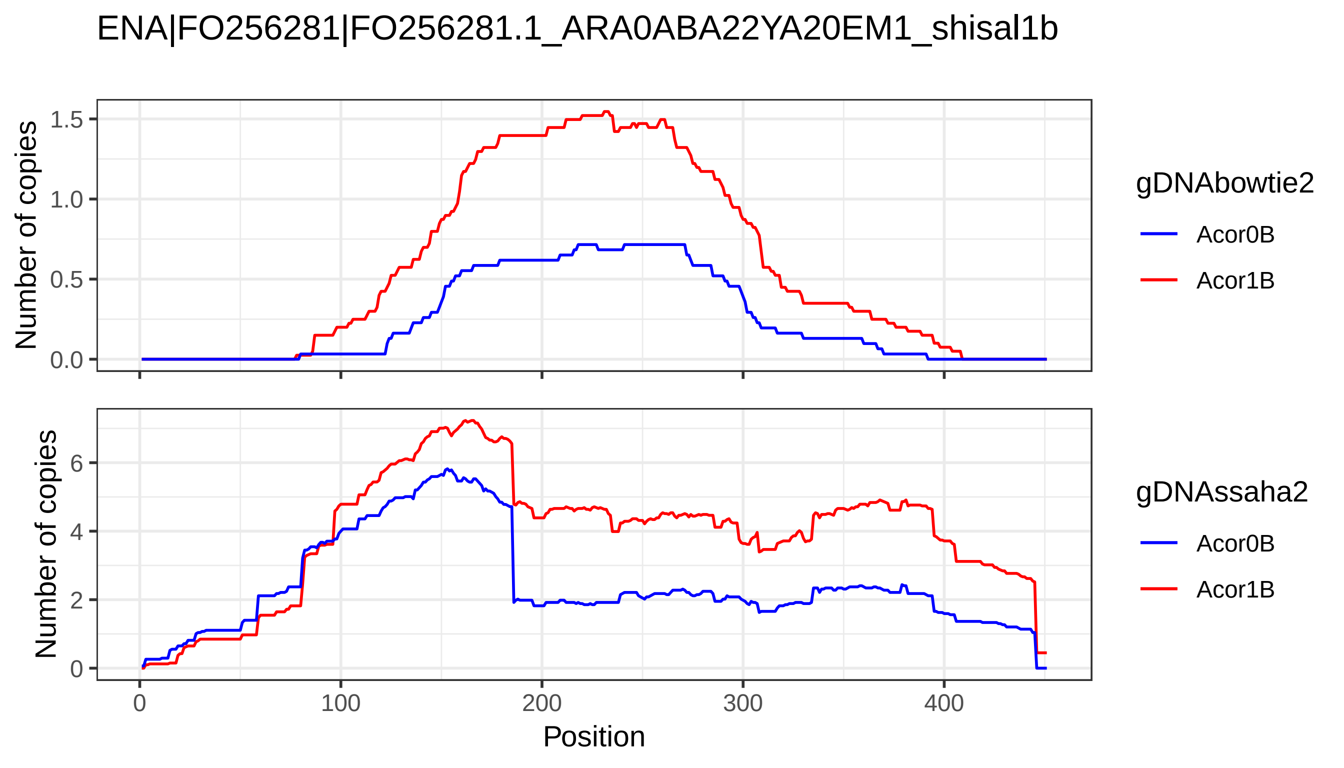

ENA|FO256281|FO256281.1_ARA0ABA22YA20EM1_shisal1b
Number of copies
1.5
1.0
0.5
0.0
gDNAbowtie2
Acor0B
Acor1B
Number of copies
6
4
2
0
gDNAssaha2
Acor0B
Acor1B
0
100
200
300
400
Position

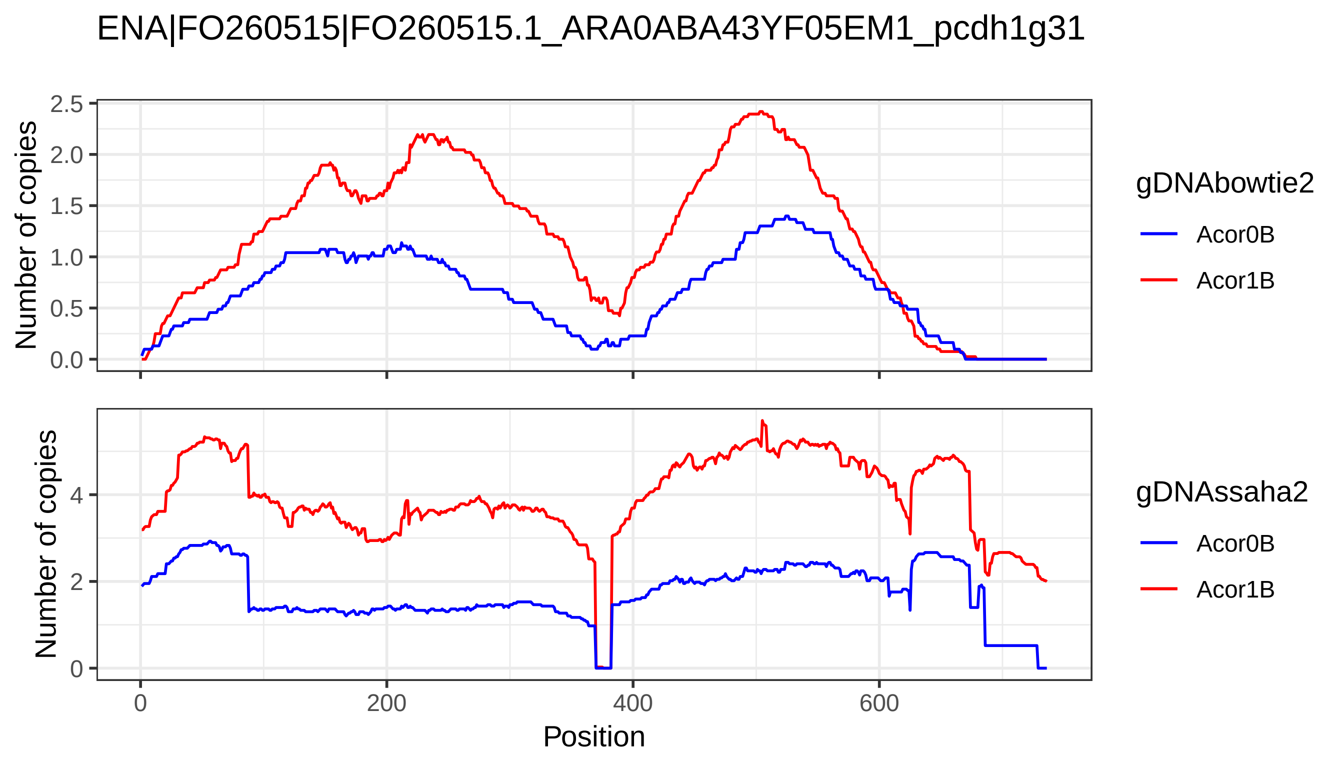
ENA|FO260515|FO260515.1_ARA0ABA43YF05EM1_pcdh1g31
Number of copies
2.5
2.0
1.5
1.0
0.5
0.0
gDNAbowtie2
Acor0B
Acor1B
Number of copies
4
2
0
gDNAssaha2
Acor0B
Acor1B
0
200
400
600
Position

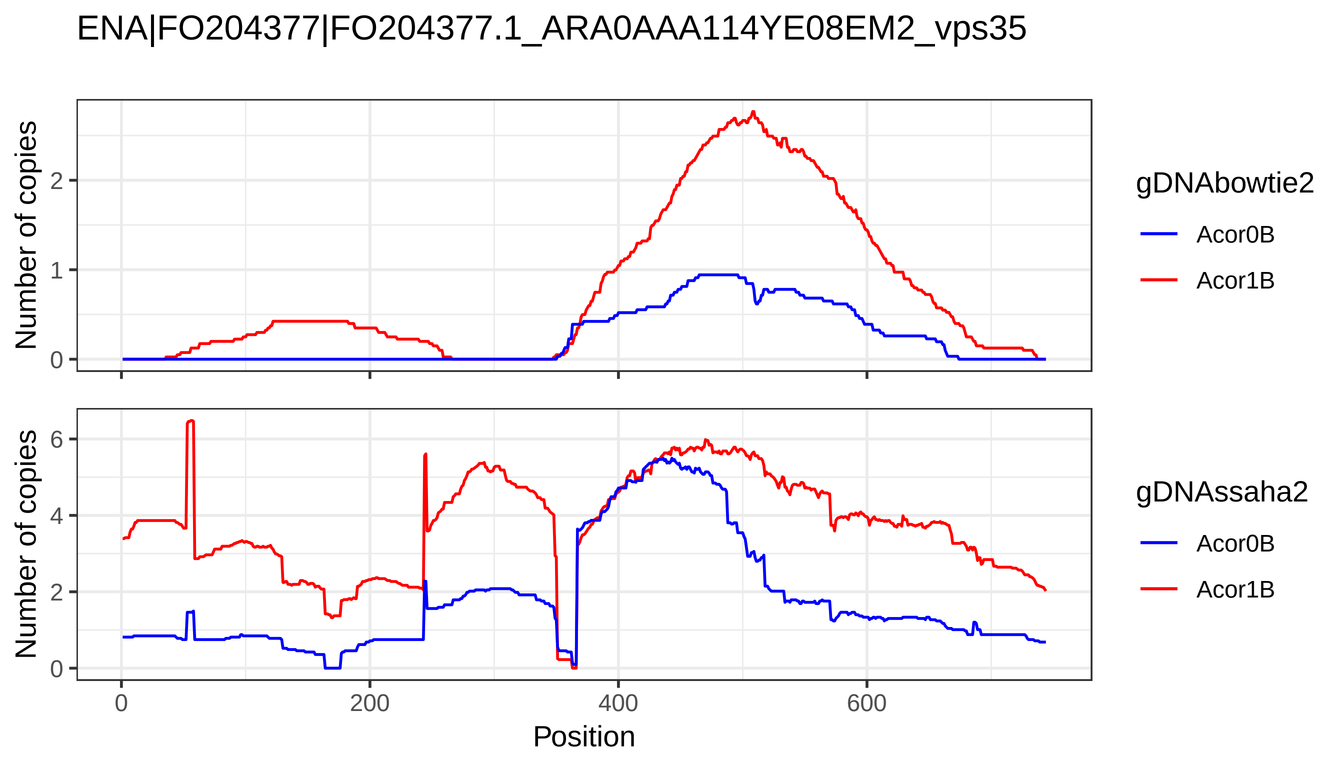

ENA|FO204377|FO204377.1_ARA0AAA114YE08EM2_vps35
Number of copies
Number of copies
Position
0
1
2
0
2
4
6
0
200
400
600
gDNAbowtie2
Acor0B
Acor1B
gDNAssaha2
Acor0B
Acor1B

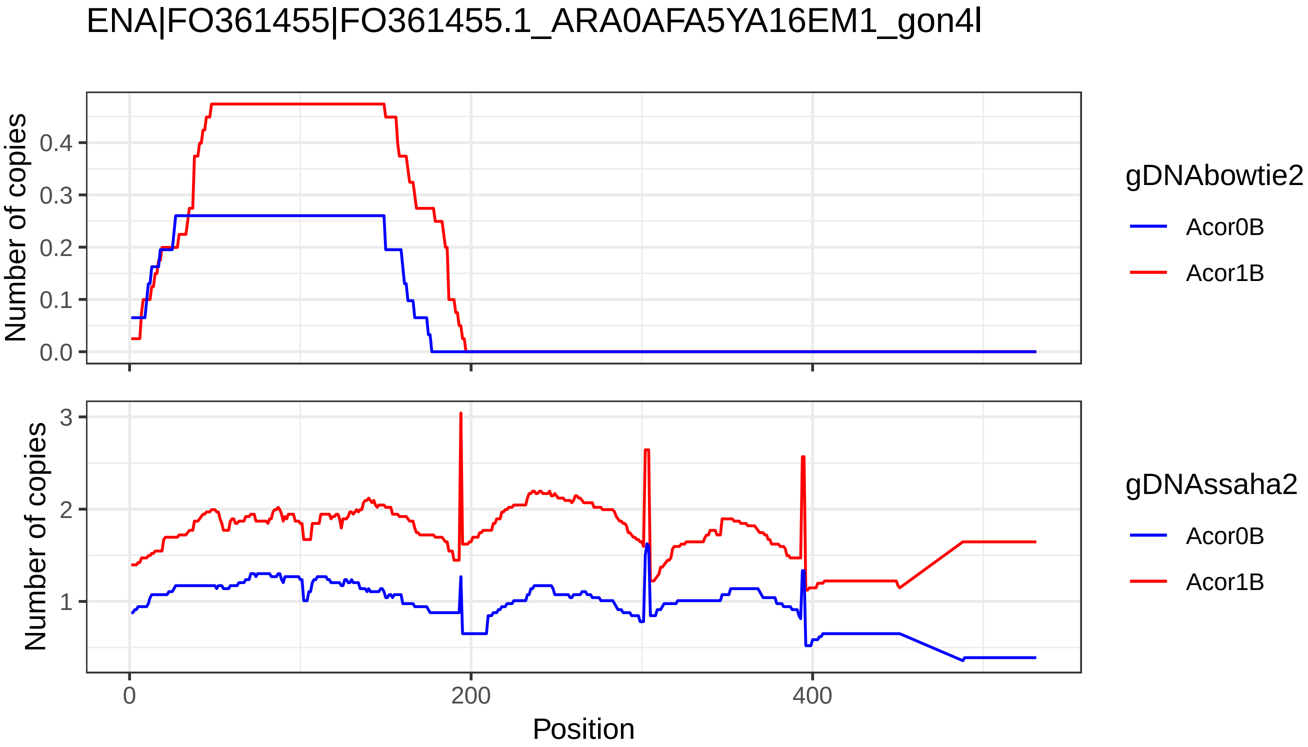

ENA|FO361455|FO361455.1_ARA0AFA5YA16EM1_gon4l
Number of copies
0.0
0.1
0.2
0.3
0.4
gDNAbowtie2
Acor0B
Acor1B
Number of copies
1
2
3
gDNAssaha2
Acor0B
Acor1B
0
200
400
Position

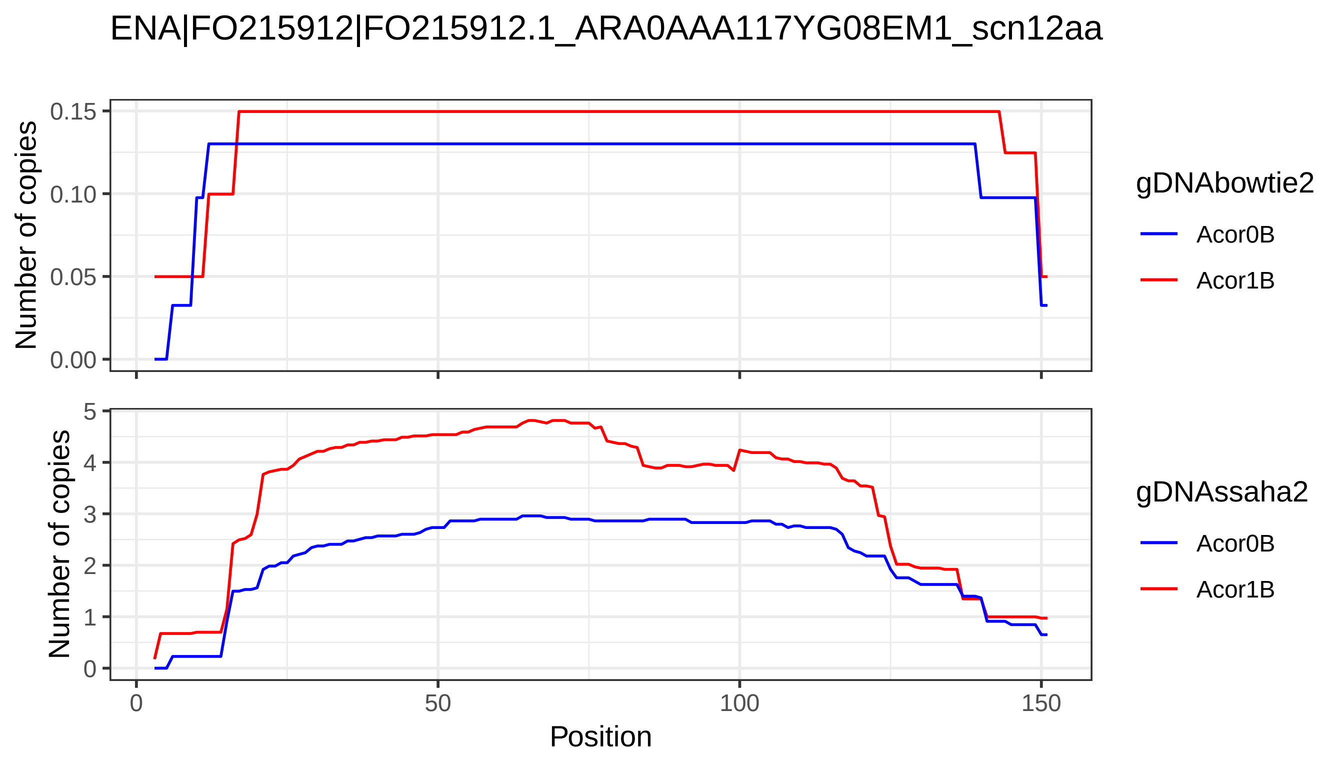
ENA|FO215912|FO215912.1_ARA0AAA117YG08EM1_scn12aa
Number of copies
0.15
0.10
0.05
0.00
gDNAbowtie2
Acor0B
Acor1B
Number of copies
5
4
3
2
1
0
gDNAssaha2
Acor0B
Acor1B
0
50
100
150
Position

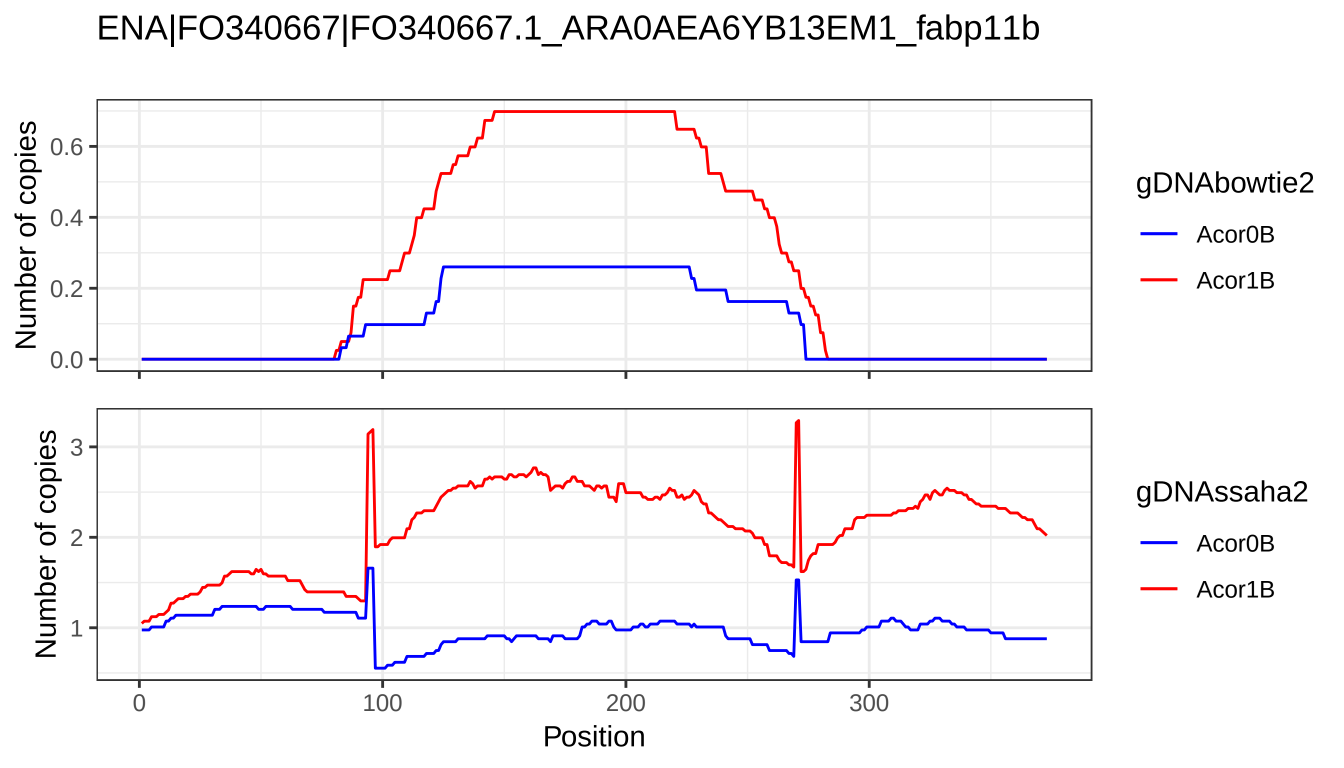

ENA|FO340667|FO340667.1_ARA0AEA6YB13EM1_fabp11b
Number of copies
0.0
0.2
0.4
0.6
gDNAbowtie2
Acor0B
Acor1B
Number of copies
1
2
3
gDNAssaha2
Acor0B
Acor1B
0
100
200
300
Position

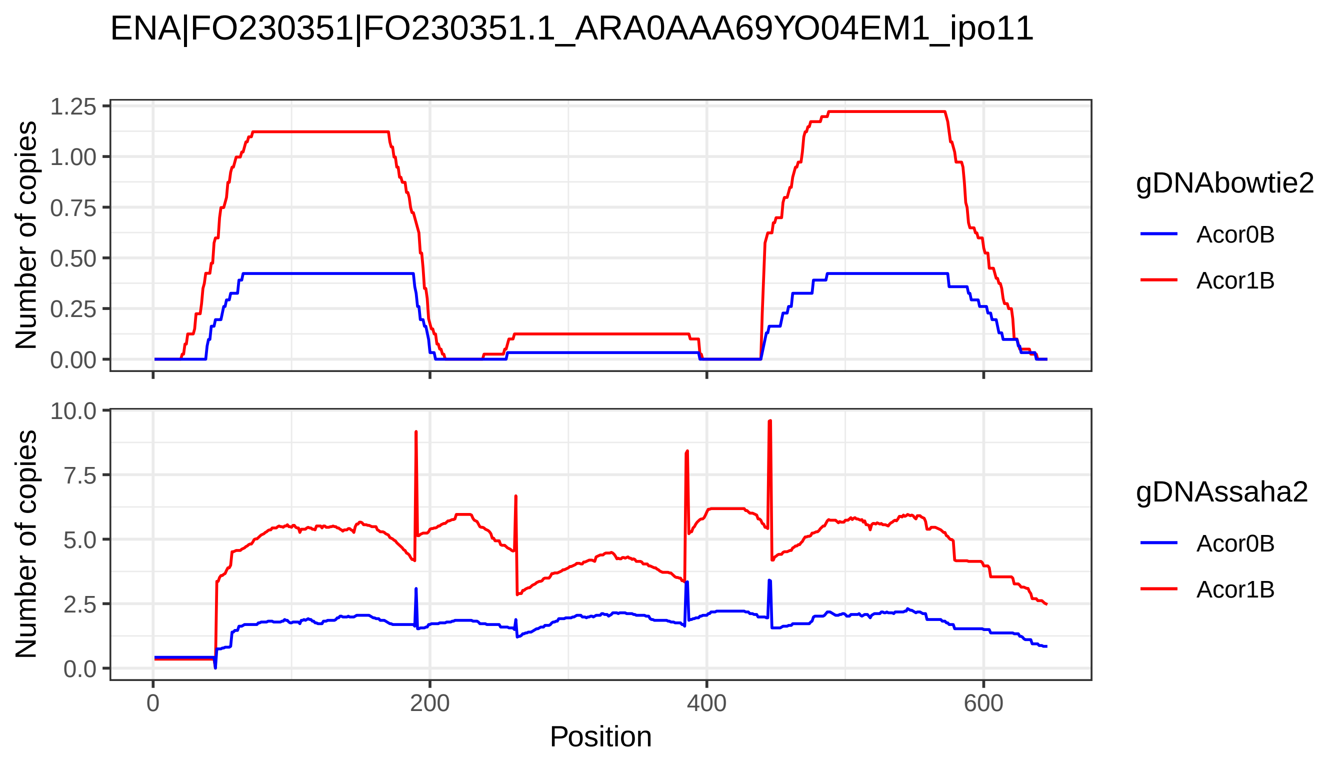
ENA|FO230351|FO230351.1_ARA0AAA69YO04EM1_ipo11
Number of copies
1.25
1.00
0.75
0.50
0.25
0.00
10.0
7.5
5.0
2.5
0.0
0
200
400
600
Position
gDNAbowtie2
Acor0B
Acor1B
gDNAssaha2
Acor0B
Acor1B

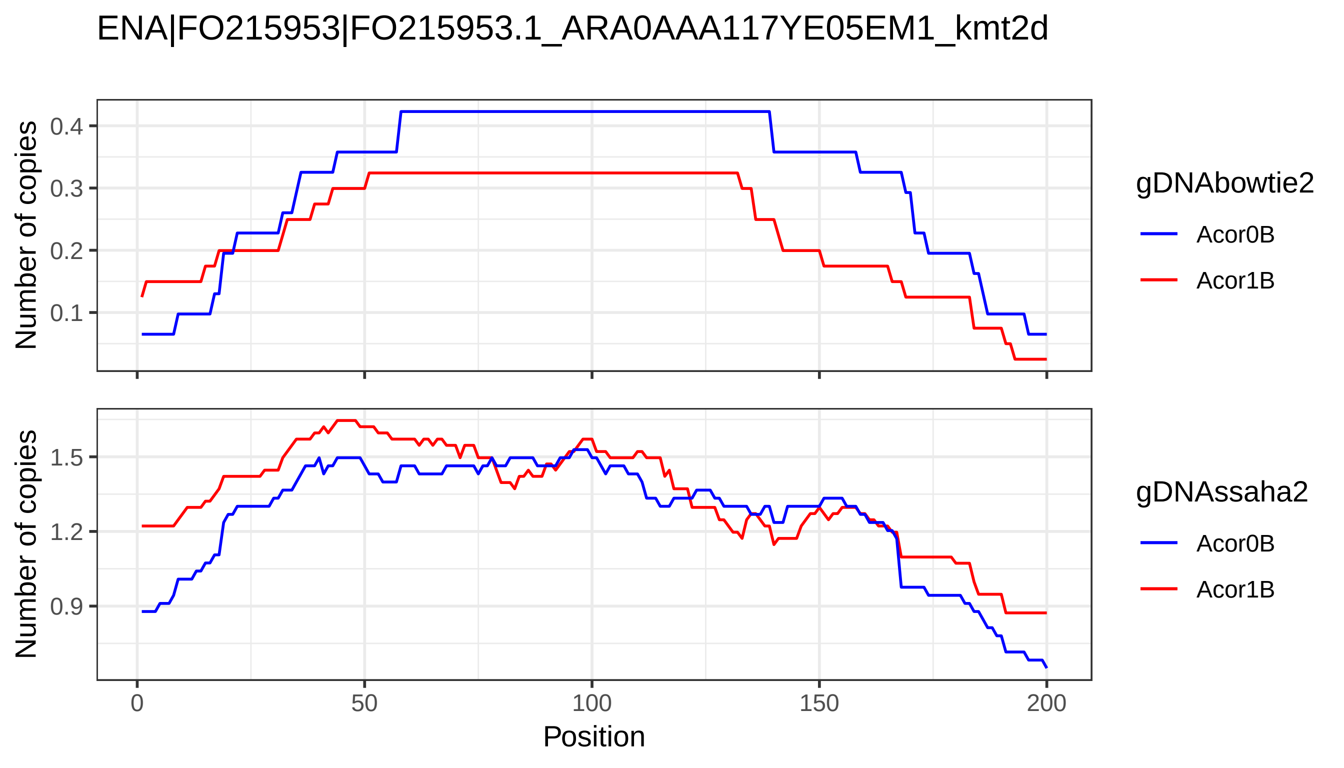
ENA|FO215953|FO215953.1_ARA0AAA117YE05EM1_kmt2d
Number of copies
0.4
0.3
0.2
0.1
gDNAbowtie2
Acor0B
Acor1B
Number of copies
1.5
1.2
0.9
gDNAssaha2
Acor0B
Acor1B
0
50
100
150
200
Position

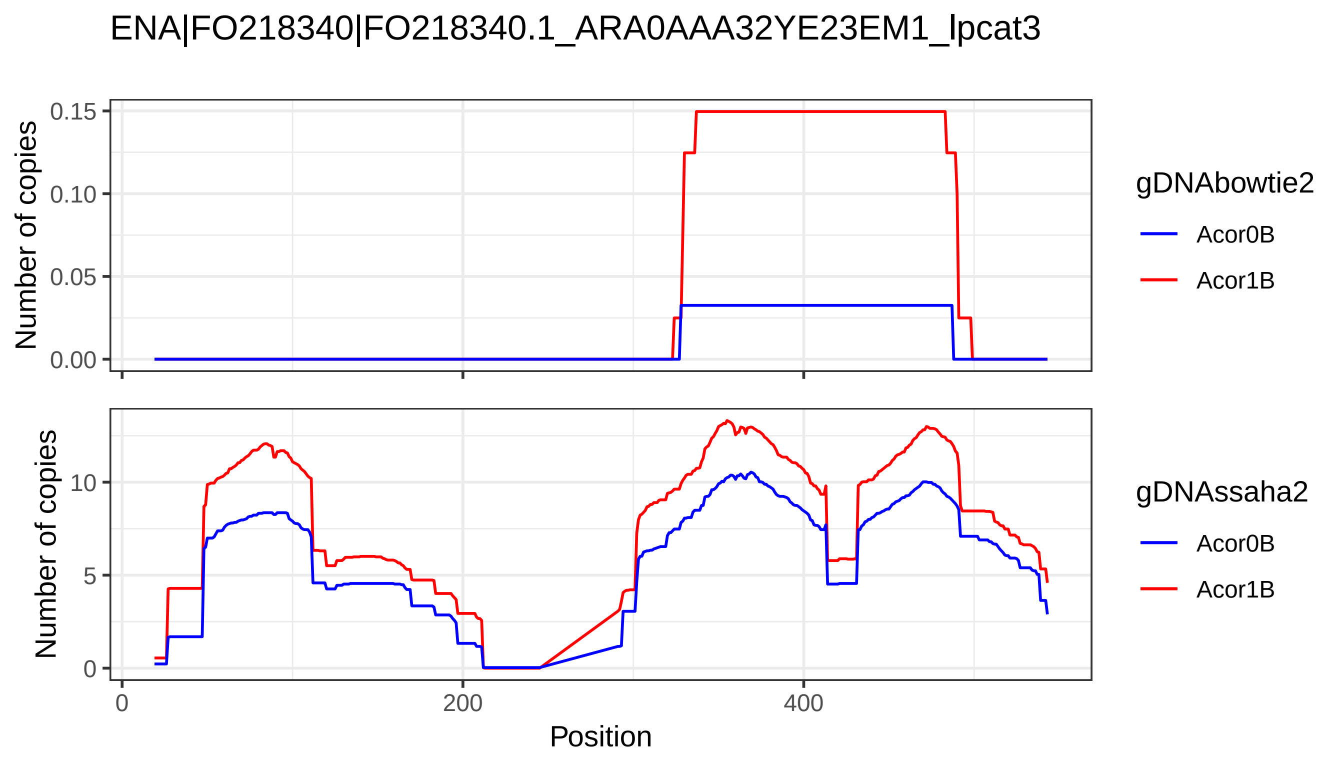
ENA|FO218340|FO218340.1_ARA0AAA32YE23EM1_lpcat3
Number of copies
0.15
0.10
0.05
0.00
Number of copies
10
5
0
0
200
400
Position
gDNAbowtie2
Acor0B
Acor1B
gDNAssaha2
Acor0B
Acor1B

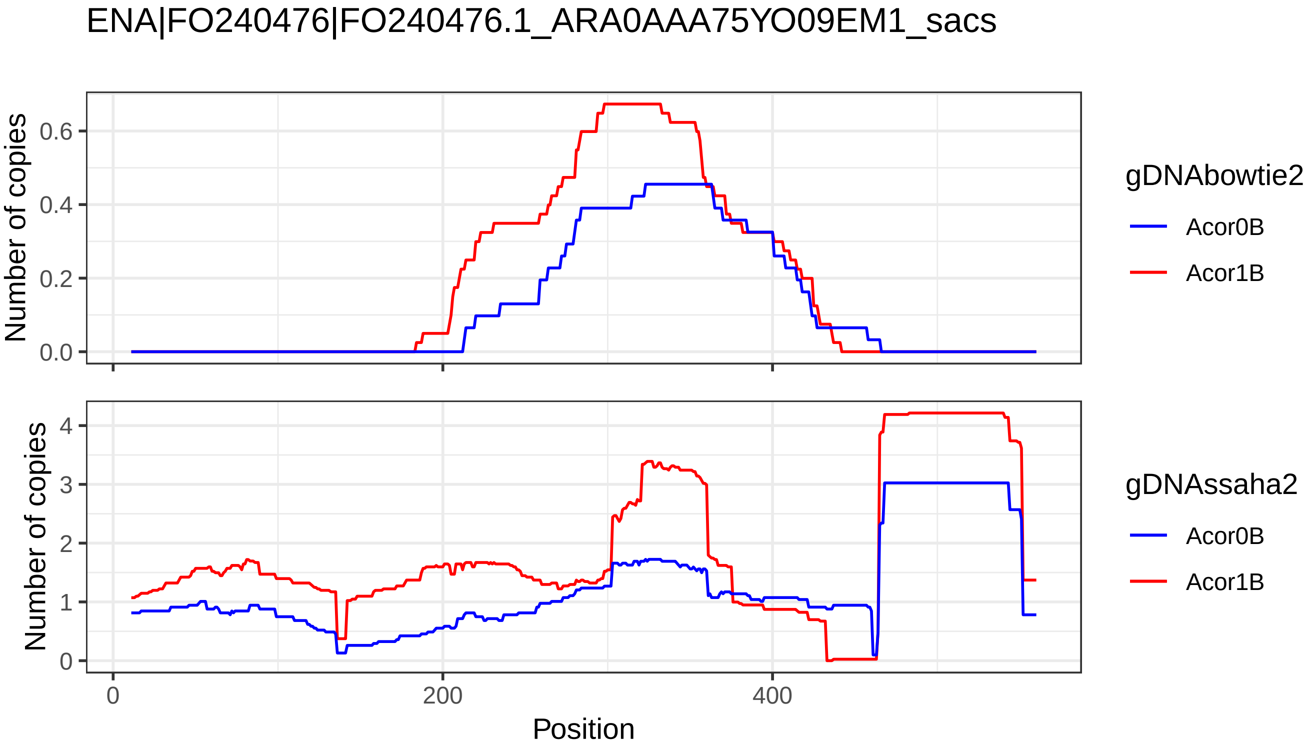
ENA|FO240476|FO240476.1_ARA0AAA75YO09EM1_sacs
Number of copies
0.0
0.2
0.4
0.6
gDNAbowtie2
Acor0B
Acor1B
Number of copies
0
1
2
3
4
gDNAssaha2
Acor0B
Acor1B
0
200
400
Position

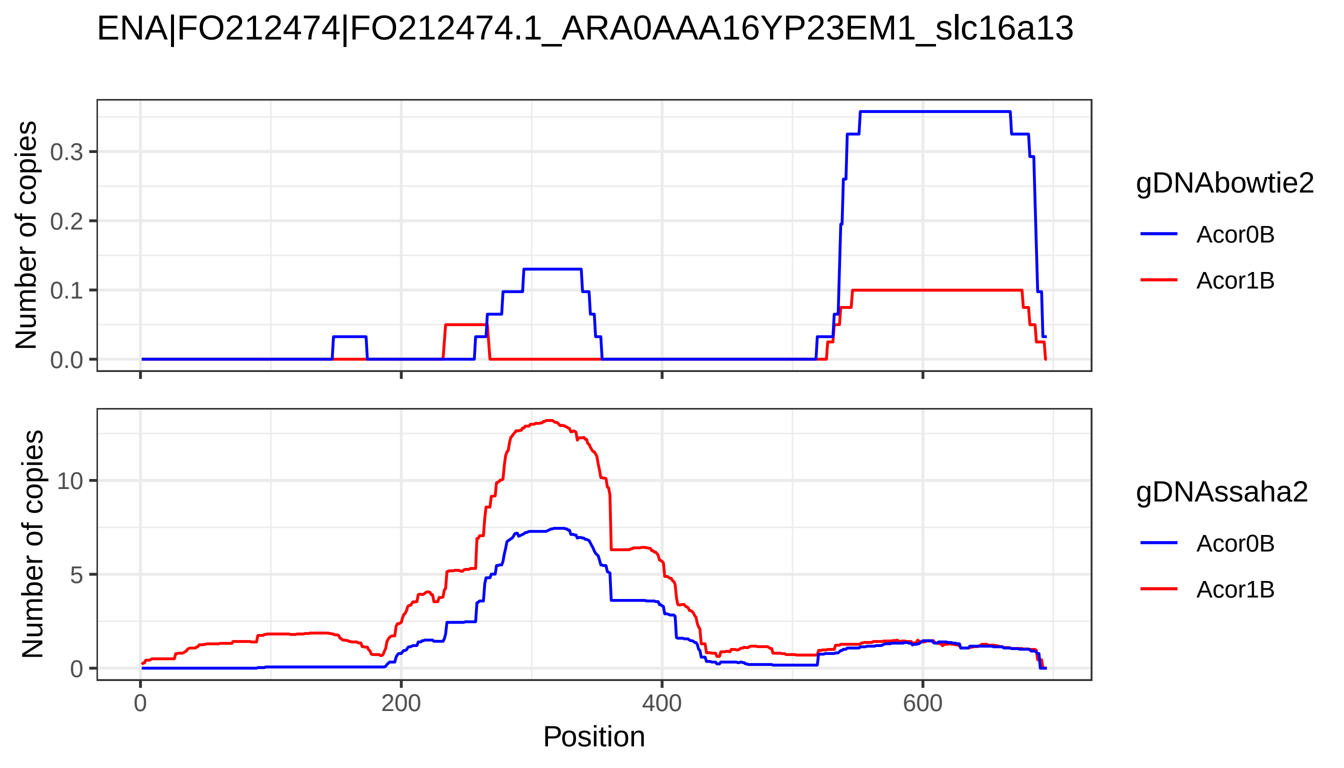

ENA|FO212474|FO212474.1_ARA0AAA16YP23EM1_slc16a13
Number of copies
0.0
0.1
0.2
0.3
Number of copies
0
5
10
0
200
400
600
Position
gDNAbowtie2
Acor0B
Acor1B
gDNAssaha2
Acor0B
Acor1B

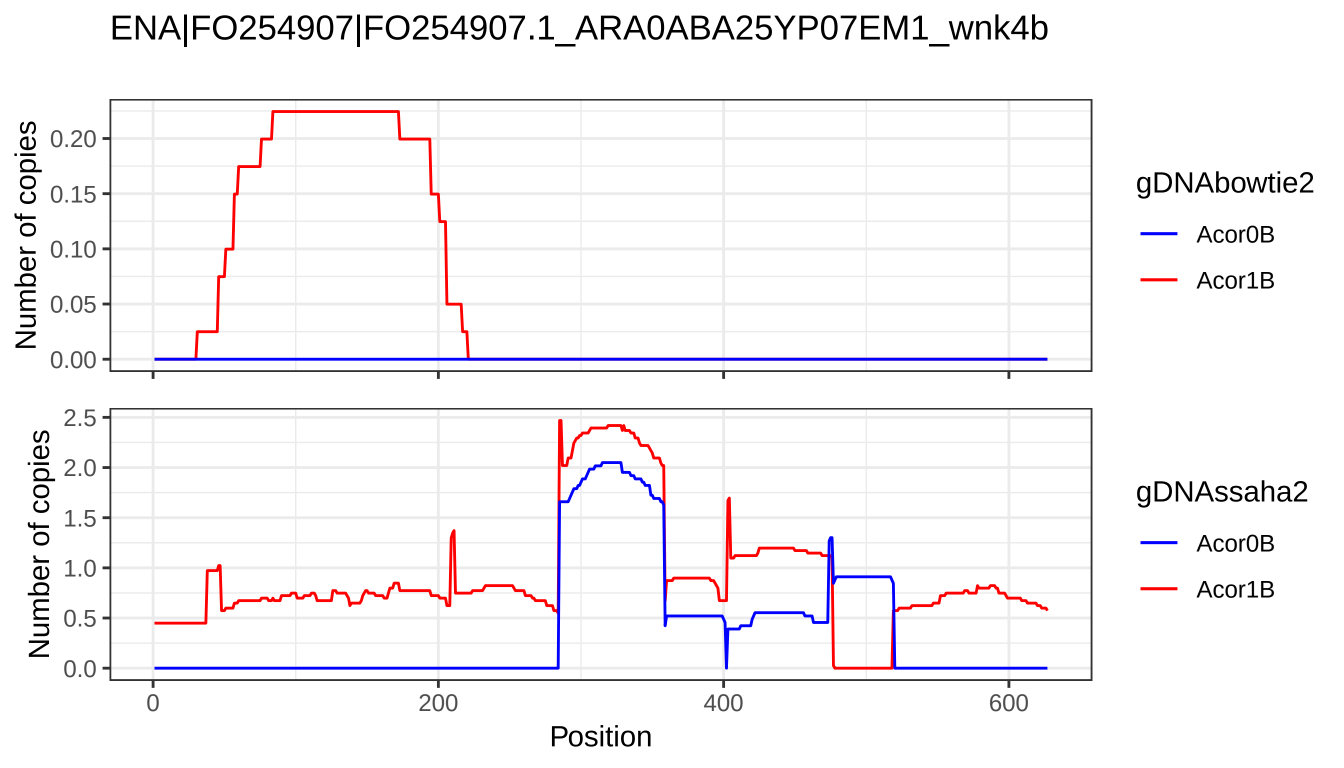
ENA|FO254907|FO254907.1_ARA0ABA25YP07EM1_wnk4b
Number of copies
0.20
0.15
0.10
0.05
0.00
gDNAbowtie2
Acor0B
Acor1B
Number of copies
2.5
2.0
1.5
1.0
0.5
0.0
gDNAssaha2
Acor0B
Acor1B
0
200
400
600
Position

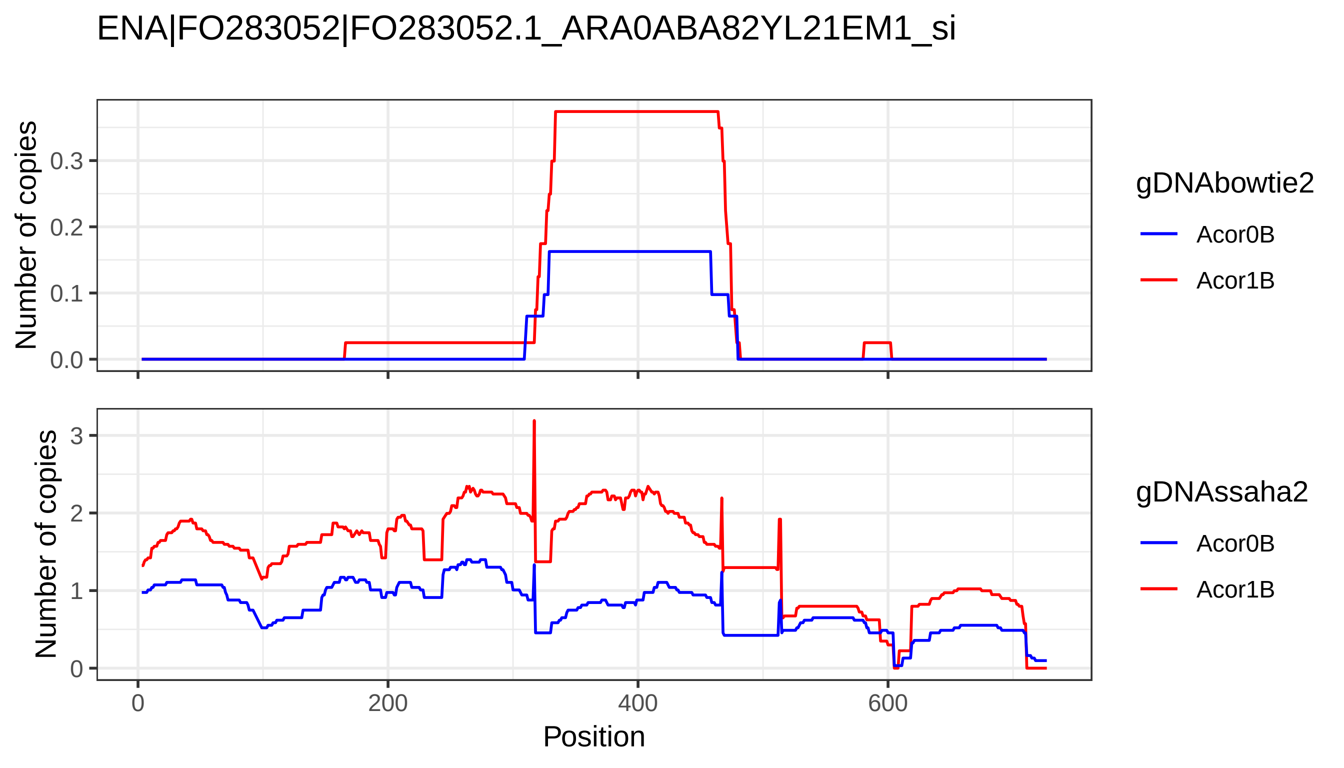

ENA|FO283052|FO283052.1_ARA0ABA82YL21EM1_si
Number of copies
0.0
0.1
0.2
0.3
gDNAbowtie2
Acor0B
Acor1B
Number of copies
0
1
2
3
gDNAssaha2
Acor0B
Acor1B
0
200
400
600
Position

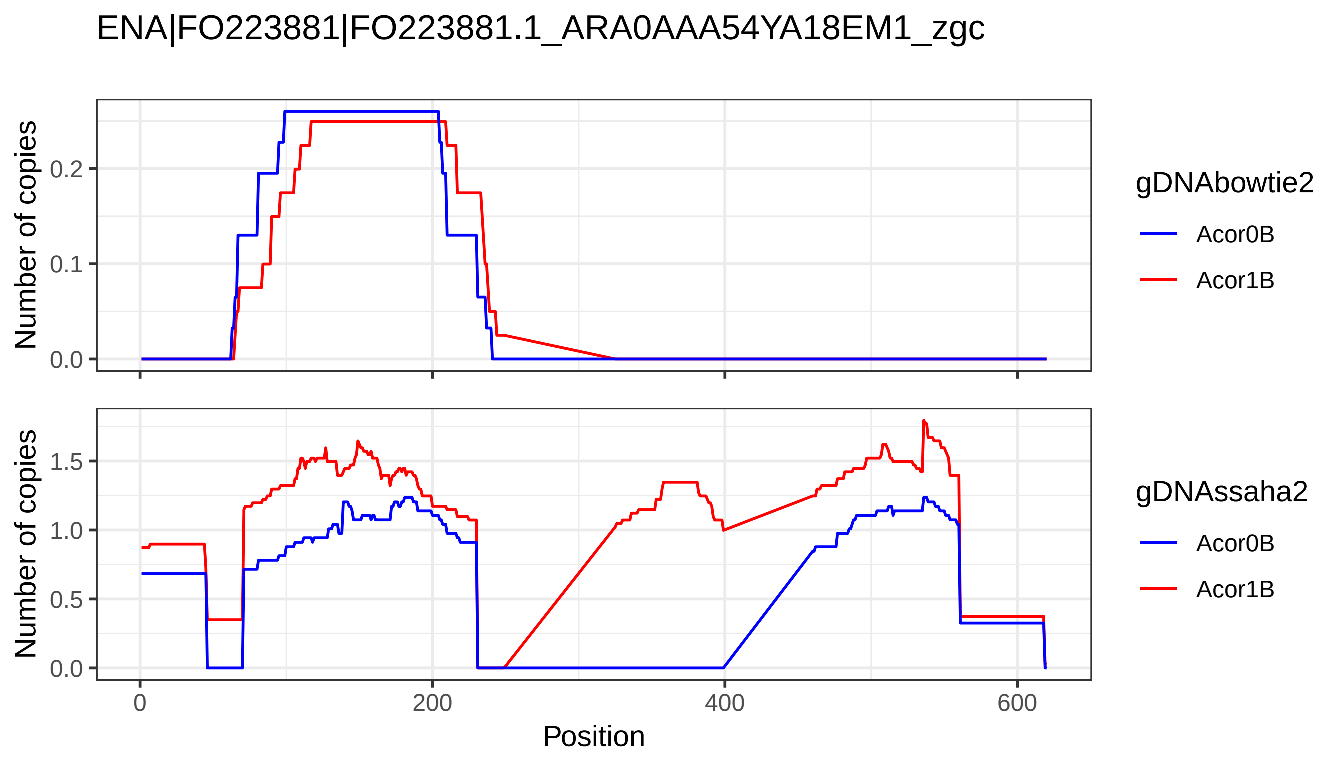

ENA|FO223881|FO223881.1_ARA0AAA54YA18EM1_zgc
Number of copies
0.0
0.1
0.2
gDNAbowtie2
Acor0B
Acor1B
Number of copies
0.0
0.5
1.0
1.5
gDNAssaha2
Acor0B
Acor1B
0
200
400
600
Position

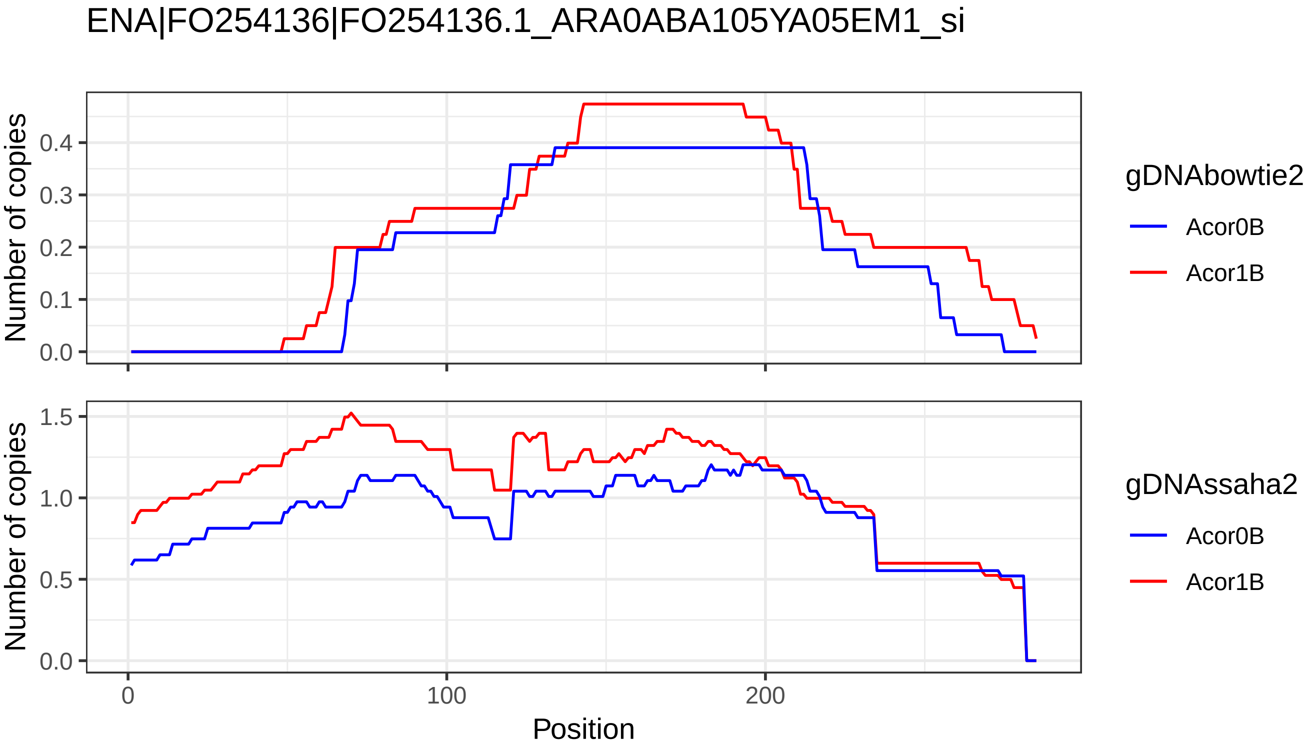
ENA|FO254136|FO254136.1_ARA0ABA105YA05EM1_si
Number of copies
0.0
0.1
0.2
0.3
0.4
0.5
1.0
1.5
0
100
200
Position
gDNAbowtie2
Acor0B
Acor1B
gDNAssaha2
Acor0B
Acor1B

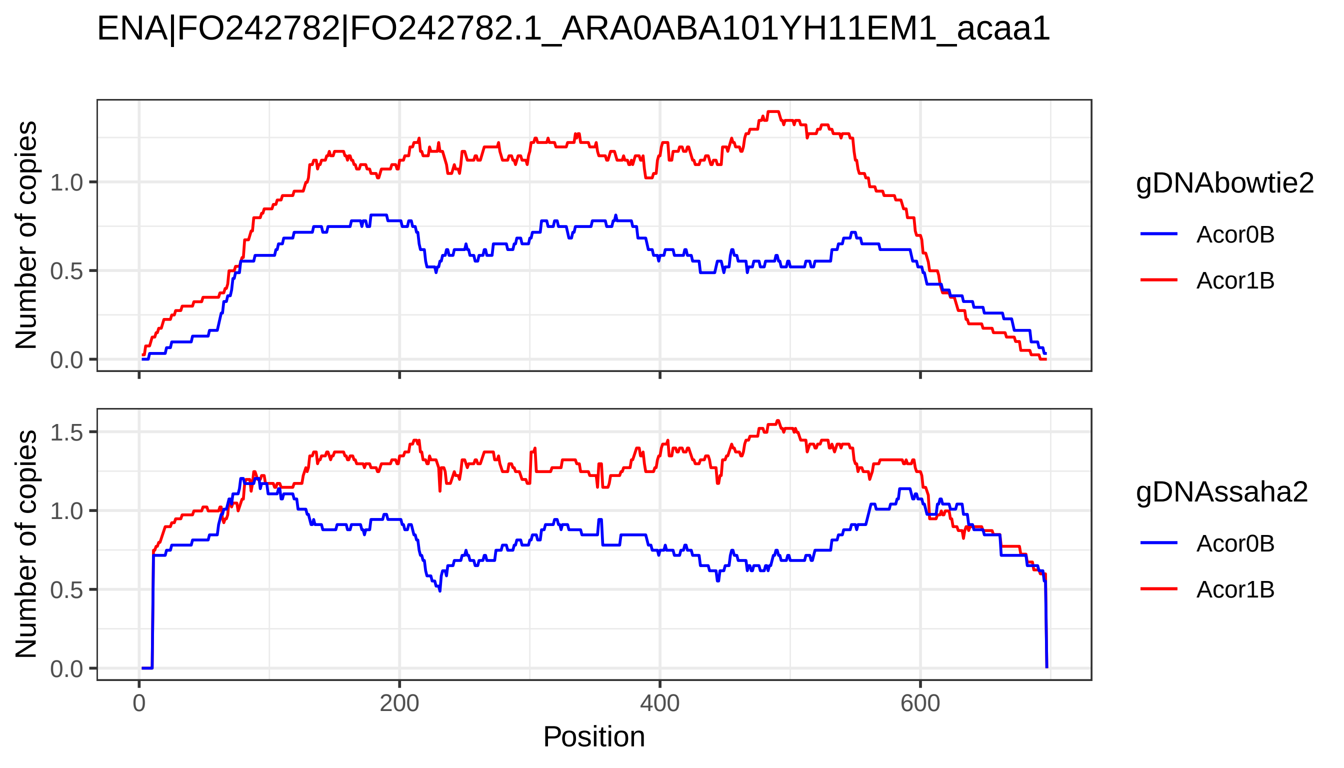

ENA|FO242782|FO242782.1_ARA0ABA101YH11EM1_acaa1
Number of copies
Number of copies
1.0
0.5
0.0
1.5
1.0
0.5
0.0
0
200
400
600
Position
gDNAbowtie2
Acor0B
Acor1B
gDNAssaha2
Acor0B
Acor1B

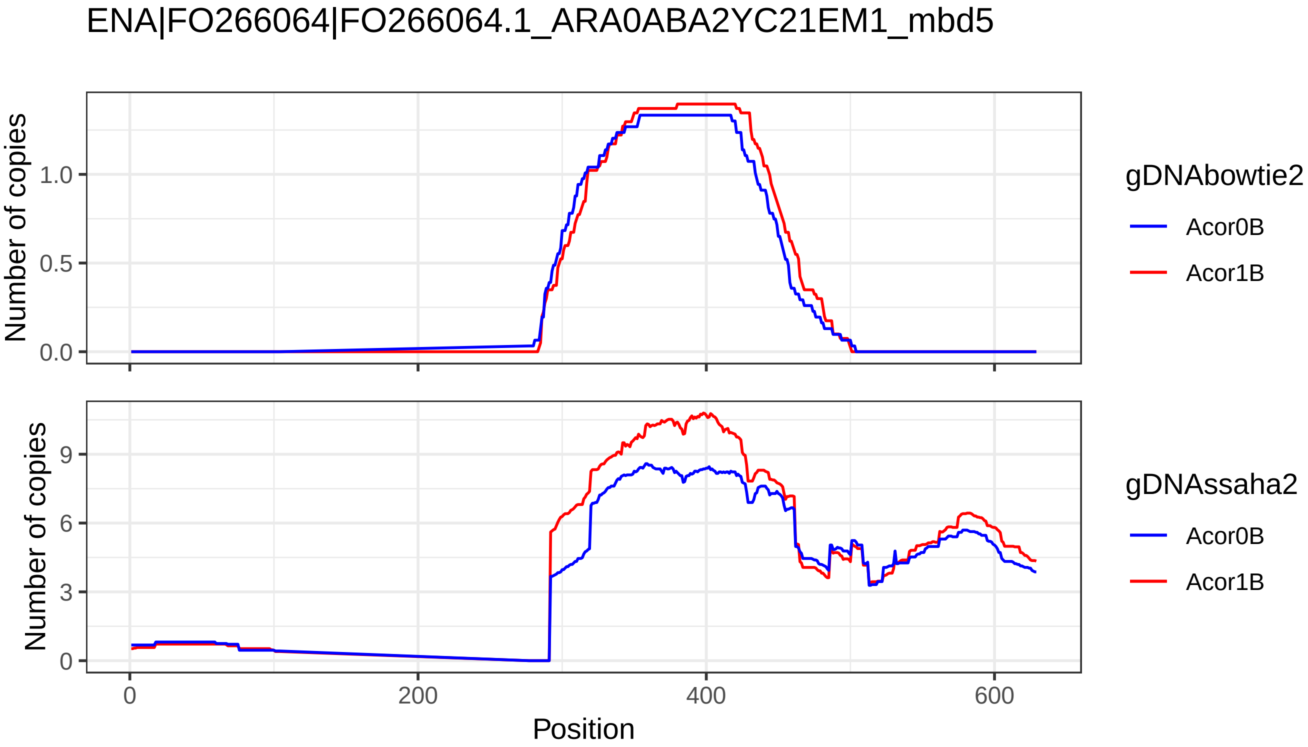

ENA|FO266064|FO266064.1_ARA0ABA2YC21EM1_mbd5
Number of copies
1.0
0.5
0.0
gDNAbowtie2
Acor0B
Acor1B
Number of copies
9
6
3
0
gDNAssaha2
Acor0B
Acor1B
0
200
400
600
Position

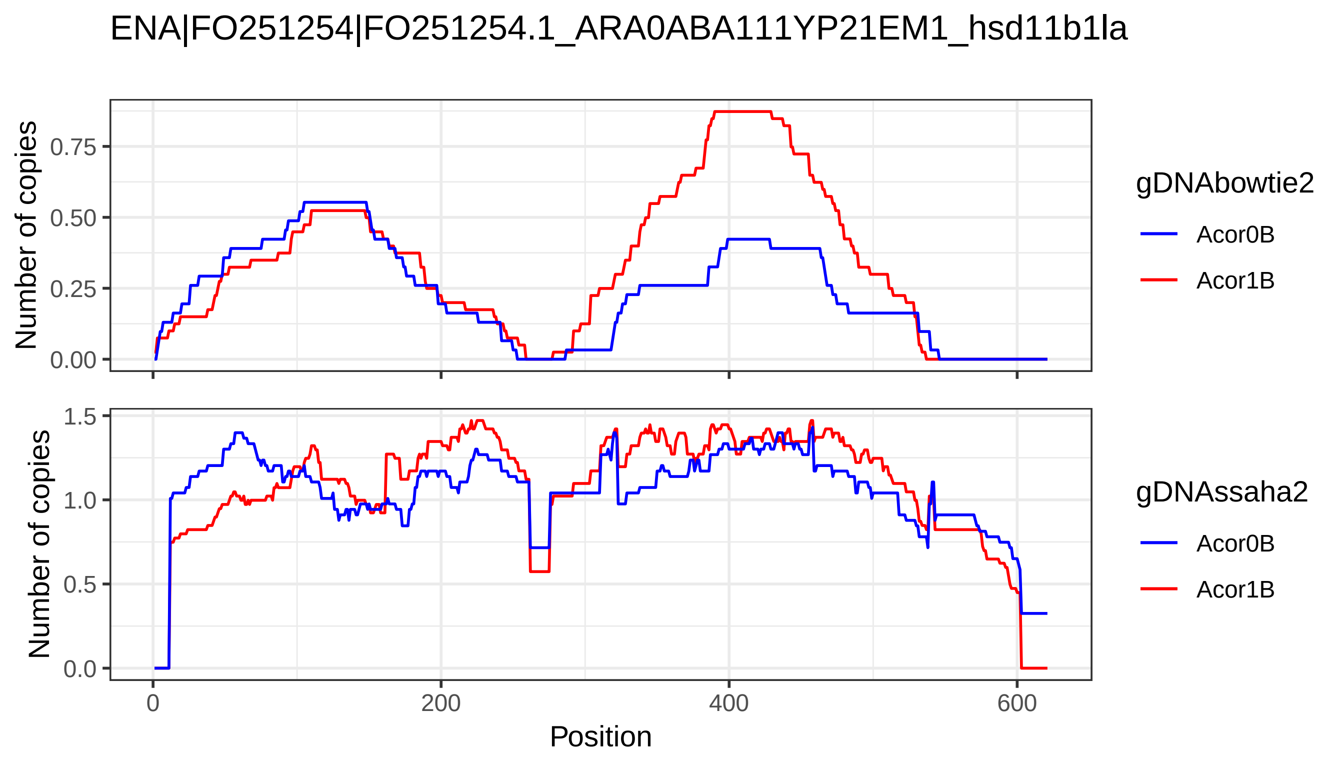
ENA|FO251254|FO251254.1_ARA0ABA111YP21EM1_hsd11b1la
Number of copies
0.00
0.25
0.50
0.75
Number of copies
0.0
0.5
1.0
1.5
0
200
400
600
Position
gDNAbowtie2
Acor0B
Acor1B
gDNAssaha2
Acor0B
Acor1B

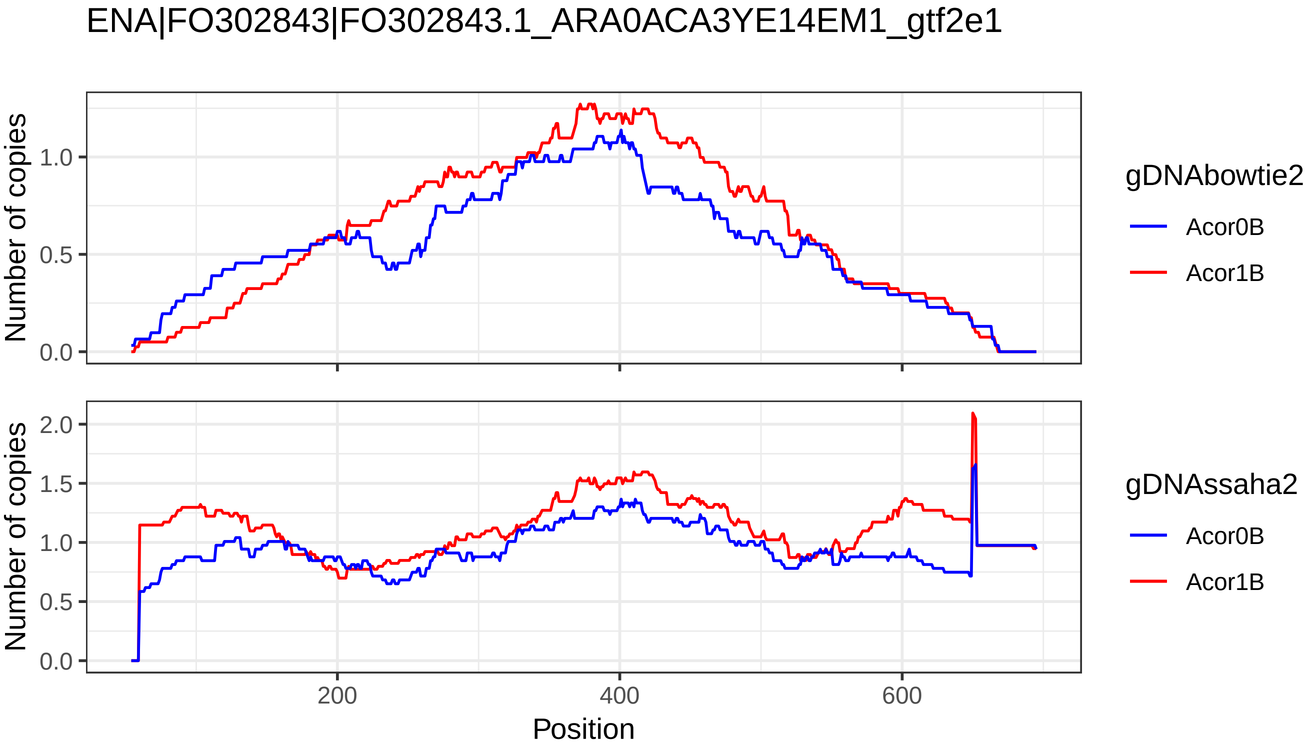
ENA|FO302843|FO302843.1_ARA0ACA3YE14EM1_gtf2e1
Number of copies
1.0
0.5
0.0
gDNAbowtie2
Acor0B
Acor1B
Number of copies
2.0
1.5
1.0
0.5
0.0
gDNAssaha2
Acor0B
Acor1B
200
400
600
Position

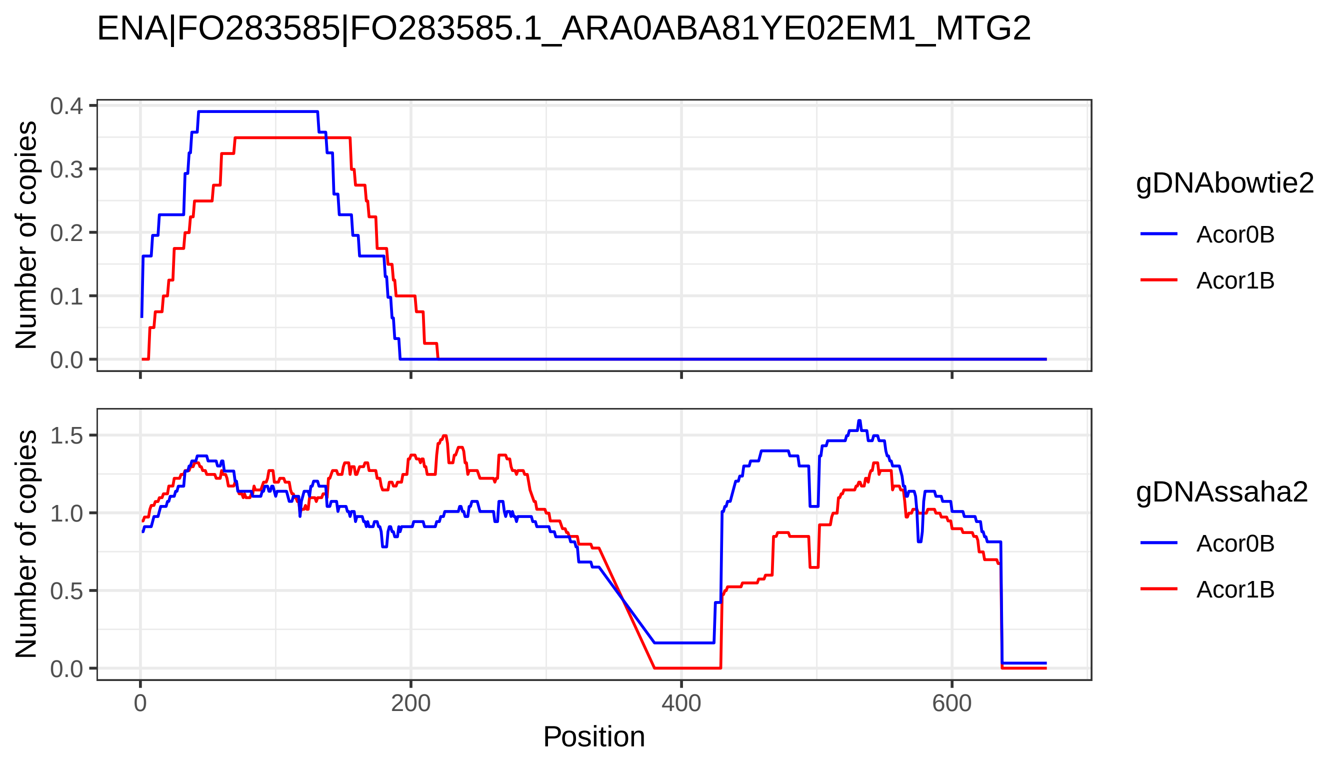
ENA|FO283585|FO283585.1_ARA0ABA81YE02EM1_MTG2
Number of copies
0.0
0.1
0.2
0.3
0.4
gDNAbowtie2
Acor0B
Acor1B
Number of copies
0.0
0.5
1.0
1.5
gDNAssaha2
Acor0B
Acor1B
0
200
400
600
Position

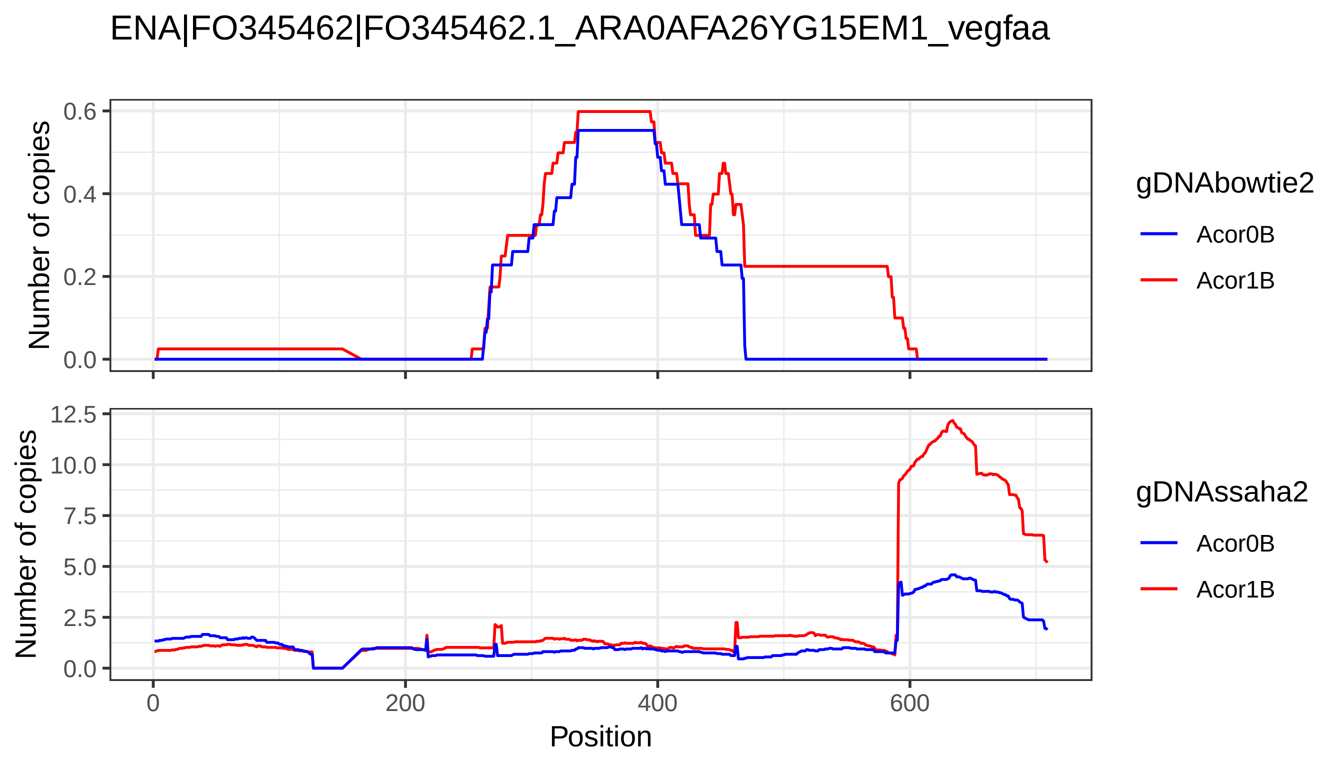

ENA|FO345462|FO345462.1_ARA0AFA26YG15EM1_vegfaa
Number of copies
0.0
0.2
0.4
0.6
gDNAbowtie2
Acor0B
Acor1B
Number of copies
0.0
2.5
5.0
7.5
10.0
12.5
gDNAssaha2
Acor0B
Acor1B
0
200
400
600
Position

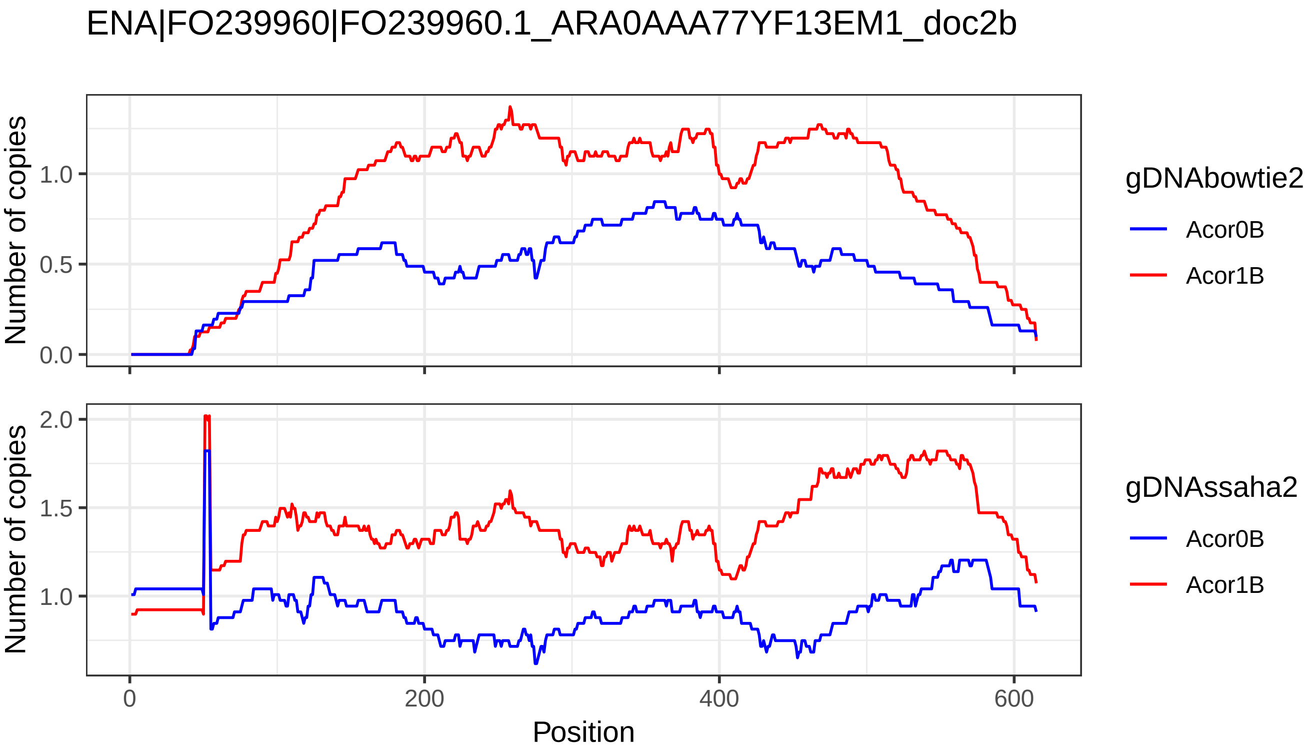
ENA|FO239960|FO239960.1_ARA0AAA77YF13EM1_doc2b
Number of copies
1.0
0.5
0.0
gDNAbowtie2
Acor0B
Acor1B
Number of copies
2.0
1.5
1.0
gDNAssaha2
Acor0B
Acor1B
0
200
400
600
Position

ENA|FO222595|FO222595.1_ARA0AAA57YJ09EM1_si

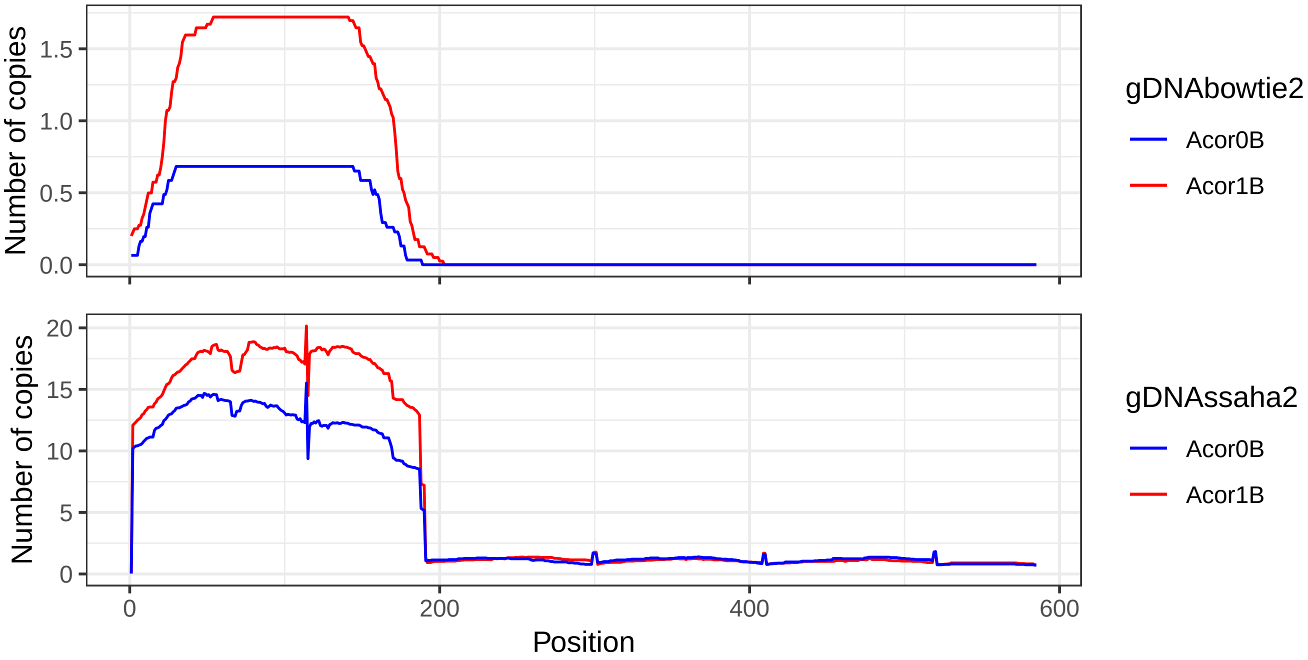

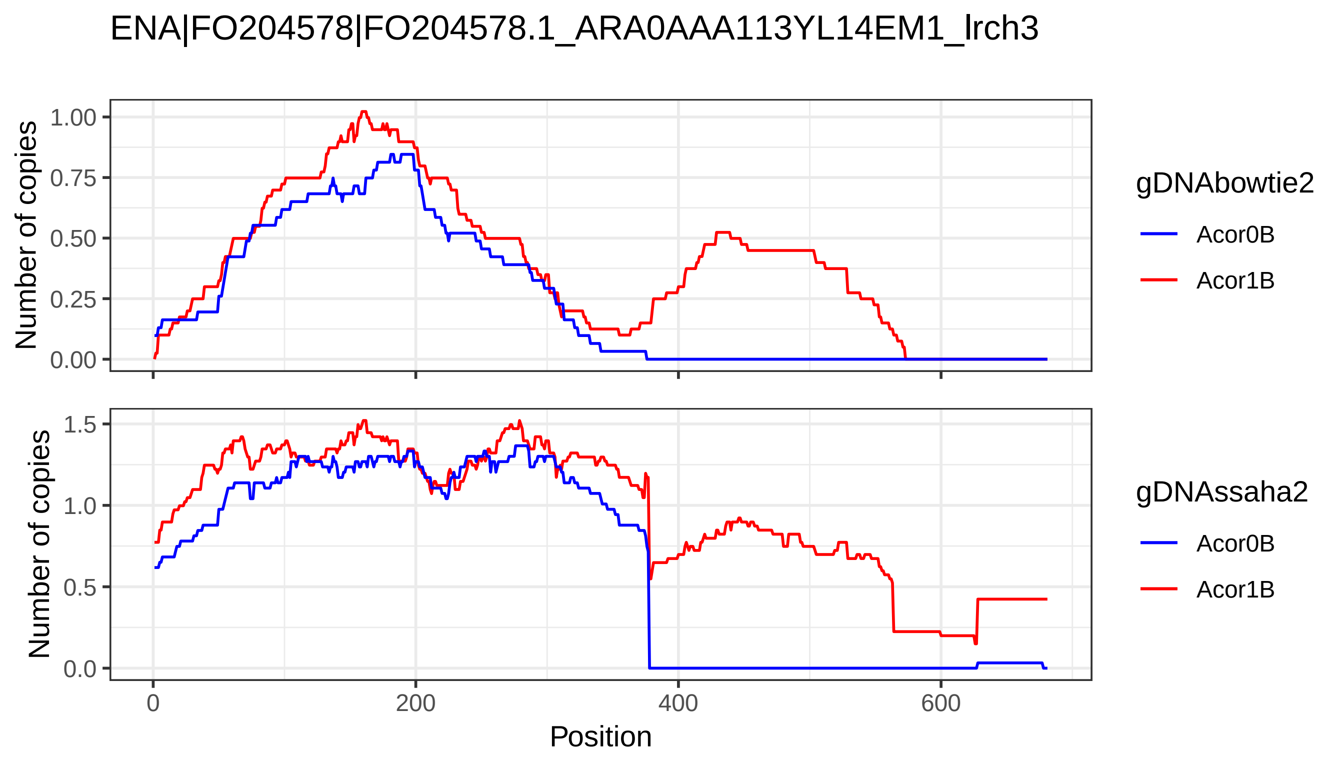

ENA|FO204578|FO204578.1_ARA0AAA113YL14EM1_lrch3
Number of copies
1.00
0.75
0.50
0.25
0.00
gDNAbowtie2
Acor0B
Acor1B
Number of copies
1.5
1.0
0.5
0.0
gDNAssaha2
Acor0B
Acor1B
0
200
400
600
Position

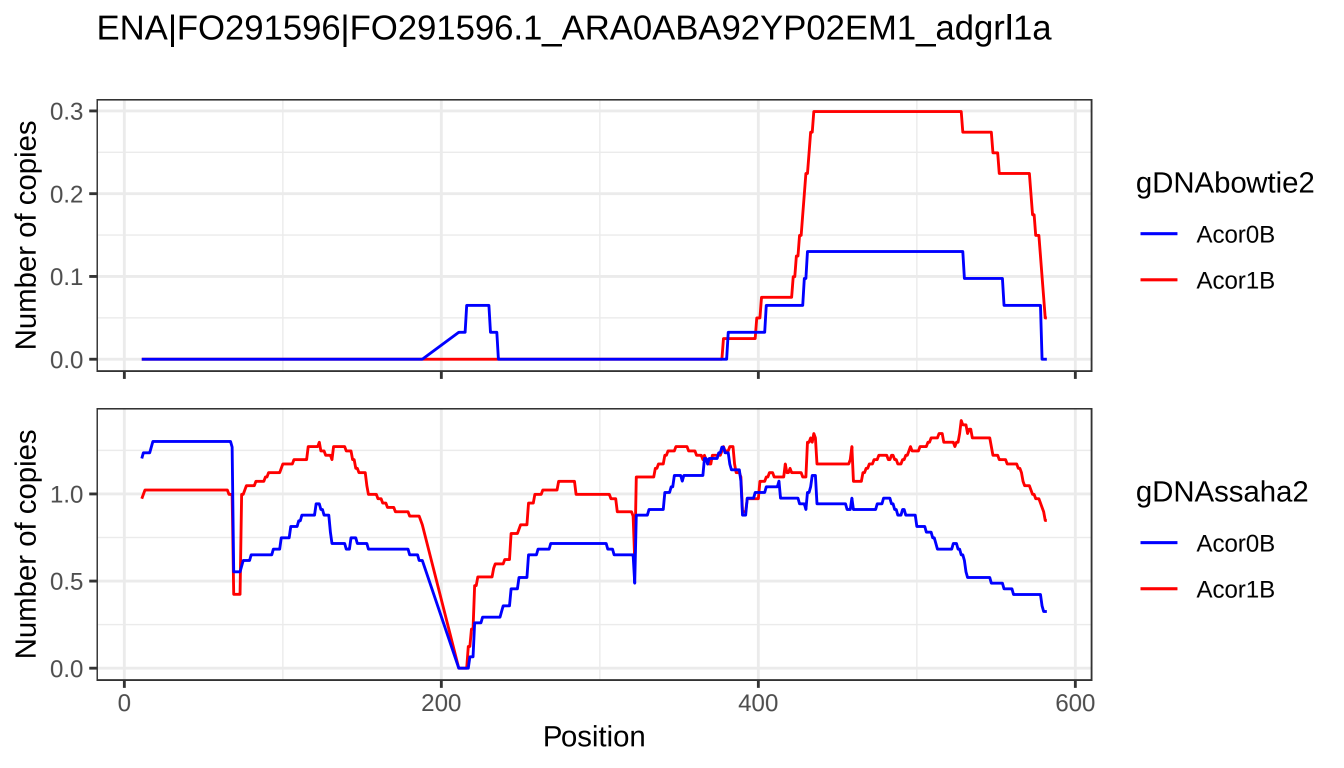

ENA|FO291596|FO291596.1_ARA0ABA92YP02EM1_adgrl1a
Number of copies
0.0
0.1
0.2
0.3
gDNAbowtie2
Acor0B
Acor1B
Number of copies
0.0
0.5
1.0
gDNAssaha2
Acor0B
Acor1B
0
200
400
600
Position

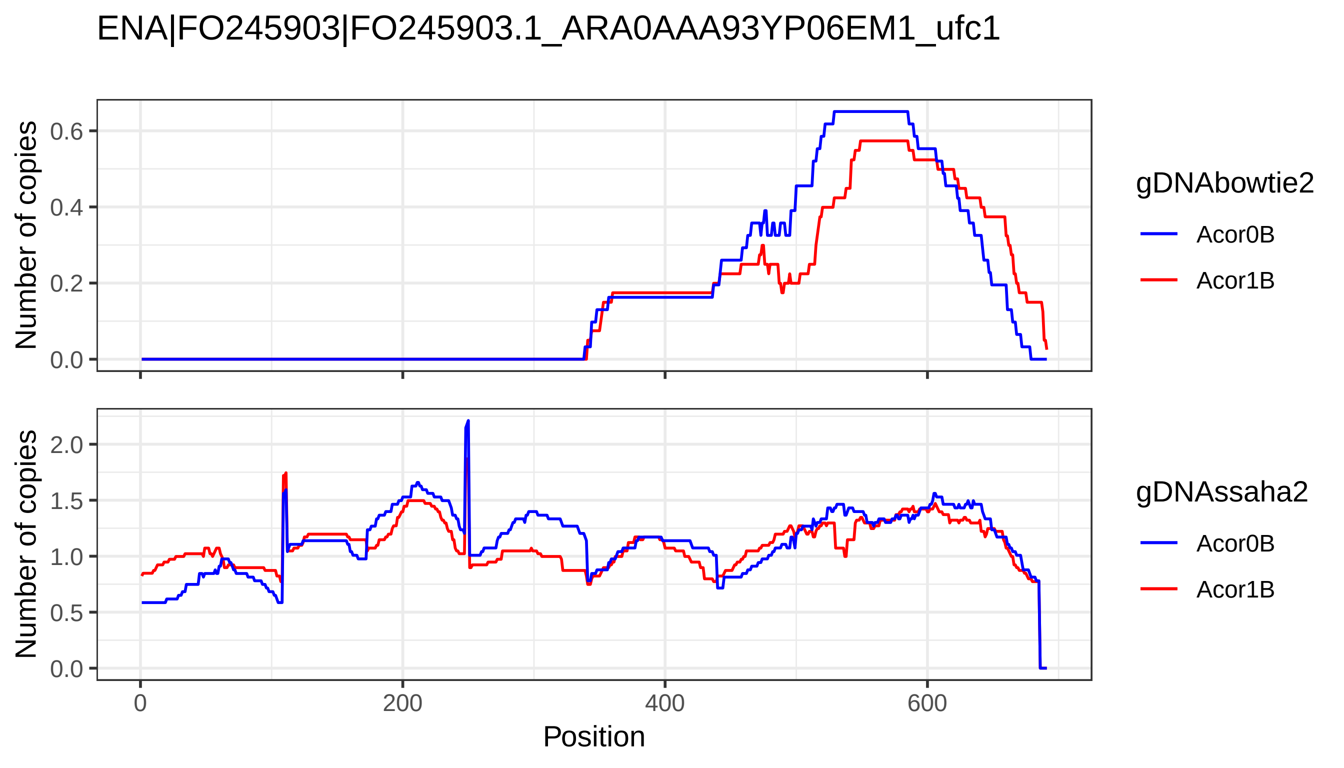

ENA|FO245903|FO245903.1_ARA0AAA93YP06EM1_ufc1
Number of copies
0.0
0.2
0.4
0.6
gDNAbowtie2
Acor0B
Acor1B
Number of copies
0.0
0.5
1.0
1.5
2.0
gDNAssaha2
Acor0B
Acor1B
0
200
400
600
Position

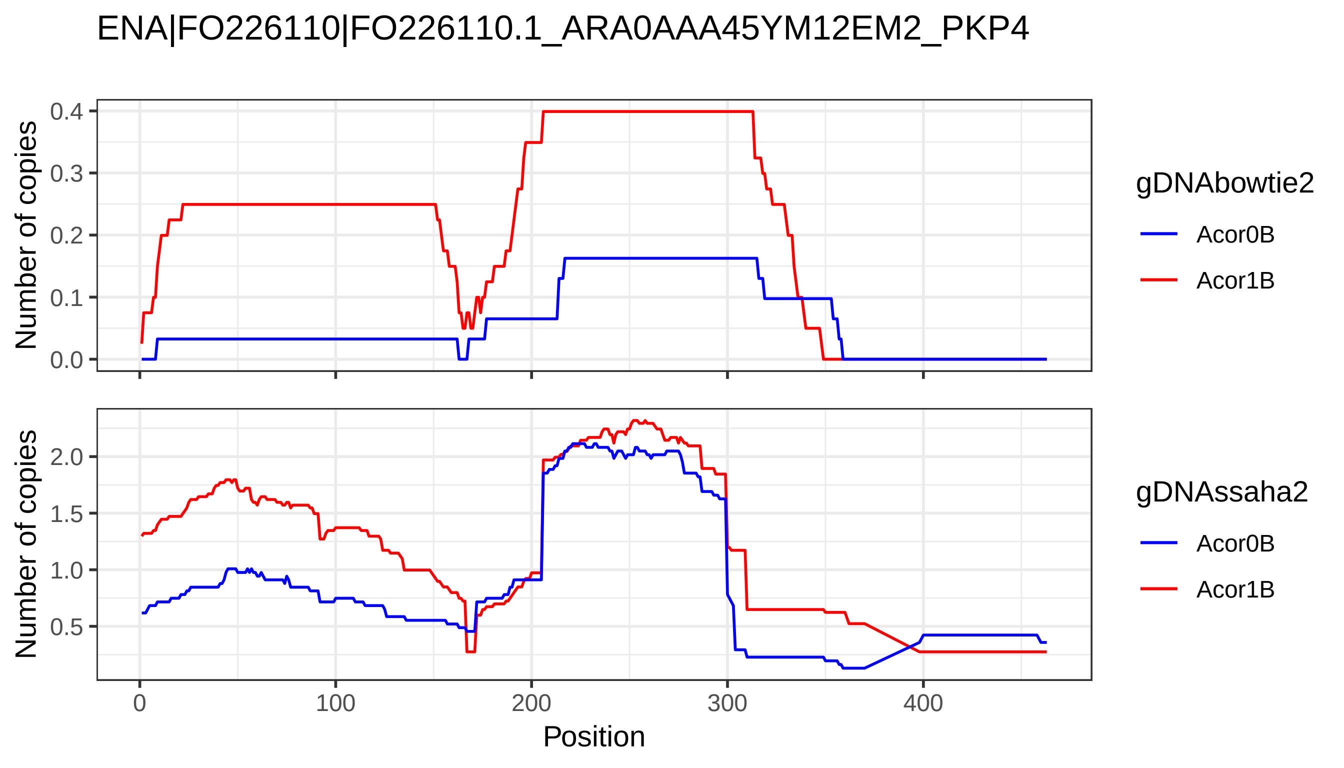

ENA|FO226110|FO226110.1_ARA0AAA45YM12EM2_PKP4
Number of copies
0.4
0.3
0.2
0.1
0.0
gDNAbowtie2
Acor0B
Acor1B
Number of copies
2.0
1.5
1.0
0.5
gDNAssaha2
Acor0B
Acor1B
0
100
200
300
400
Position

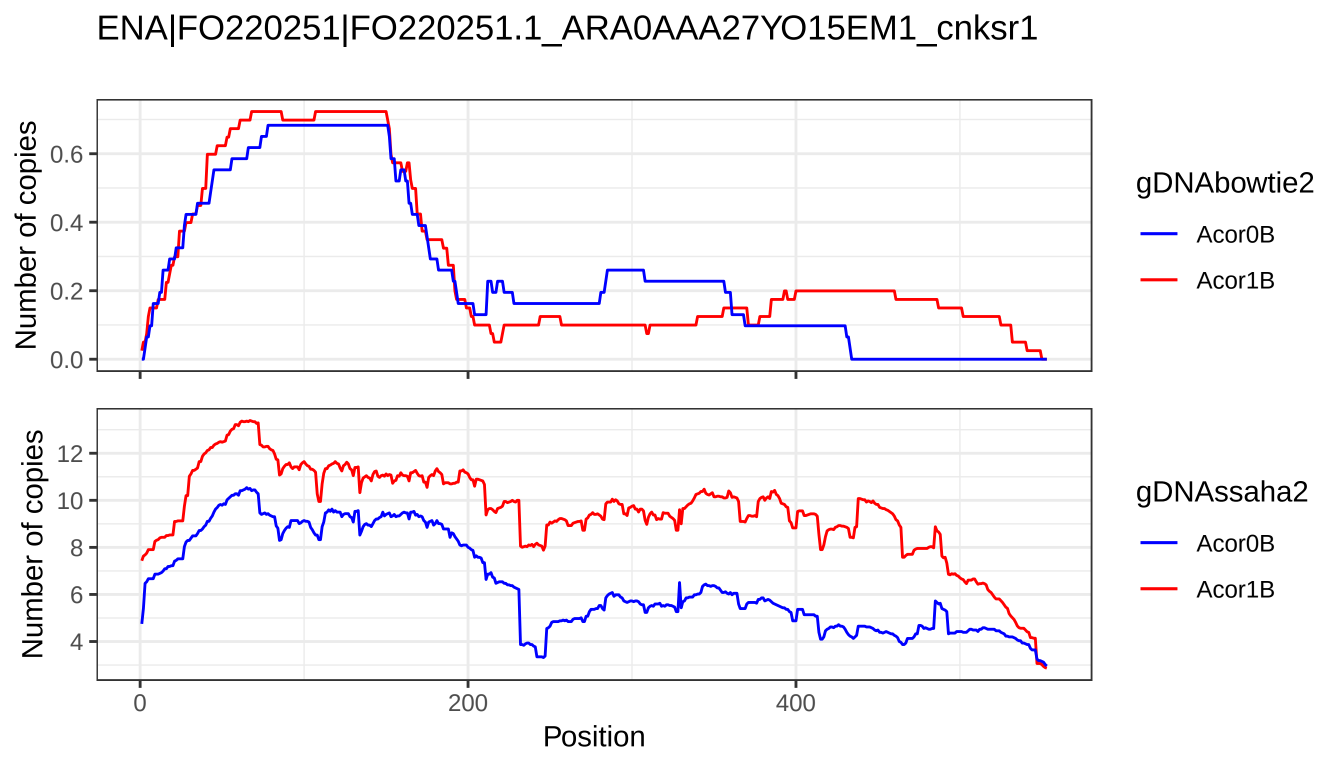
ENA|FO220251|FO220251.1_ARA0AAA27YO15EM1_cnksr1
Number of copies
0.0
0.2
0.4
0.6
Number of copies
4
6
8
10
12
0
200
400
Position
gDNAbowtie2
Acor0B
Acor1B
gDNAssaha2
Acor0B
Acor1B

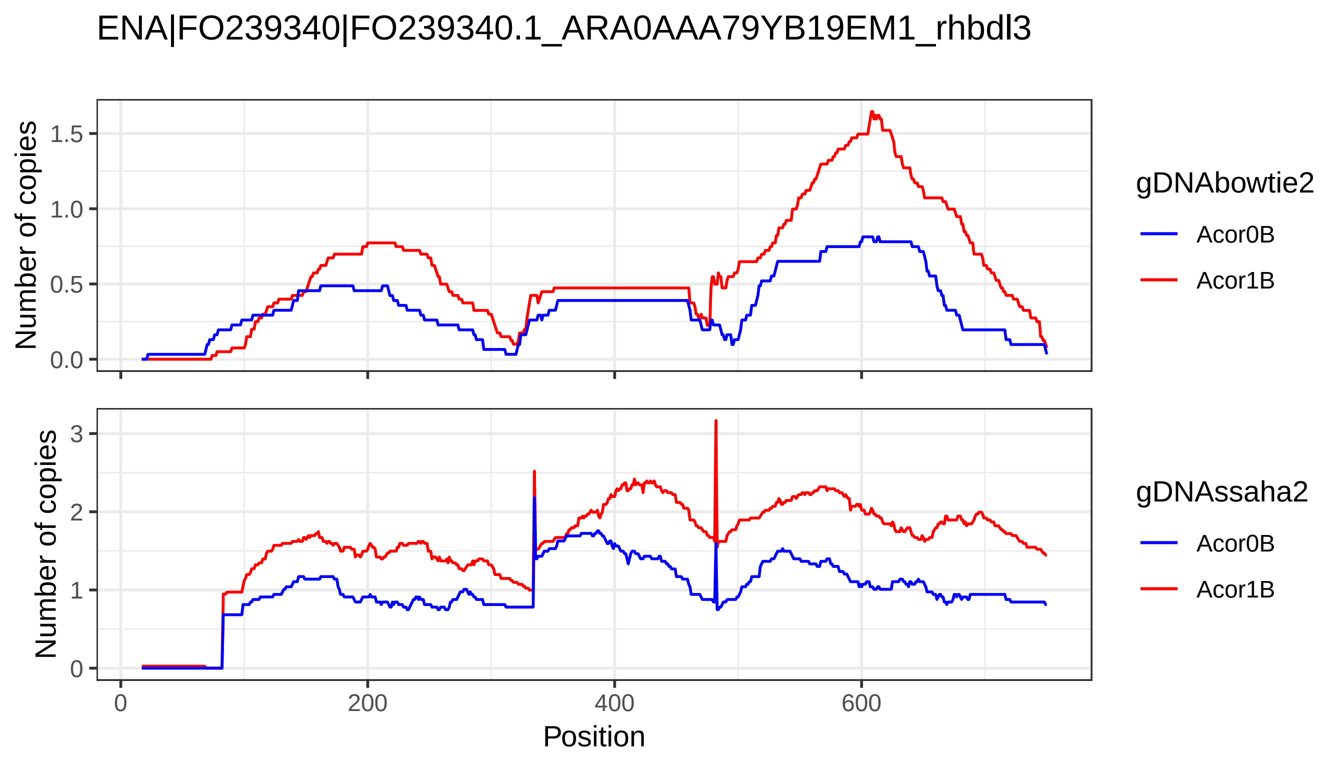
ENA|FO239340|FO239340.1_ARA0AAA79YB19EM1_rhbdl3
Number of copies
1.5
1.0
0.5
0.0
gDNAbowtie2
Acor0B
Acor1B
Number of copies
3
2
1
0
gDNAssaha2
Acor0B
Acor1B
0
200
400
600
Position

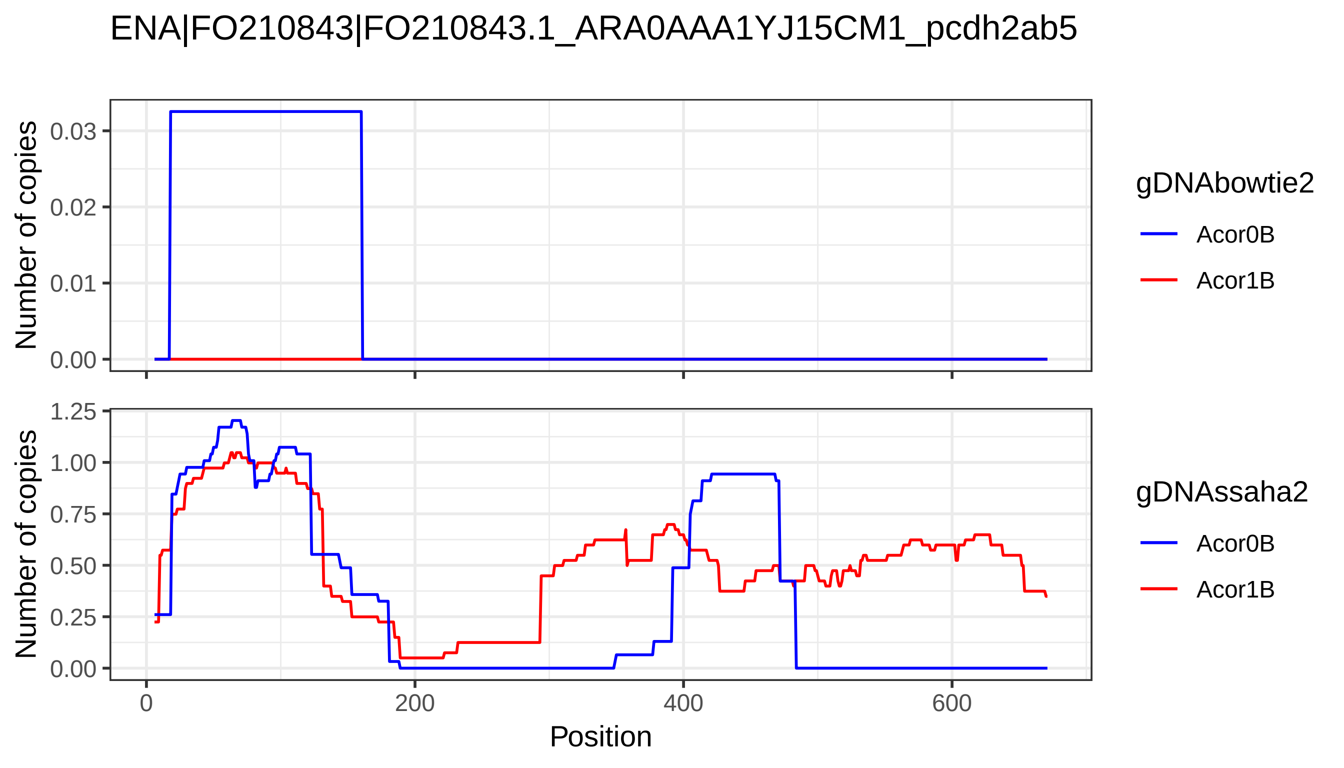

ENA|FO210843|FO210843.1_ARA0AAA1YJ15CM1_pcdh2ab5
Number of copies
0.03
0.02
0.01
0.00
gDNAbowtie2
Acor0B
Acor1B
Number of copies
1.25
1.00
0.75
0.50
0.25
0.00
gDNAssaha2
Acor0B
Acor1B
0
200
400
600
Position

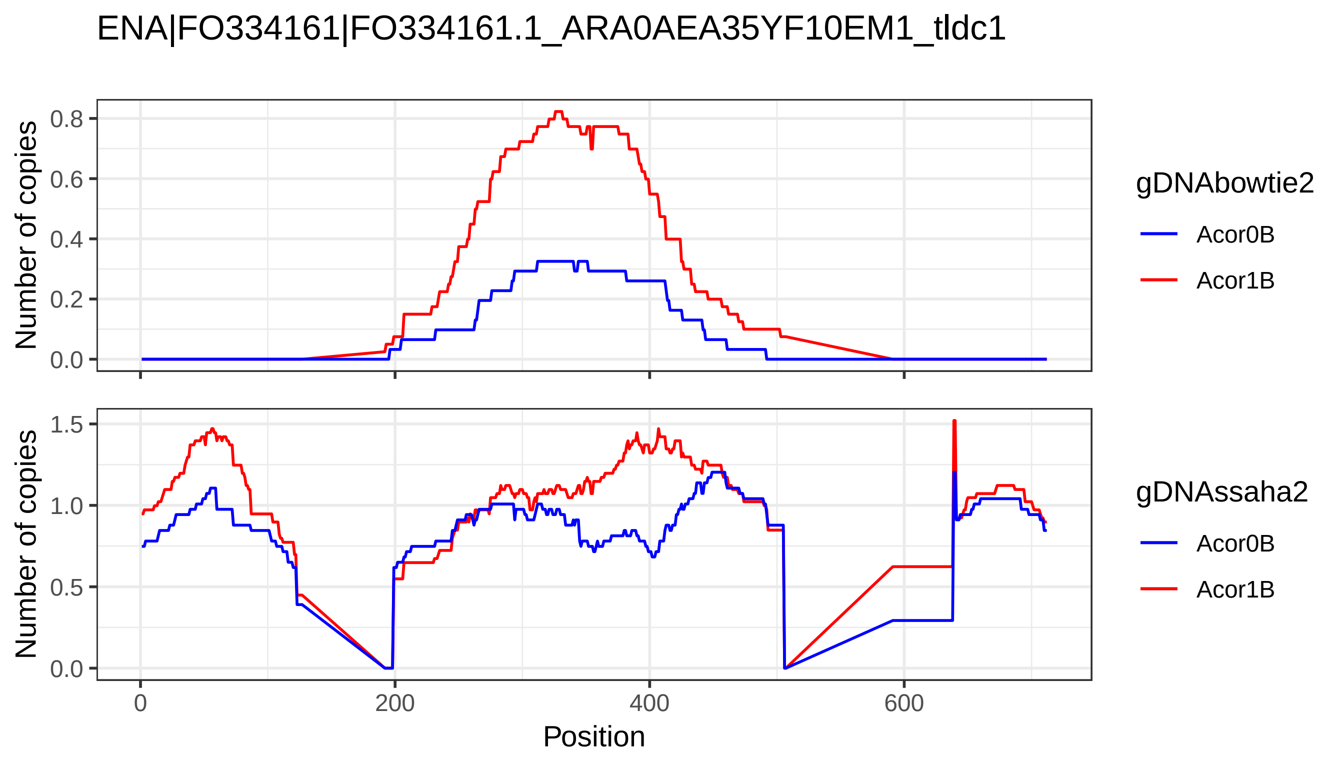

ENA|FO334161|FO334161.1_ARA0AEA35YF10EM1_tldc1
Number of copies
0.0
0.2
0.4
0.6
0.8
gDNAbowtie2
Acor0B
Acor1B
Number of copies
0.0
0.5
1.0
1.5
gDNAssaha2
Acor0B
Acor1B
0
200
400
600
Position

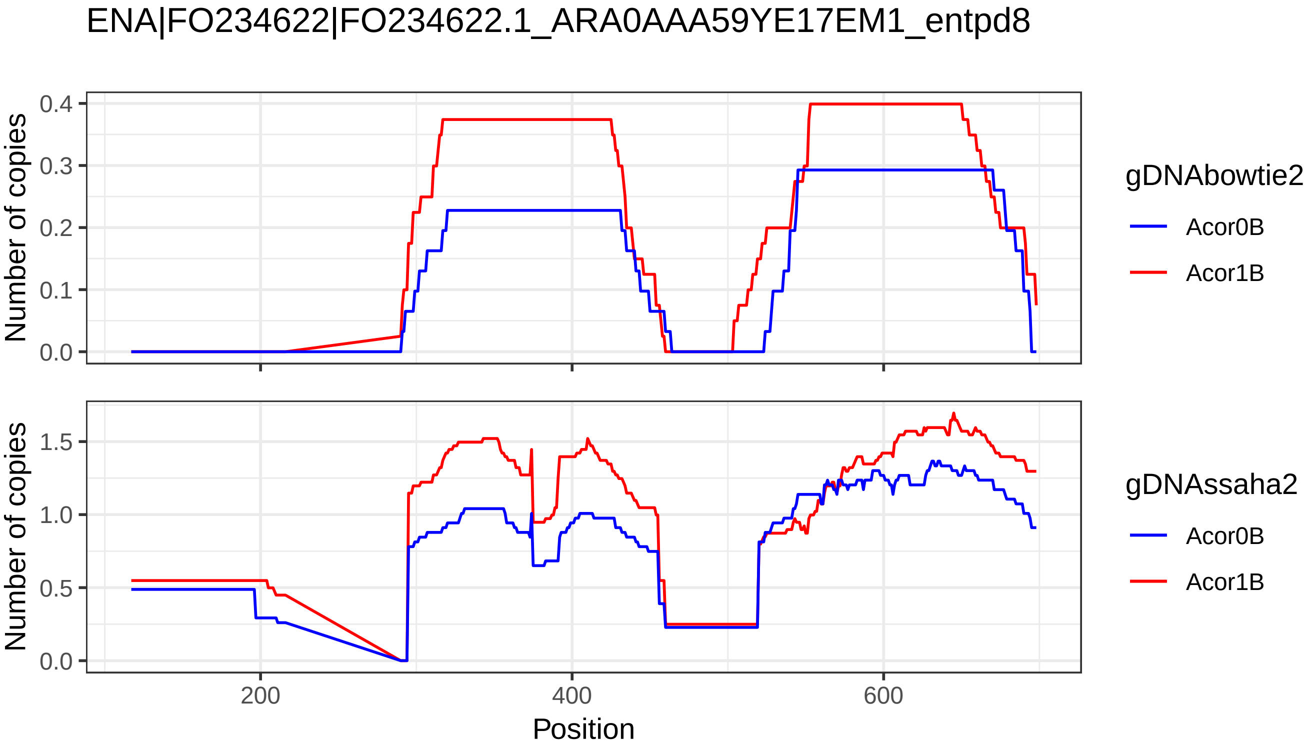
ENA|FO234622|FO234622.1_ARA0AAA59YE17EM1_entpd8
Number of copies
0.0
0.1
0.2
0.3
0.4
0.0
0.5
1.0
1.5
200
400
600
Position
gDNAbowtie2
Acor0B
Acor1B
gDNAssaha2
Acor0B
Acor1B

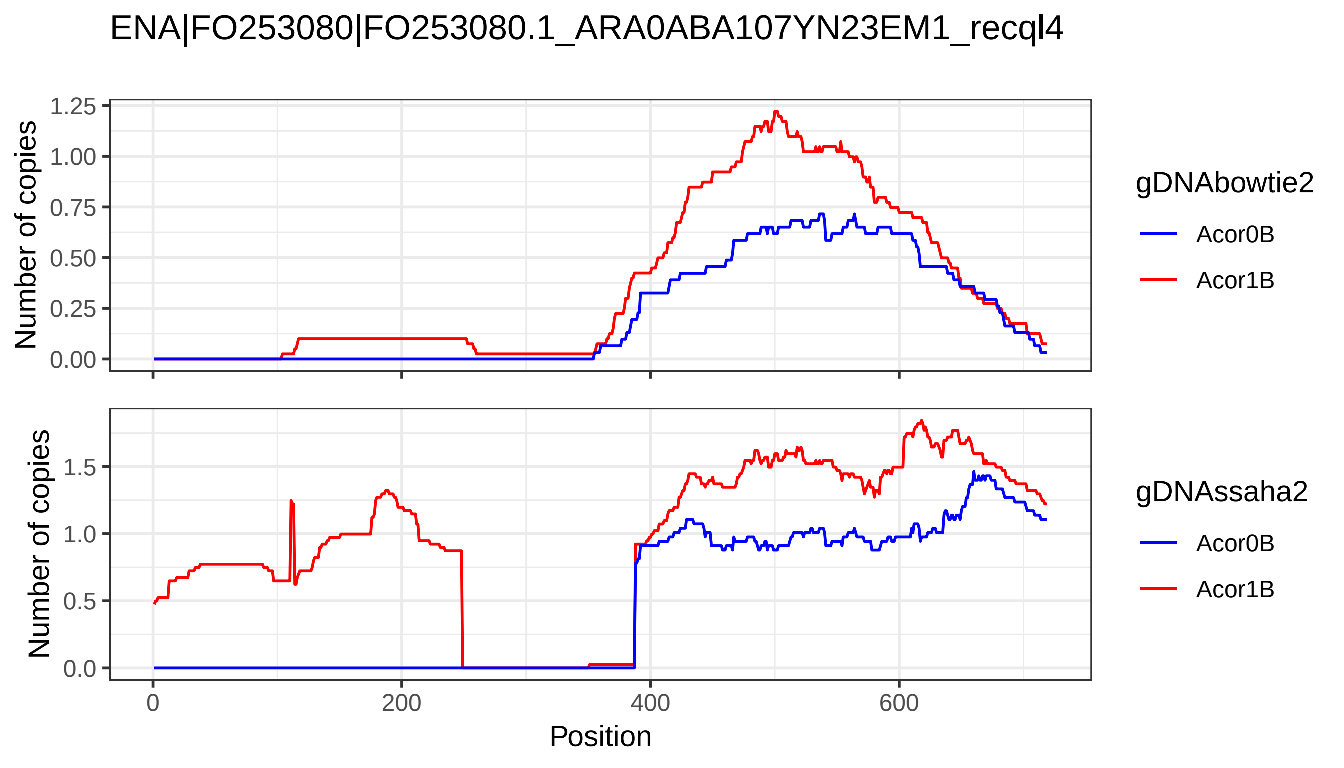
ENA|FO253080|FO253080.1_ARA0ABA107YN23EM1_recql4
Number of copies
1.25
1.00
0.75
0.50
0.25
0.00
gDNAbowtie2
Acor0B
Acor1B
Number of copies
1.5
1.0
0.5
0.0
gDNAssaha2
Acor0B
Acor1B
0
200
400
600
Position

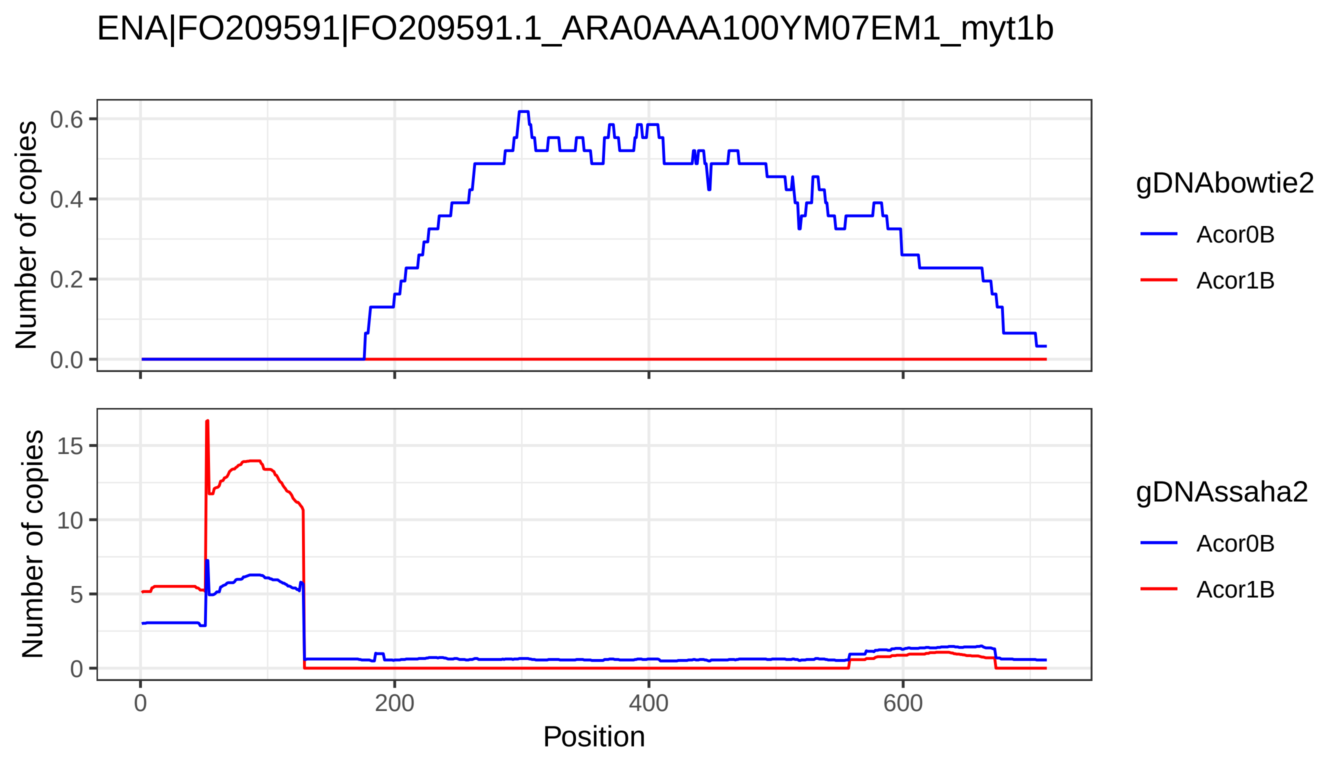

ENA|FO209591|FO209591.1_ARA0AAA100YM07EM1_myt1b
Number of copies
0.0
0.2
0.4
0.6
gDNAbowtie2
Acor0B
Acor1B
Number of copies
0
5
10
15
gDNAssaha2
Acor0B
Acor1B
0
200
400
600
Position

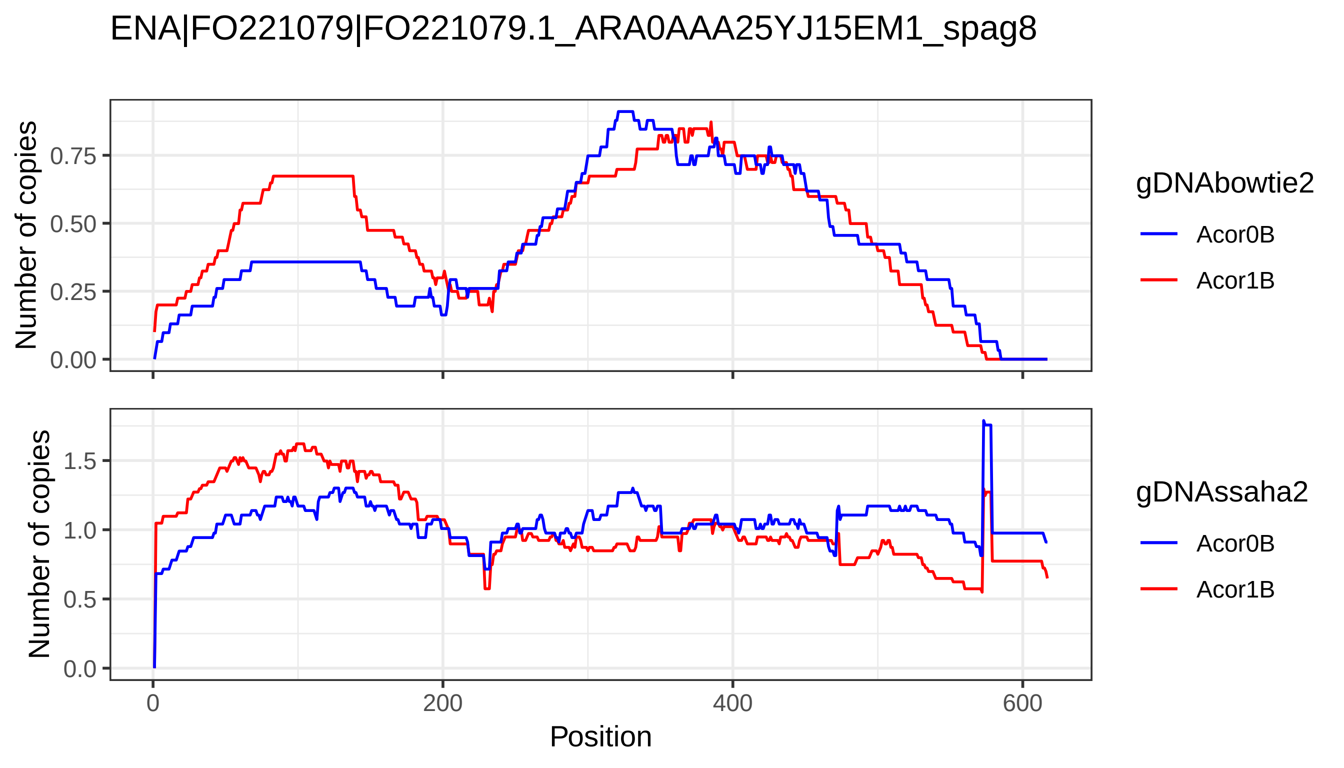

ENA|FO221079|FO221079.1_ARA0AAA25YJ15EM1_spag8
Number of copies
0.00
0.25
0.50
0.75
gDNAbowtie2
Acor0B
Acor1B
Number of copies
0.0
0.5
1.0
1.5
gDNAssaha2
Acor0B
Acor1B
0
200
400
600
Position

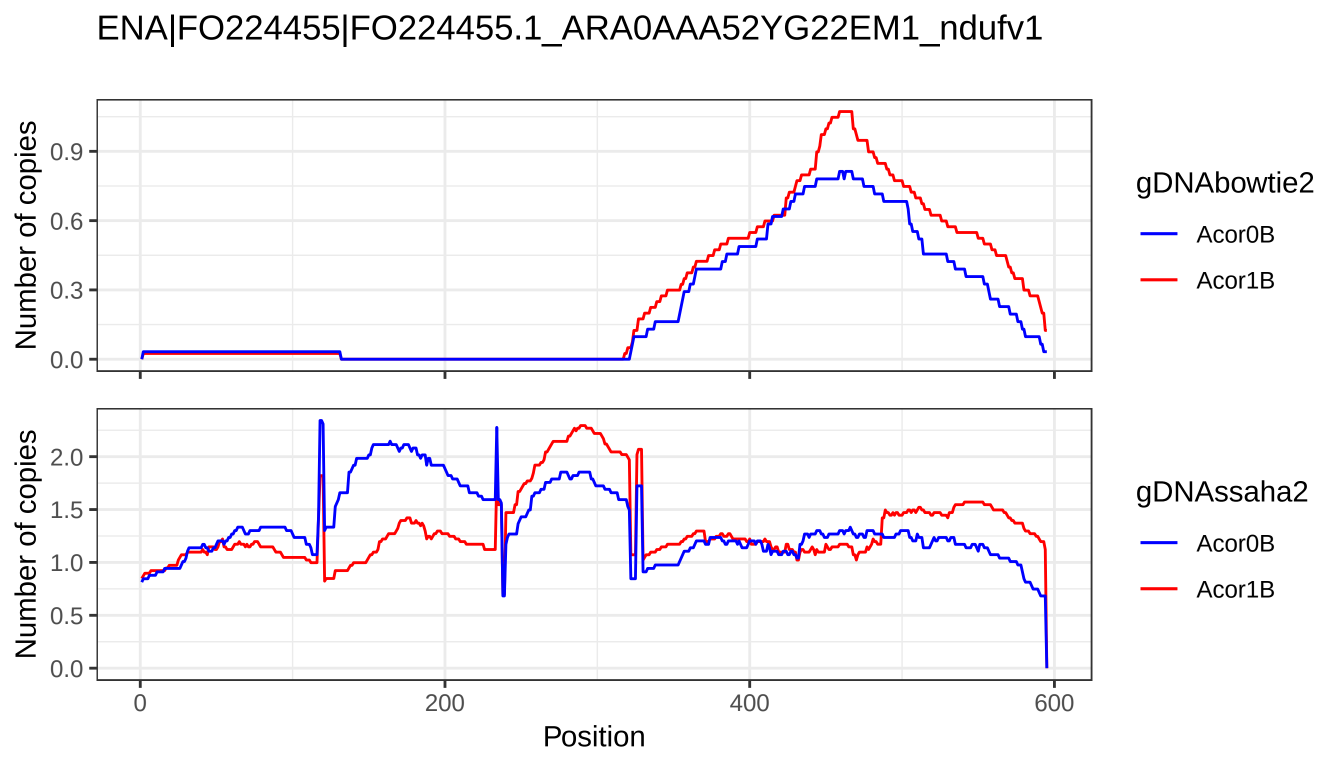

ENA|FO224455|FO224455.1_ARA0AAA52YG22EM1_ndufv1
Number of copies
0.0
0.3
0.6
0.9
gDNAbowtie2
Acor0B
Acor1B
Number of copies
0.0
0.5
1.0
1.5
2.0
gDNAssaha2
Acor0B
Acor1B
0
200
400
600
Position

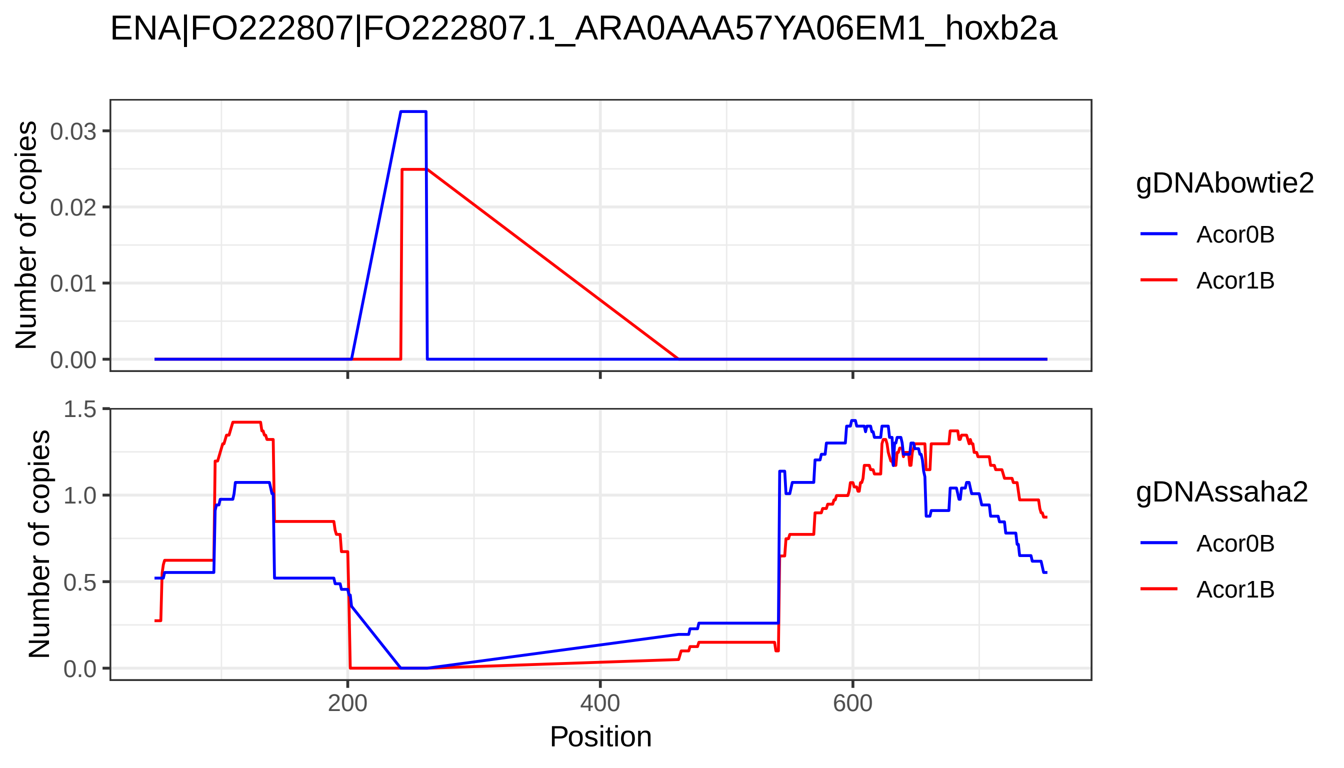
ENA|FO222807|FO222807.1_ARA0AAA57YA06EM1_hoxb2a
Number of copies
0.03
0.02
0.01
0.00
gDNAbowtie2
Acor0B
Acor1B
Number of copies
1.5
1.0
0.5
0.0
200
400
600
Position
gDNAssaha2
Acor0B
Acor1B

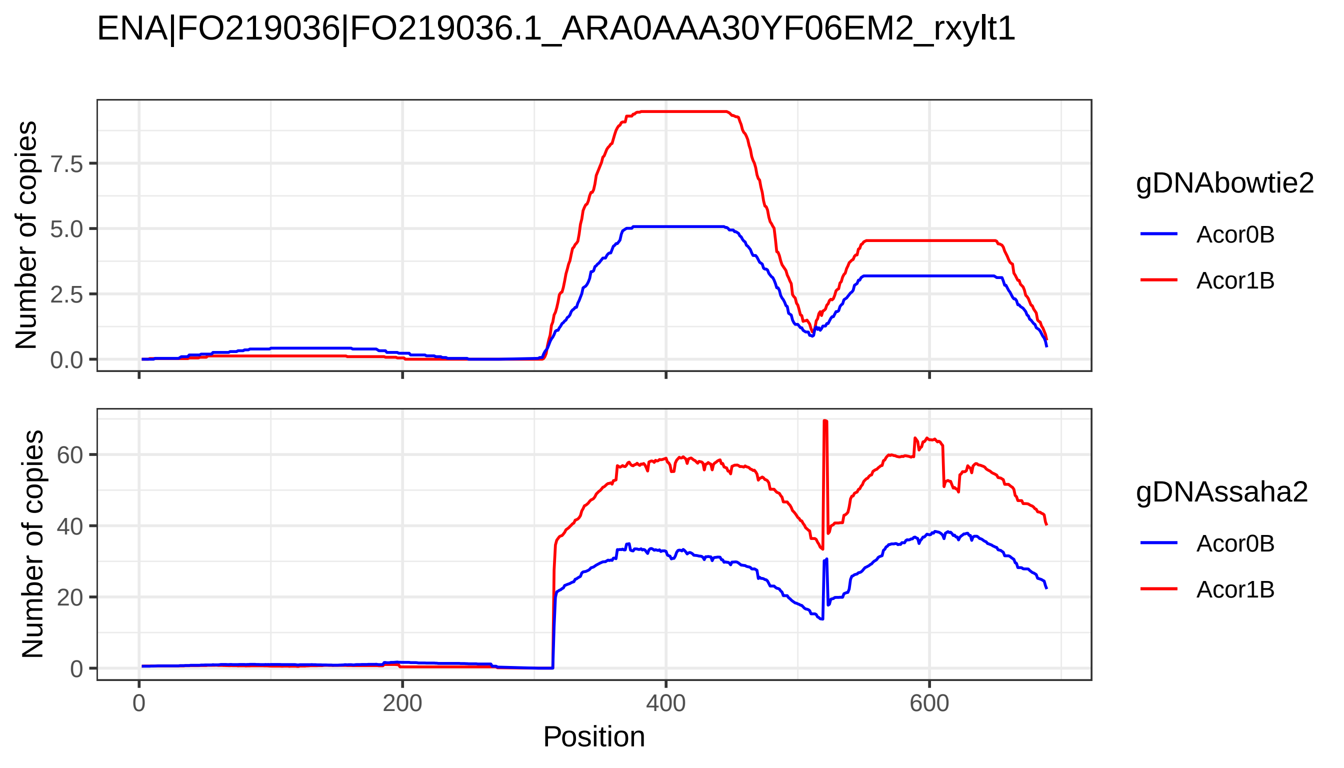
ENA|FO219036|FO219036.1_ARA0AAA30YF06EM2_rxylt1
Number of copies
7.5
5.0
2.5
0.0
gDNAbowtie2
Acor0B
Acor1B
Number of copies
60
40
20
0
gDNAssaha2
Acor0B
Acor1B
0
200
400
600
Position

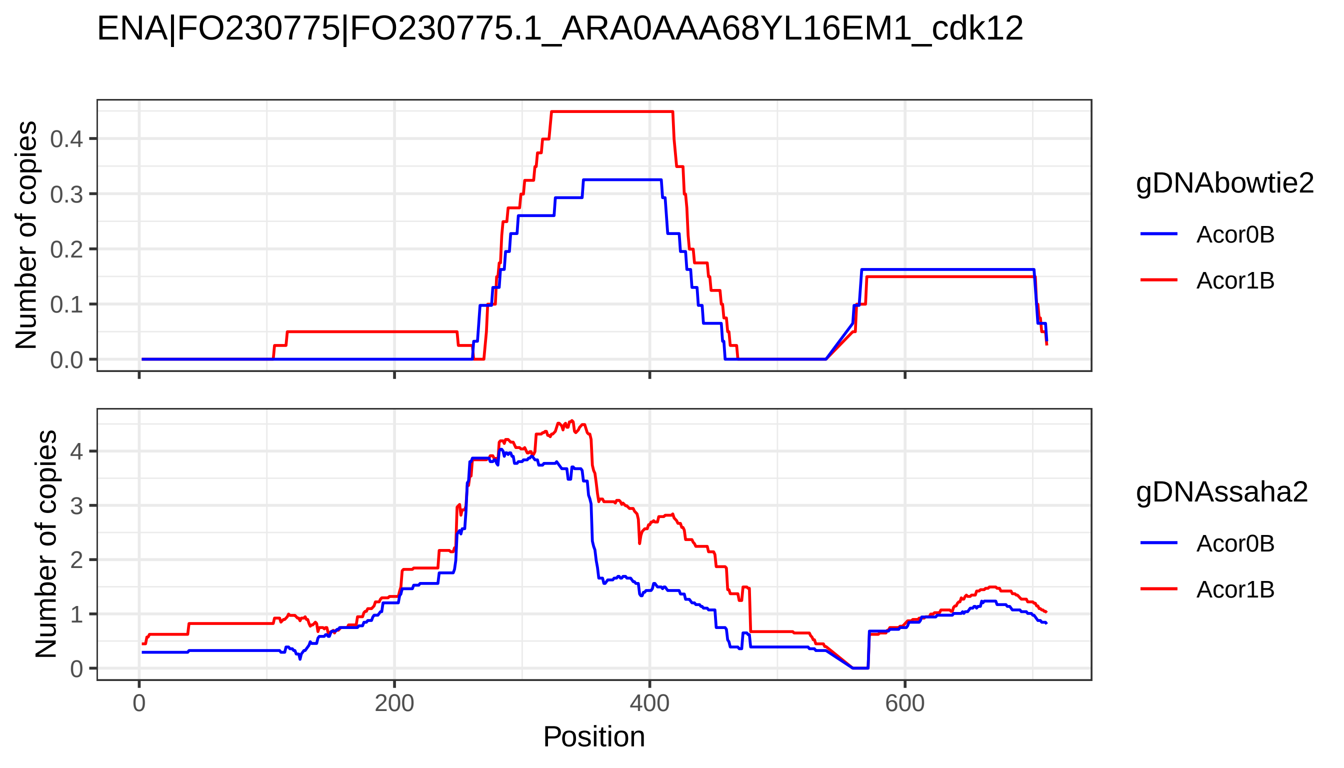
ENA|FO230775|FO230775.1_ARA0AAA68YL16EM1_cdk12
Number of copies
0.0
0.1
0.2
0.3
0.4
gDNAbowtie2
Acor0B
Acor1B
Number of copies
0
1
2
3
4
gDNAssaha2
Acor0B
Acor1B
0
200
400
600
Position

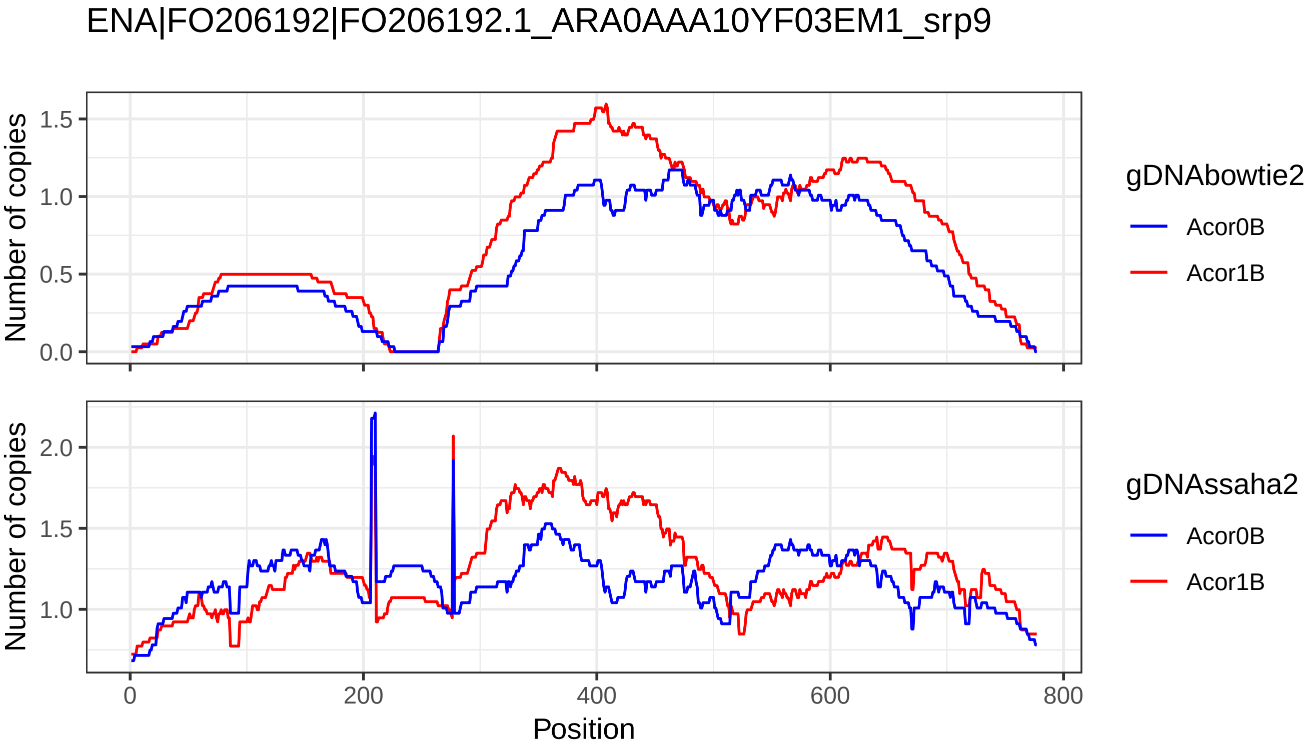

ENA|FO206192|FO206192.1_ARA0AAA10YF03EM1_srp9
Number of copies
1.5
1.0
0.5
0.0
2.0
1.5
1.0
0
200
400
600
800
Position
gDNAbowtie2
Acor0B
Acor1B
gDNAssaha2
Acor0B
Acor1B

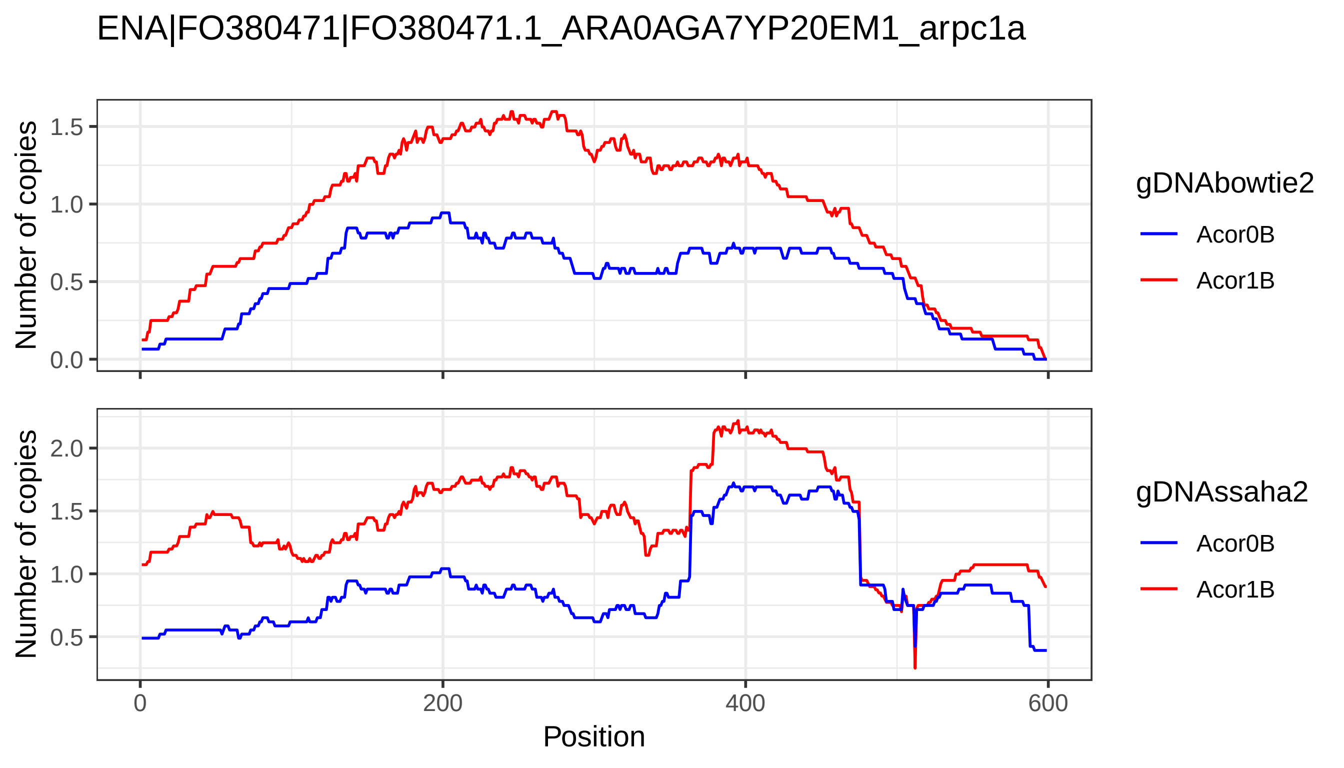
ENA|FO380471|FO380471.1_ARA0AGA7YP20EM1_arpc1a
Number of copies
0.0
0.5
1.0
1.5
gDNAbowtie2
Acor0B
Acor1B
Number of copies
0.5
1.0
1.5
2.0
gDNAssaha2
Acor0B
Acor1B
0
200
400
600
Position

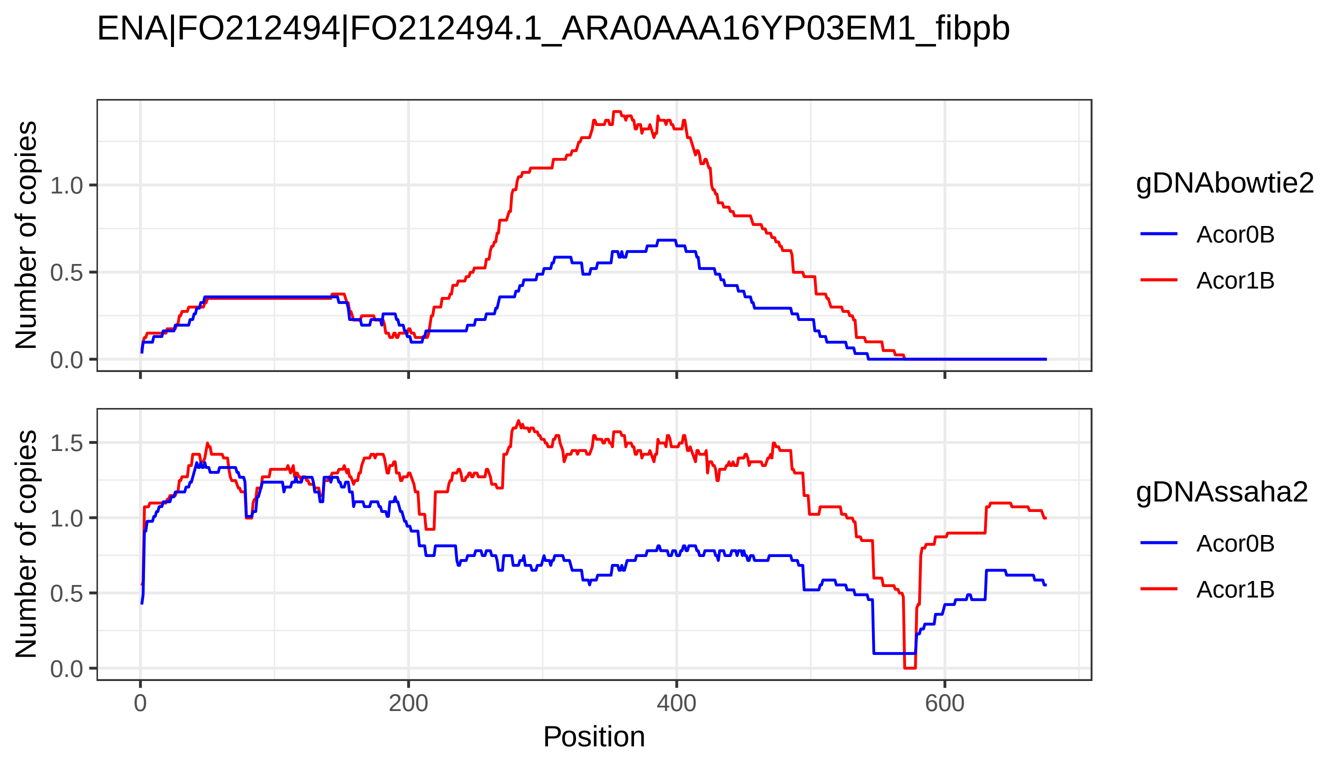

ENA|FO212494|FO212494.1_ARA0AAA16YP03EM1_fibpb
Number of copies
1.0
0.5
0.0
gDNAbowtie2
Acor0B
Acor1B
Number of copies
1.5
1.0
0.5
0.0
gDNAssaha2
Acor0B
Acor1B
0
200
400
600
Position

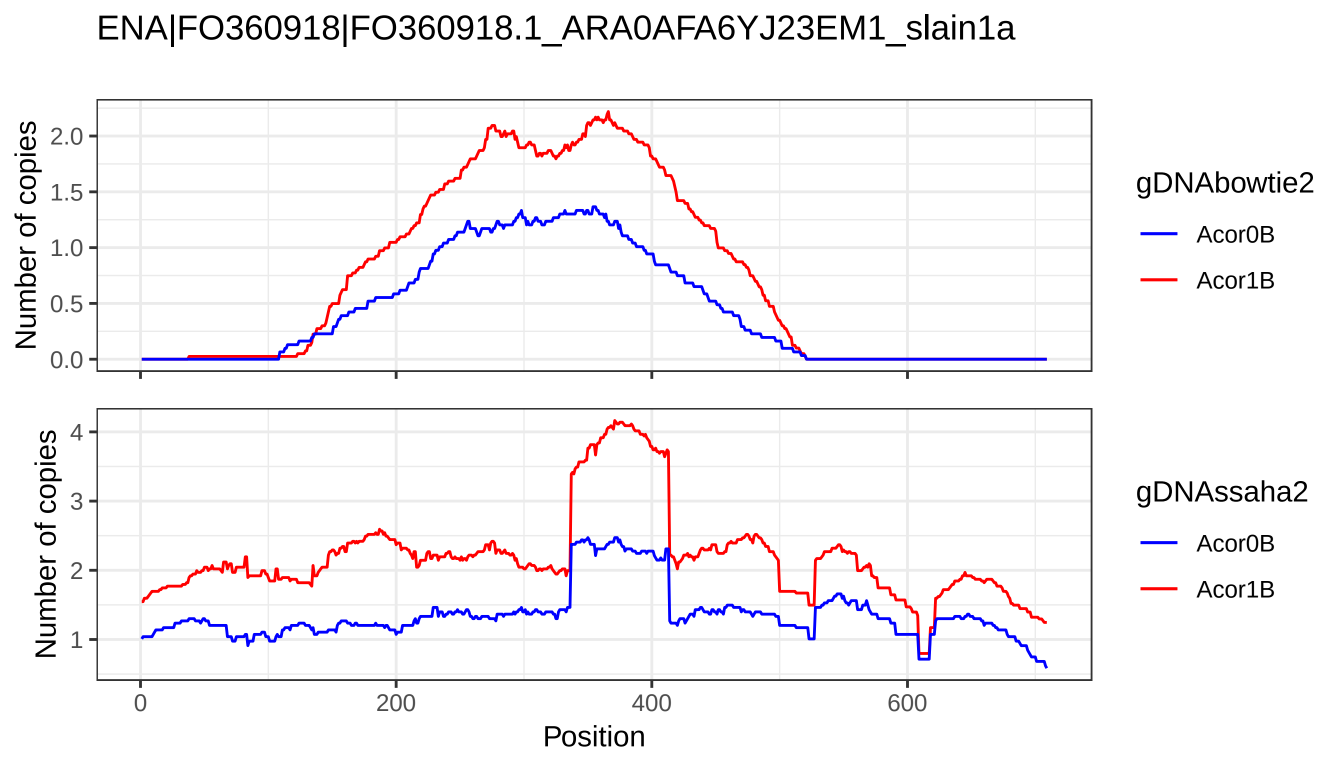
ENA|FO360918|FO360918.1_ARA0AFA6YJ23EM1_slain1a
Number of copies
2.0
1.5
1.0
0.5
0.0
gDNAbowtie2
Acor0B
Acor1B
Number of copies
4
3
2
1
gDNAssaha2
Acor0B
Acor1B
0
200
400
600
Position

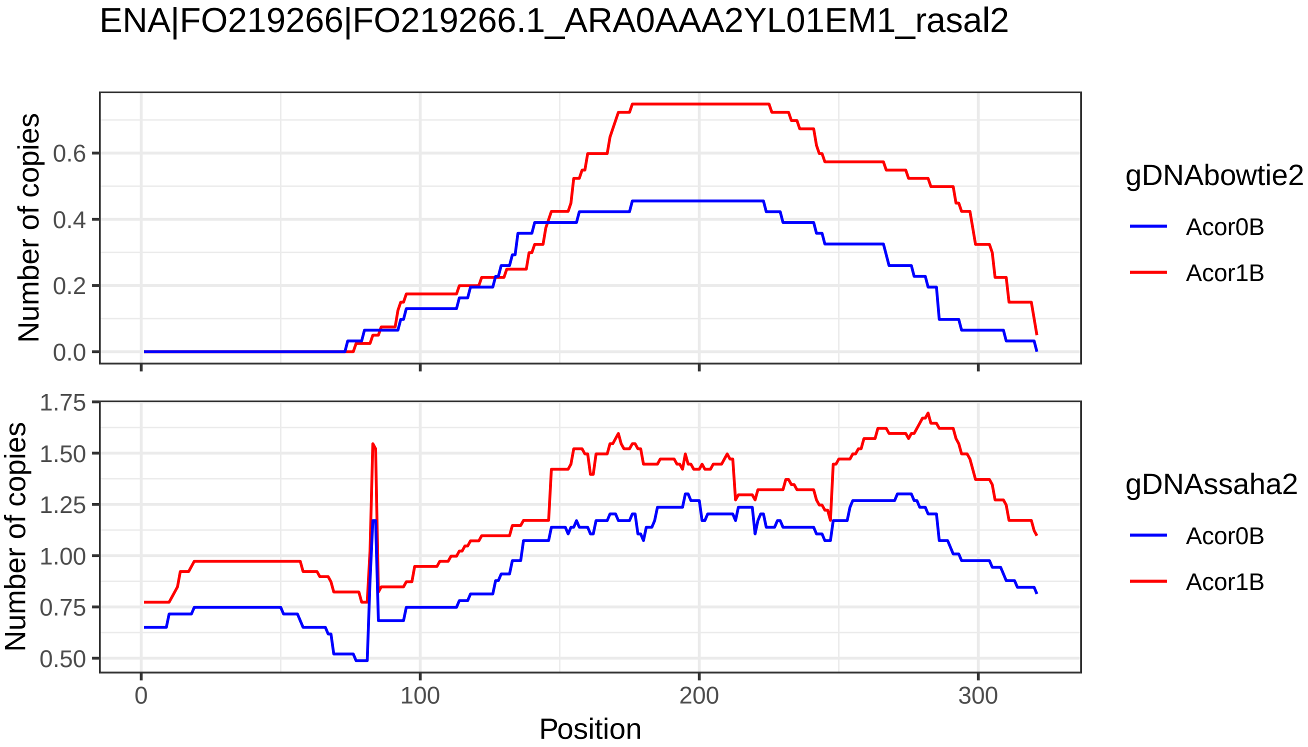
ENA|FO219266|FO219266.1_ARA0AAA2YL01EM1_rasal2
Number of copies
0.0
0.2
0.4
0.6
gDNAbowtie2
Acor0B
Acor1B
Number of copies
0.50
0.75
1.00
1.25
1.50
1.75
gDNAssaha2
Acor0B
Acor1B
0
100
200
300
Position

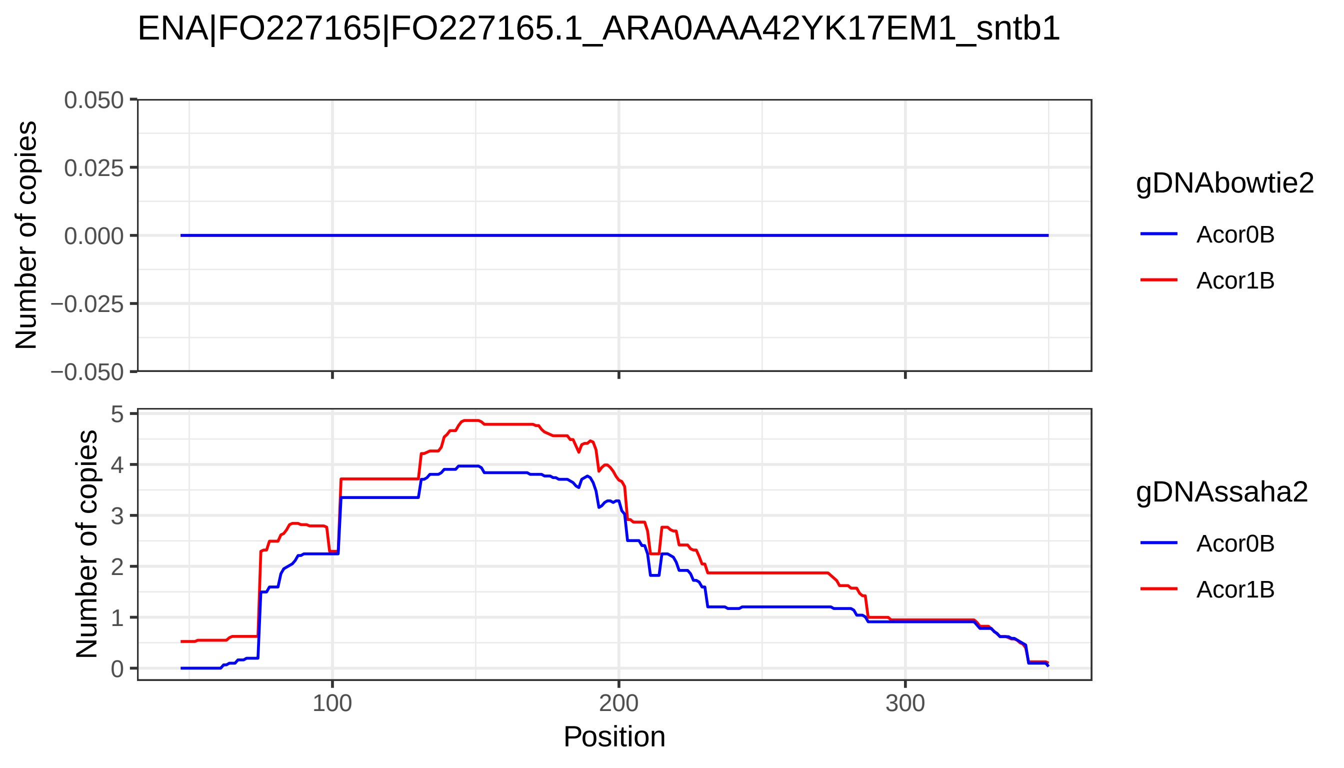
ENA|FO227165|FO227165.1_ARA0AAA42YK17EM1_sntb1
Number of copies
0.050
0.025
0.000
−0.025
−0.050
gDNAbowtie2
Acor0B
Acor1B
Number of copies
5
4
3
2
1
0
100
200
300
Position
gDNAssaha2
Acor0B
Acor1B

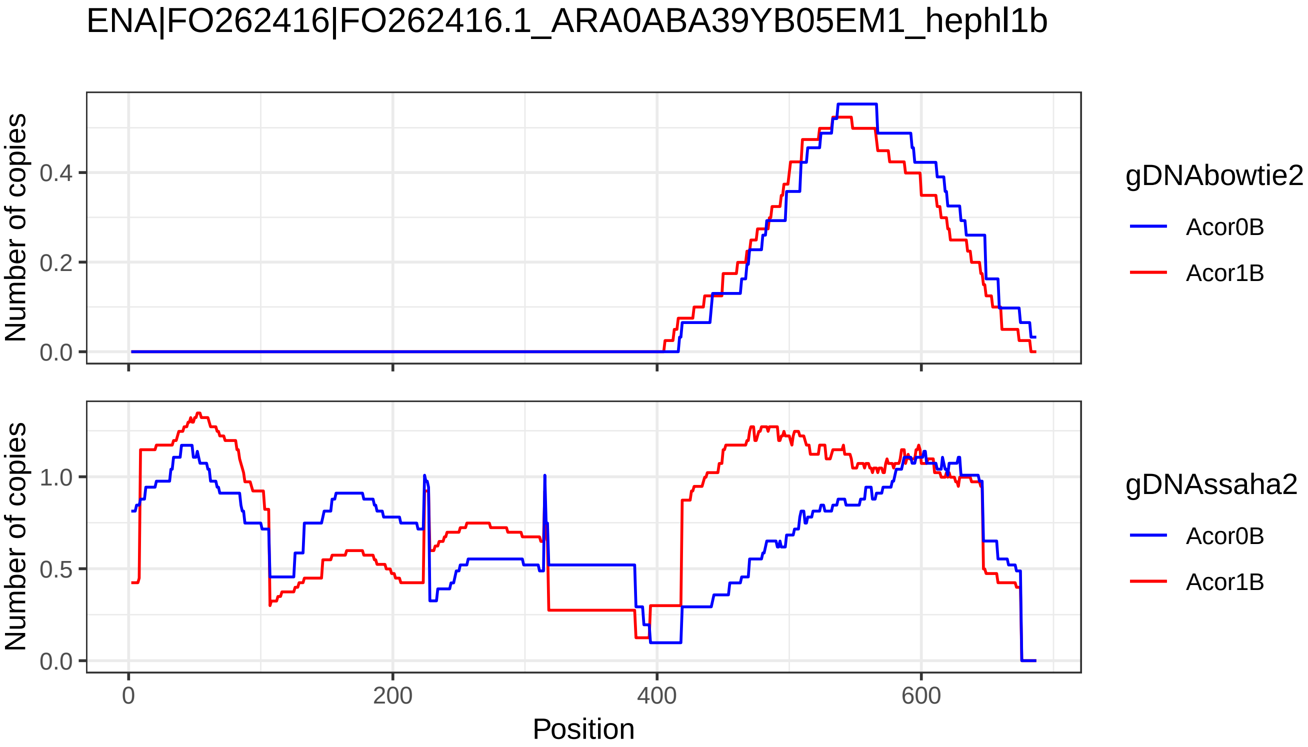
ENA|FO262416|FO262416.1_ARA0ABA39YB05EM1_hephl1b
Number of copies
0.0
0.2
0.4
0.0
0.5
1.0
0
200
400
600
Position
gDNAbowtie2
Acor0B
Acor1B
gDNAssaha2
Acor0B
Acor1B

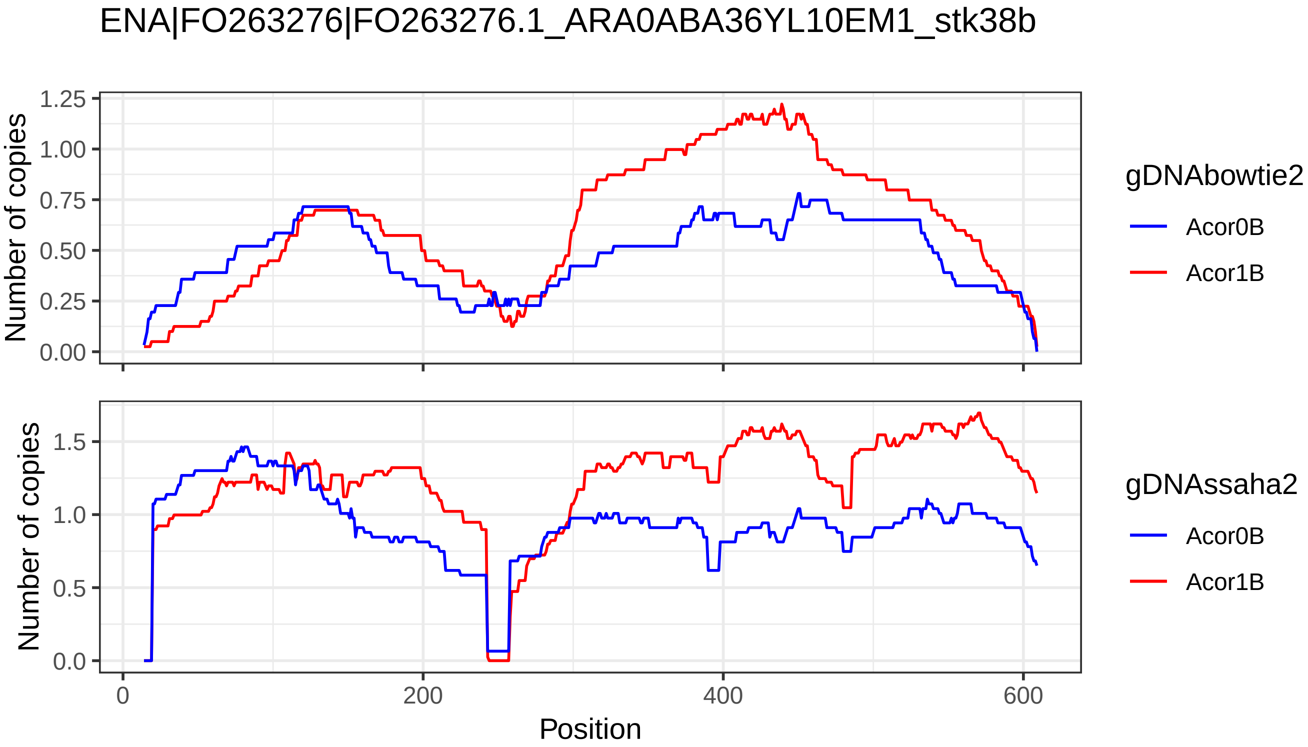

ENA|FO263276|FO263276.1_ARA0ABA36YL10EM1_stk38b
Number of copies
1.25
1.00
0.75
0.50
0.25
0.00
gDNAbowtie2
Acor0B
Acor1B
Number of copies
1.5
1.0
0.5
0.0
gDNAssaha2
Acor0B
Acor1B
0
200
400
600
Position

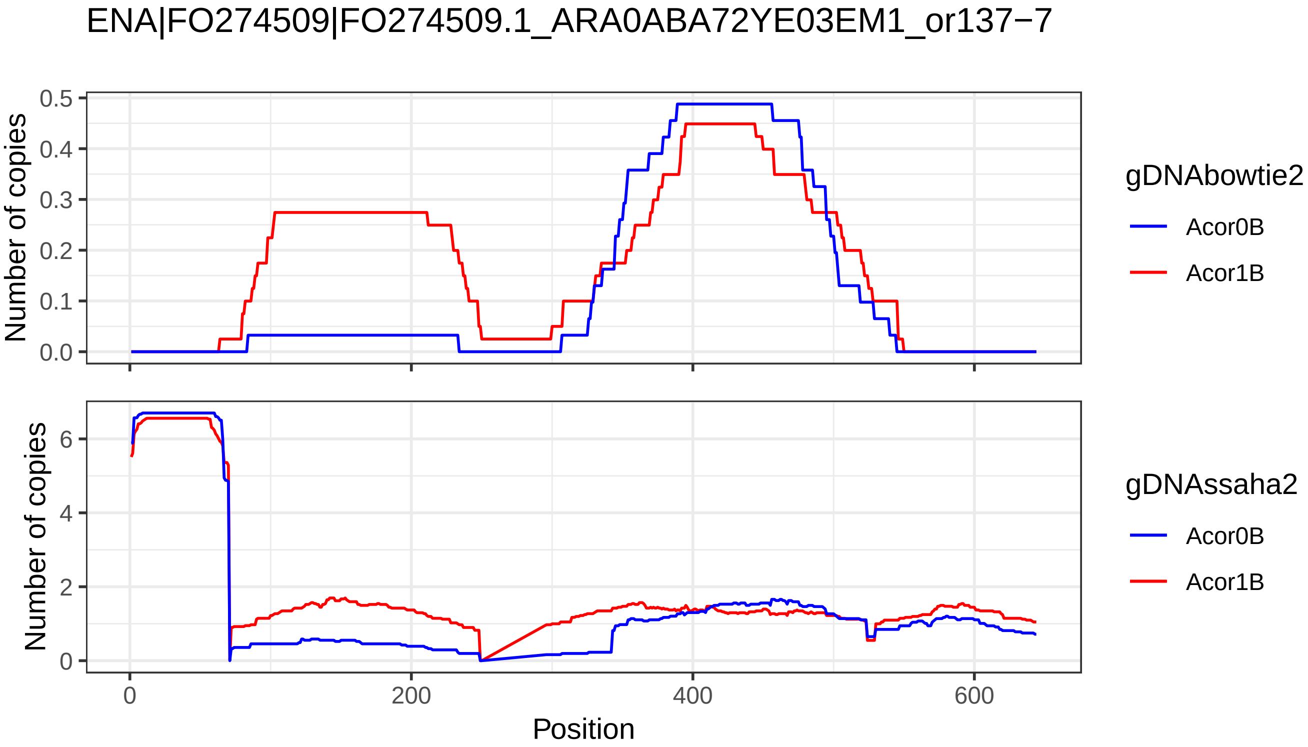
ENA|FO274509|FO274509.1_ARA0ABA72YE03EM1_or137−7
Number of copies
0.0
0.1
0.2
0.3
0.4
0.5
0
2
4
6
0
200
400
600
Position
gDNAbowtie2
Acor0B
Acor1B
gDNAssaha2
Acor0B
Acor1B

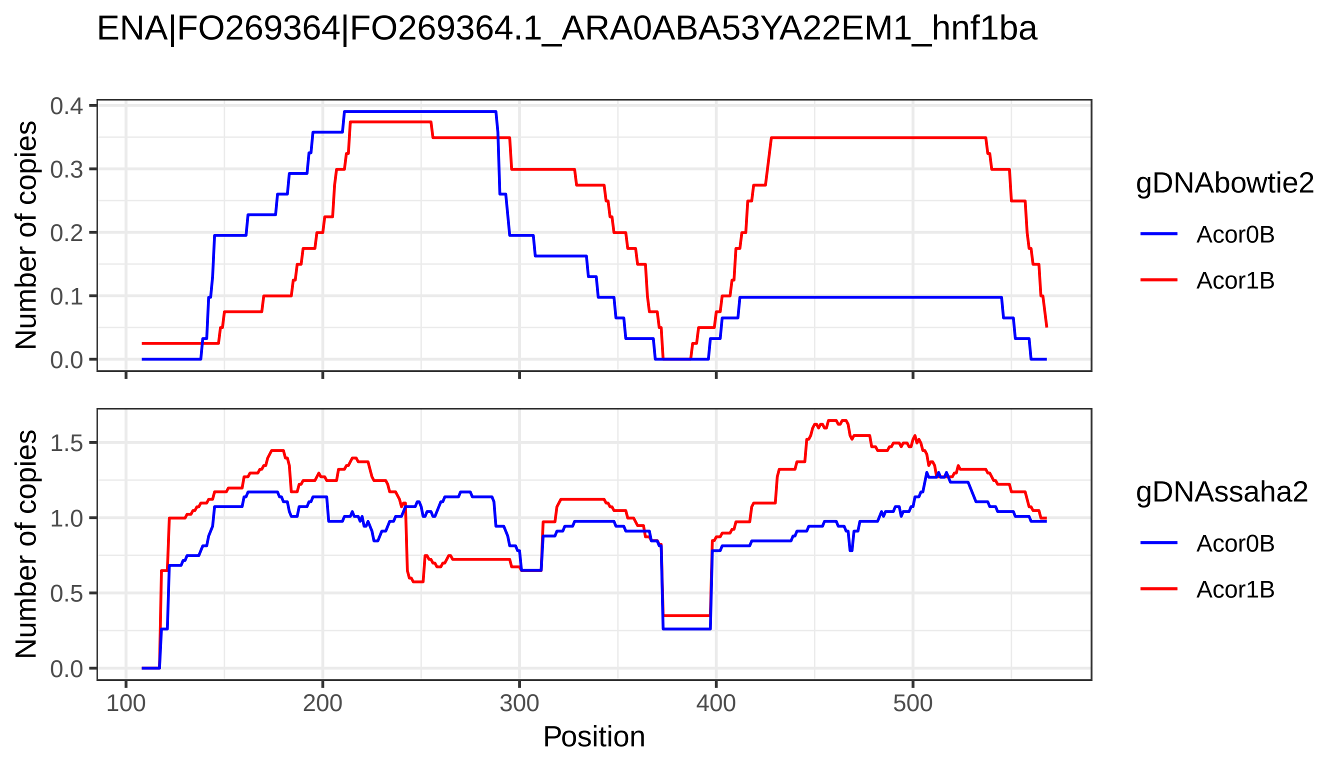
ENA|FO269364|FO269364.1_ARA0ABA53YA22EM1_hnf1ba
Number of copies
0.0
0.1
0.2
0.3
0.4
0.0
0.5
1.0
1.5
100
200
300
400
500
Position
gDNAbowtie2
Acor0B
Acor1B
gDNAssaha2
Acor0B
Acor1B

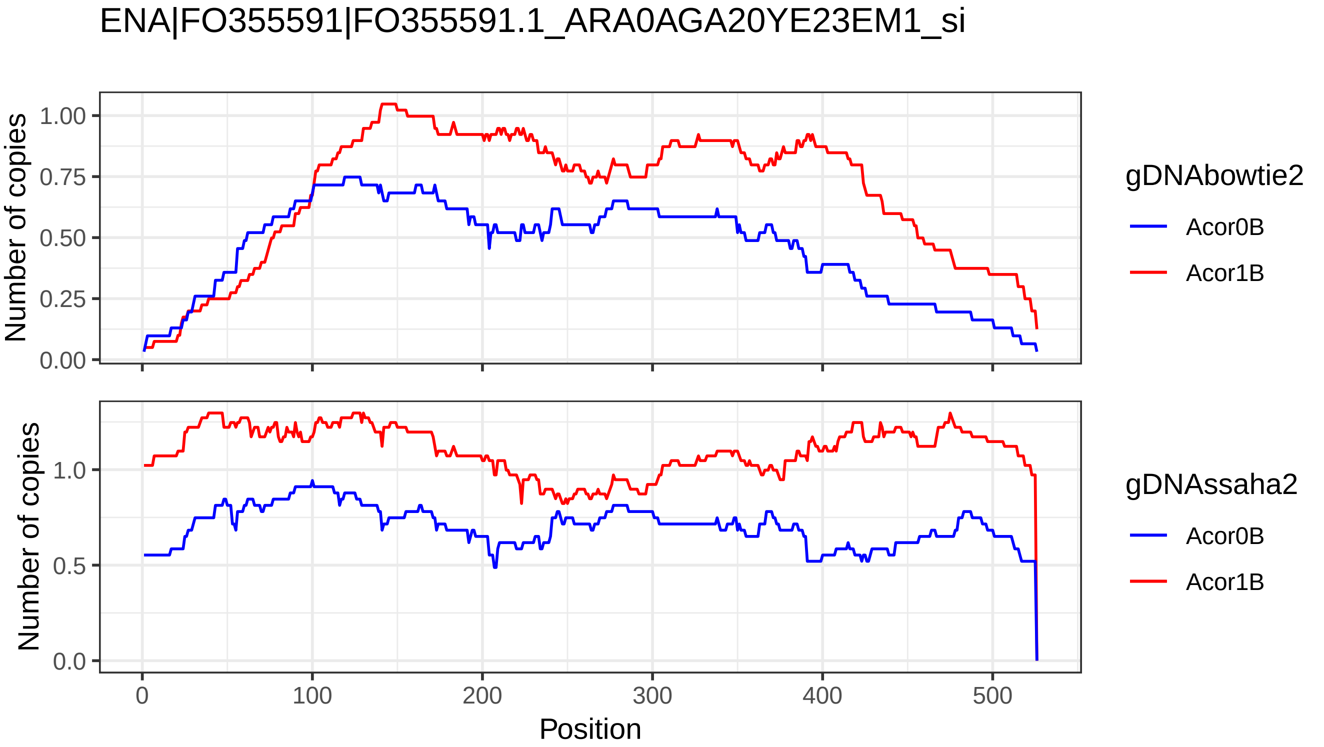
ENA|FO355591|FO355591.1_ARA0AGA20YE23EM1_si
Number of copies
1.00
0.75
0.50
0.25
0.00
gDNAbowtie2
Acor0B
Acor1B
Number of copies
1.0
0.5
0.0
gDNAssaha2
Acor0B
Acor1B
0
100
200
300
400
500
Position

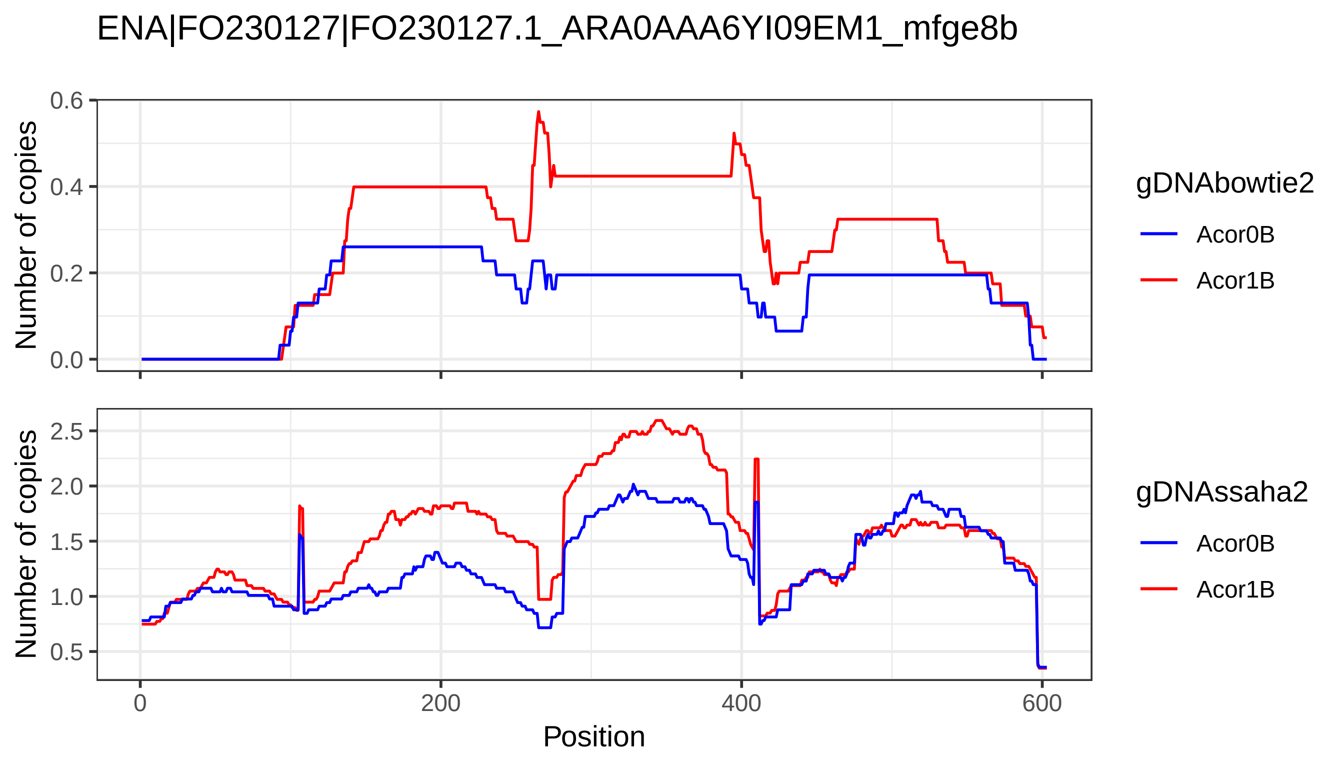
ENA|FO230127|FO230127.1_ARA0AAA6YI09EM1_mfge8b
Number of copies
0.6
0.4
0.2
0.0
gDNAbowtie2
Acor0B
Acor1B
Number of copies
2.5
2.0
1.5
1.0
0.5
gDNAssaha2
Acor0B
Acor1B
0
200
400
600
Position

ENA|FO282399|FO282399.1_ARA0ABA84YI10EM1_emilin3a

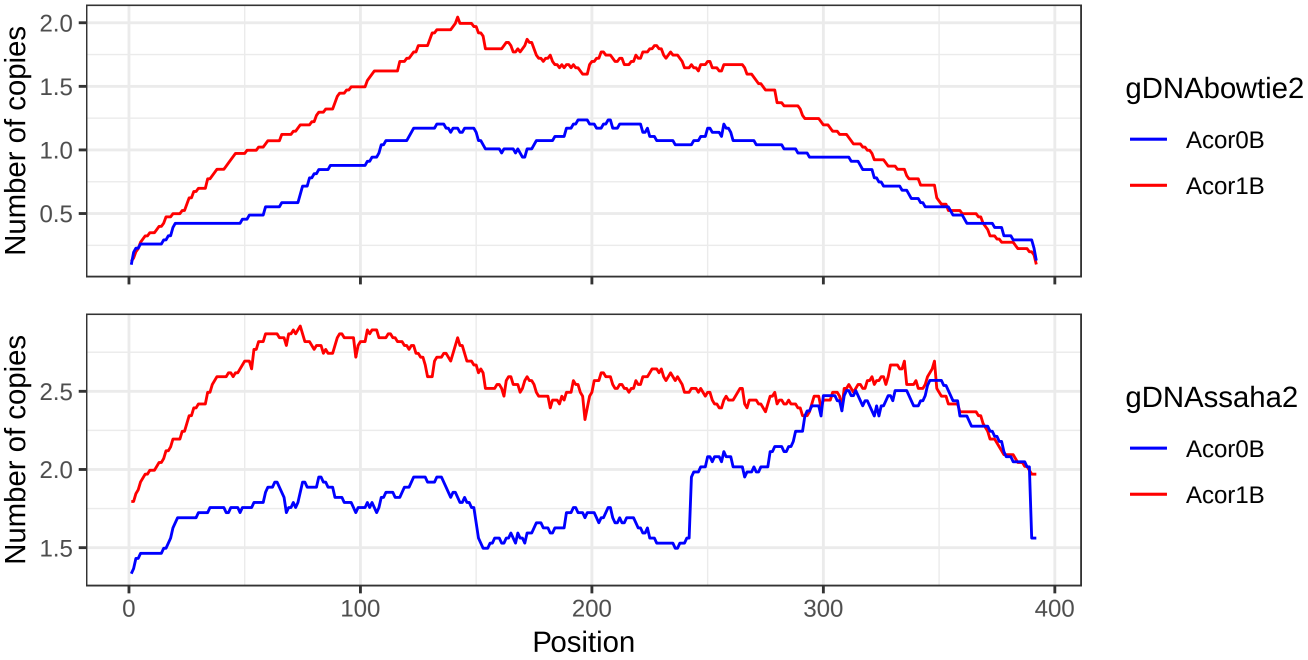

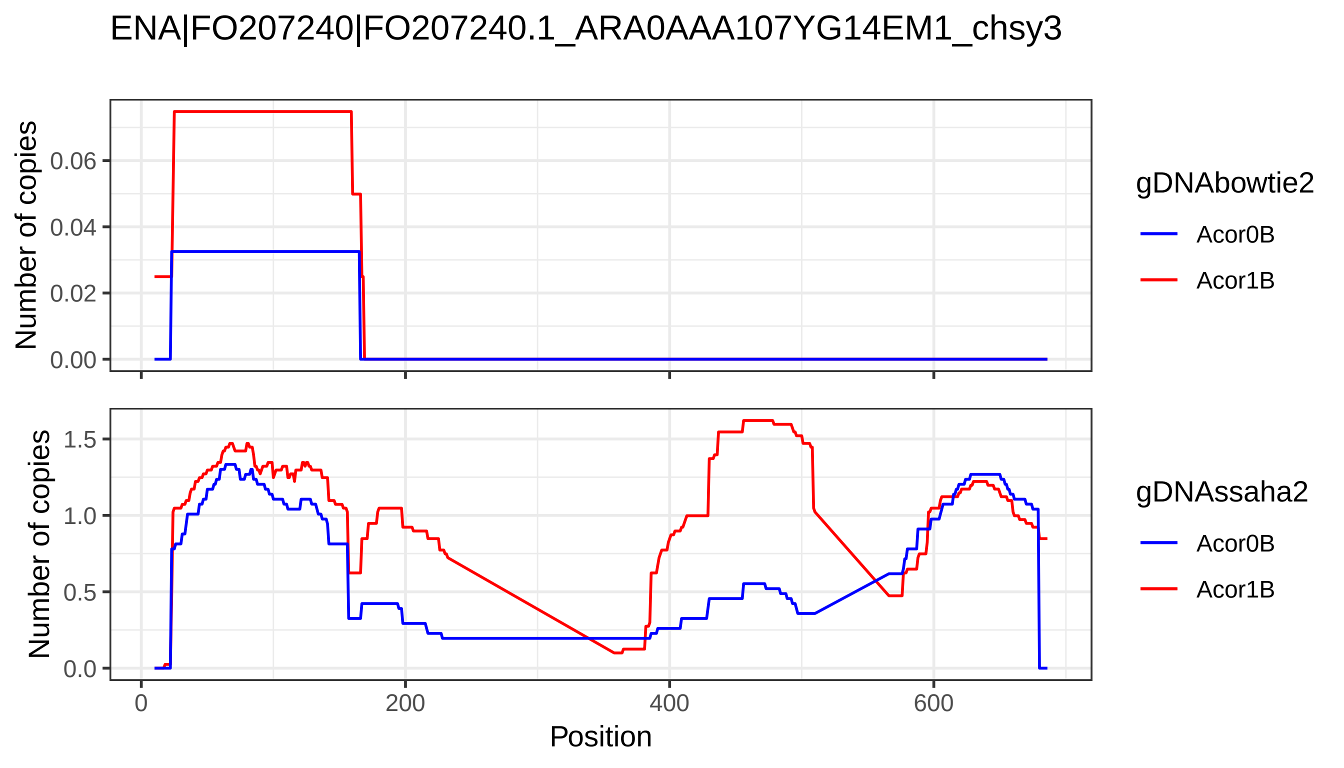

ENA|FO207240|FO207240.1_ARA0AAA107YG14EM1_chsy3
Number of copies
0.00
0.02
0.04
0.06
gDNAbowtie2
Acor0B
Acor1B
Number of copies
0.0
0.5
1.0
1.5
gDNAssaha2
Acor0B
Acor1B
0
200
400
600
Position

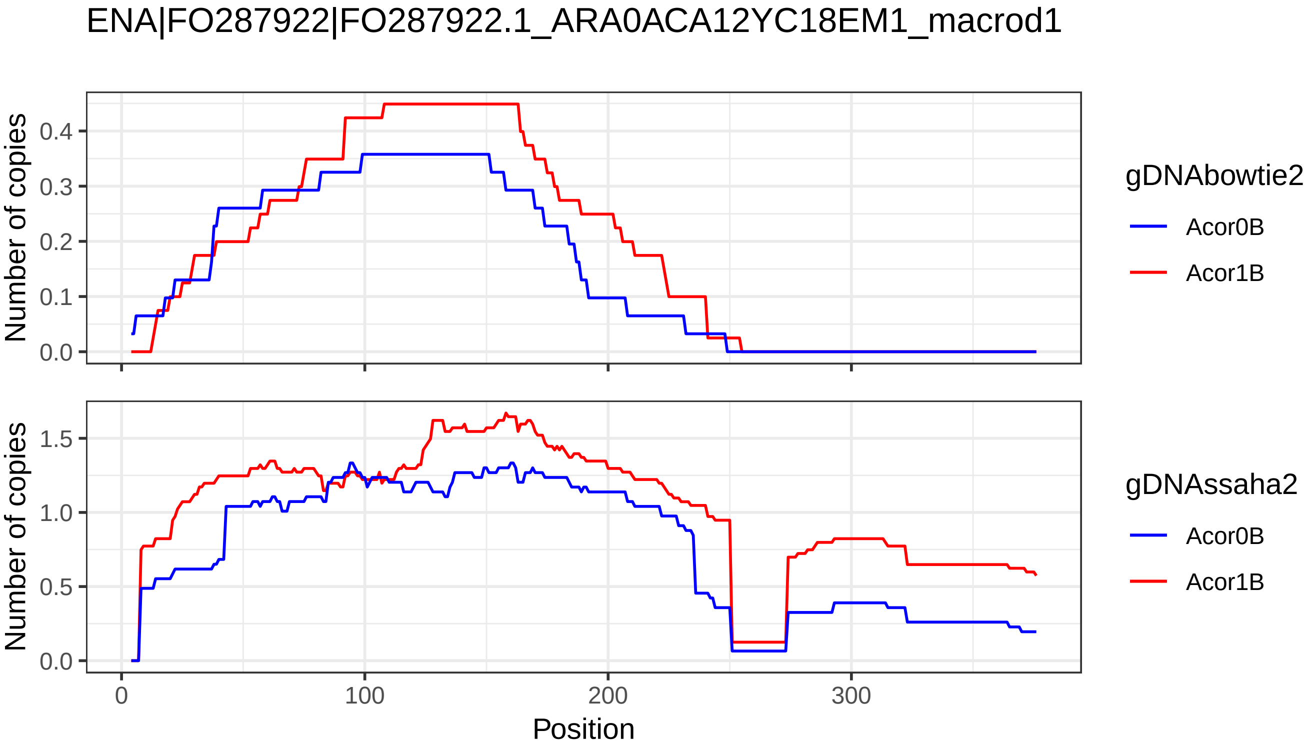

ENA|FO287922|FO287922.1_ARA0ACA12YC18EM1_macrod1
Number of copies
0.0
0.1
0.2
0.3
0.4
gDNAbowtie2
Acor0B
Acor1B
Number of copies
0.0
0.5
1.0
1.5
gDNAssaha2
Acor0B
Acor1B
0
100
200
300
Position

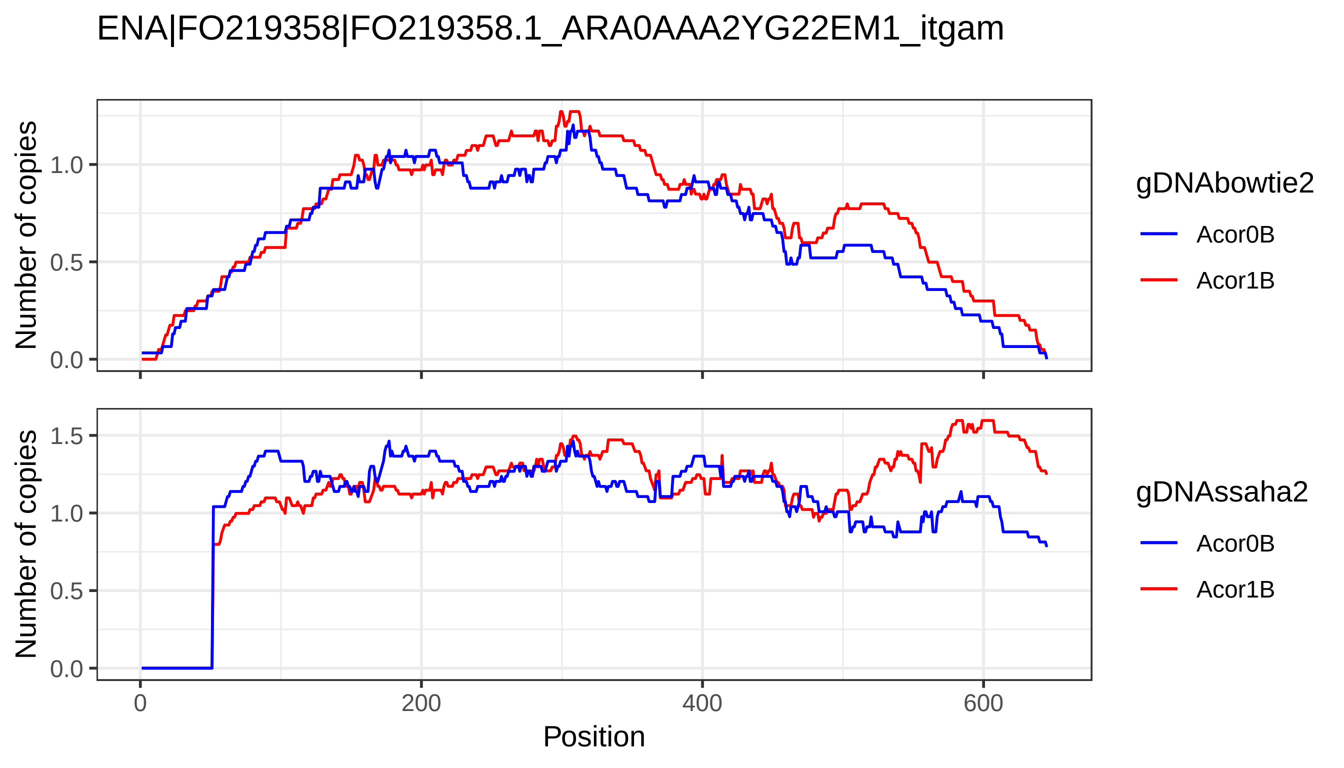
ENA|FO219358|FO219358.1_ARA0AAA2YG22EM1_itgam
Number of copies
0.0
0.5
1.0
1.5
0
200
400
600
Position
gDNAbowtie2
Acor0B
Acor1B
gDNAssaha2
Acor0B
Acor1B

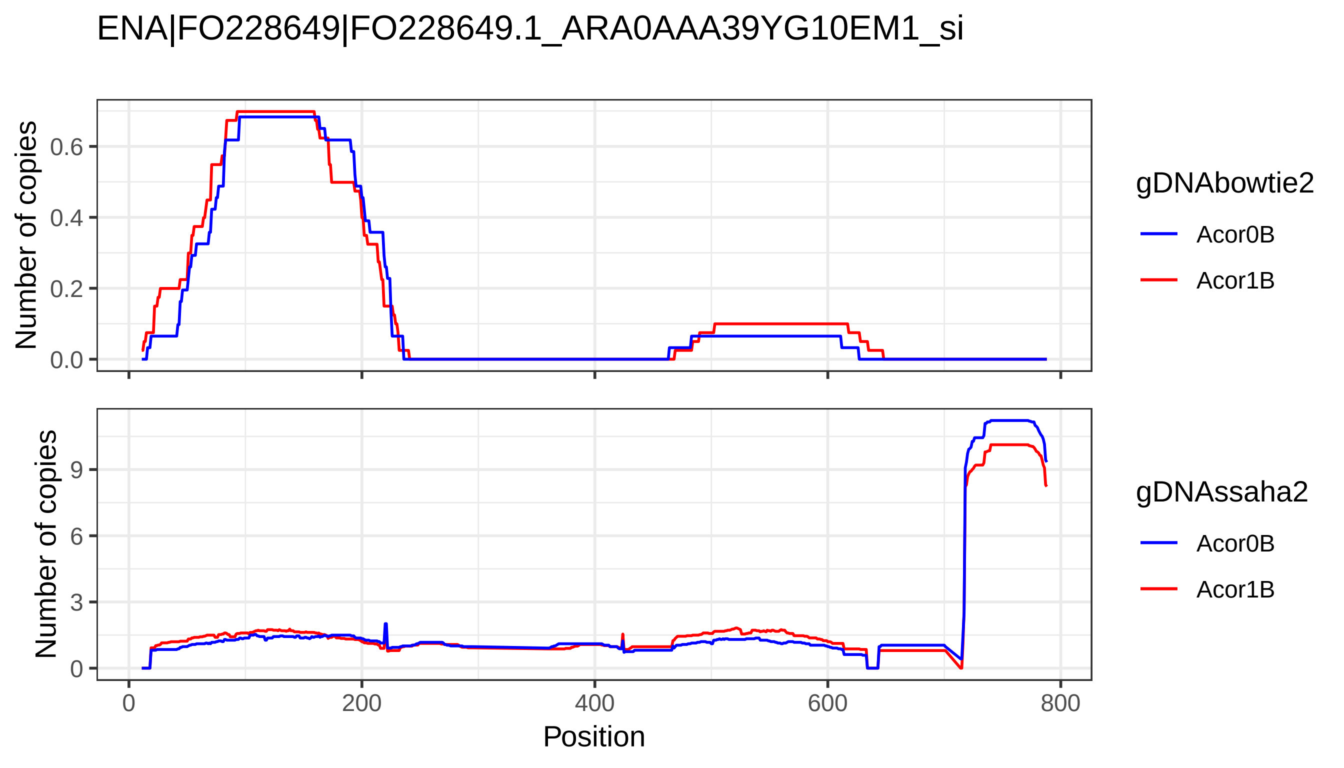

ENA|FO228649|FO228649.1_ARA0AAA39YG10EM1_si
Number of copies
0.0
0.2
0.4
0.6
gDNAbowtie2
Acor0B
Acor1B
Number of copies
0
3
6
9
gDNAssaha2
Acor0B
Acor1B
0
200
400
600
800
Position

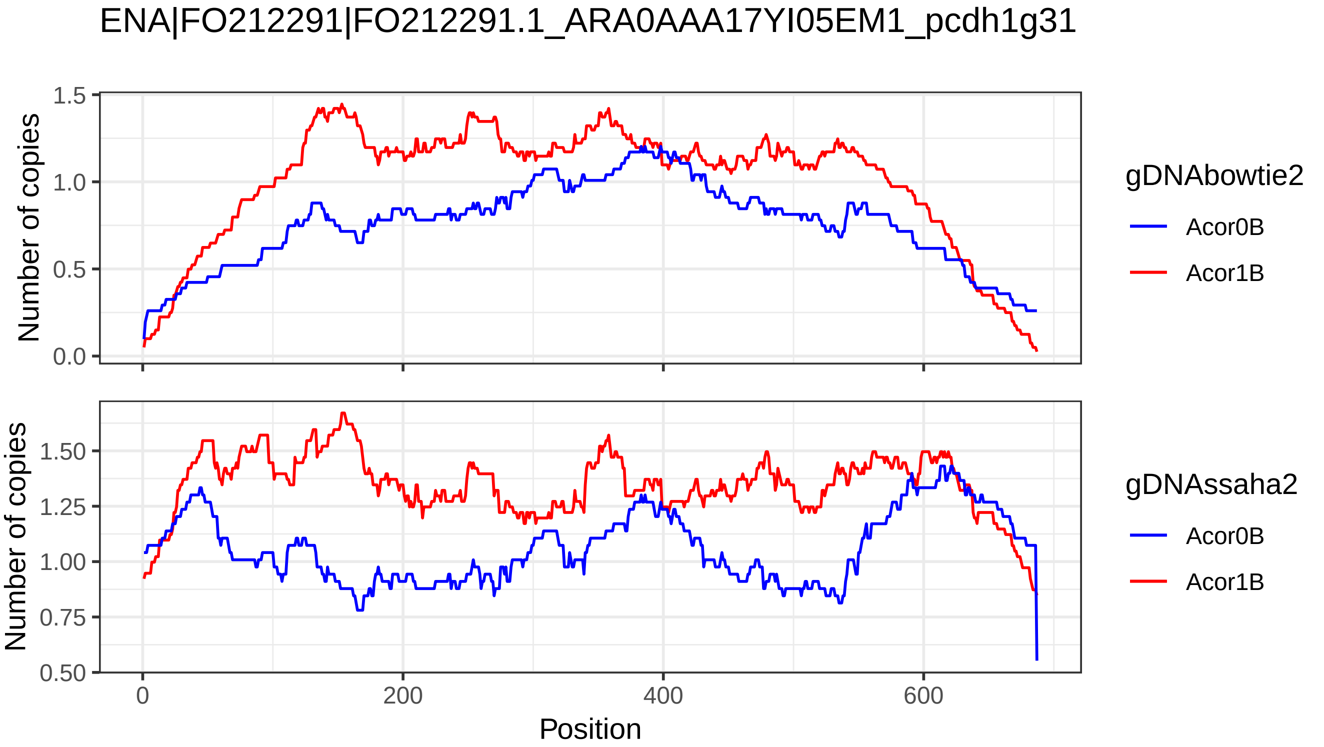
ENA|FO212291|FO212291.1_ARA0AAA17YI05EM1_pcdh1g31
Number of copies
1.5
1.0
0.5
0.0
gDNAbowtie2
Acor0B
Acor1B
Number of copies
1.50
1.25
1.00
0.75
0.50
gDNAssaha2
Acor0B
Acor1B
0
200
400
600
Position
