# Supplementary material for: Irreproducible results and unsupported conclusions in Ahmad et al. [BMC genomics (2020) 21:656]
Source: BMC Genomics. 2023 Dec 18;24:778. doi: 10.1186/s12864-023-09883-4 (PMC10726643; doi:10.1186/s12864-023-09883-4)
Supplement: Supplementary file 9 — Complementary text to better understand our methods, results and discussion. [file 12864_2023_9883_MOESM9_ESM.doc]

**Supplementary text notes (**Complementary text to better understand our methods, results and discussion)

**Supplementary text note 1**

As claimed in [1], “*The CDS with the highest log2 quotient representing a high confidence of B chromosome presence are listed a Table 3 and Supplementary dataset 3 for each species. The coverage pattern for some of these CDS were also visualized to confirm the higher peaks for B+ as compared to B- genomes, hence providing evidence of their expanded copy number on B chromosomes (Supplementary Figs. S7, S8, S9)*”. We tried to verify this information for *A. mexicanus* and A. *correntinus* (Figs S7 and S8, respectively), but we realized that some of the accession numbers in the plot were absent in Sup_Dataset_3. For brevity in the following explanation, we associated the accessions numbers given in figures S7 and S8 with species name and sequence number, as shown in Table S1.

| Table S1 Incongruence between the information contained in Figures S7 and S8 and Supplementary Dataset 3 in [1]. Only some of the accession numbers mentioned in Figures S7 and S8 in [1] are found in its Supplementary Dataset 3 file, whereas those in bold-type letter are missing. | | |
| --- | --- | --- |
| Figure | Our name | Accession number |
| Fig. S7: *A. mexicanus* | Amex1 | ARA0AAA39YG16EM1 |
|  | Amex2 | **ARA0ABA18YP03EM1** |
|  | Amex3 | **ARA0AAA100YB14EM1** |
|  | Amex4 | **ARA0ABA86YJ16EM1** |
|  | Amex5 | **ARA0AAA109YO19EM1** |
|  | Amex6 | **ARA0ABA110YF05EM1** |
|  | Amex7 | **ARA0AAA114YD18EM2** |
|  | Amex8 | ARA0AGA13YG24EM1 |
| Fig. S8: *A. correntinus* | Acor1 | **ARA0AAA7YM13EM1** |
|  | Acor2 | ARA0AAA105YI15EM1 |
|  | Acor3 | **ARA0AAAYO20EM1** |
|  | Acor4 | ARA0ABA89YC04EM1 |
|  | Acor5 | ARA0AAA27YN14EM1 |
|  | Acor6 | ARA0ABA89YC04EM1 |
|  | Acor7 | **ARA0AAA69YE12EM1** |
|  | Acor8 | ARA0ABA32YK17EM1 |

In the case of *A. mexicanus*, only Amex1 and Amex8 were found in Sup_Dataset_3, whereas the six remaining accessions numbers were missing. Likewise, for *A. correntinus*, we only found five out of the eight accession numbers whereas Acor1, Acor3 and Acor7 were missing in Sup_Dataset_3. In addition, Acor2 was the only sequence in Fig. S8 annotated as protein-coding gene (*tars*) for *A. correntinus* in Sup_Dataset_3, the four remaining (that were present in this dataset) were "unknown". It was however confusing that the coverage profile displayed about 1000 nt for this contig whereas the contig we got from EBI had only 629 nt.

**Supplementary text note 2**

In methods, it reads: "*The transcriptome assemblies of A. mexicanus (used as reference for A. mexicanus and A. correntinus) and Locust migratoria (used as reference for A. flavolineata) were retrieved from NCBI database (accession IDs: GDIO00000000.1, PRJNA237016)*". However the IDs are mistaken, as GDIO00000000.1 contains data from *L. migratoria* whereas PRJNA237016 includes data from *A. mexicanus*. In addition, the sequence IDs are not in NCBI, as indicated. For instance, Sup_dataset_3 says that the transcript ID for the *acaa*1 gene is "ARA0ABA101YH11EM1.b.am.1". However, even deleting the last part (".b.am.1"), no result is found in NCBI: <https://www.ncbi.nlm.nih.gov/search/all/?term=ARA0ABA101YH11EM1>

However, we realized that these "transcript ids" (so called in Sup_dataset_3) are actually in the EBI repository, where a similar search (for “ARA0ABA101YH11EM1”) found a sequence with the name "*Astyanax mexicanus* surface fish mRNA 5-prime sequence from clone ARA0ABA101YH11 (ARA0ABA101YH11EM1)": <https://www.ebi.ac.uk/ebisearch/search.ebi?query=ARA0ABA101YH11EM1&db=allebi&submit1=Search&requestFrom=ebi_index>, although no annotation was associated with this name in the database. For this reason, we downloaded from EBI the sequences of the 61 contigs annotated as protein-coding genes in Sup_dataset_3 (*A. correntinus* sheet) (see these sequences in our Supplementary_file_6) and searched for these nucleotide sequences in the NR protein database from NCBI by means of BLASTX, restricted to "bony fishes (taxid:7898)". Considering the description of the best BLASTX coincidences and comparing them with the gene symbol shown in Sup_dataset_3, using GeneCards ([www.genecards.org](http://www.genecards.orgi/)) to search for synonym terms, we found only 16 contigs with the same annotation specified in [1] (Sup_dataset_3), whereas 18 contigs gave a discrepant annotation and the 27 remainder were not found in the BLASTX search (see full data in our Supplementary_file_7).

In addition, we analyzed the sequences of the *A. mexicanus* assembled transcriptome included in the previously mentioned NCBI bioproject (PRJNA237016) (<https://www.ncbi.nlm.nih.gov/bioproject/PRJNA237016/>) and downloaded the 46,987 "transcripts" (<https://www.ncbi.nlm.nih.gov/nuccore?linkname=bioproject_nuccore_transcript&from_uid=237016>). On the basis of the gene names assigned to the 61 contigs annotated as known genes for *A. correntinus*, in Sup_dataset_3, we could identify only 26 contigs in the *A. mexicanus* transcriptome from NCBI (see sequences in our Supplementary_file_8), and BLASTX showed that they yielded the expected homology with *A. mexicanus* genes.

**Supplementary text note 3**

When we downloaded authors' *A. correntinus* libraries from SRA, which corresponded to the access numbers mentioned in the paper: SRR11147339 (+B) and SRR11147340 (0B), they contained 505,608,854 and 387,817,038 reads, respectively. These figures coincided with the "Number of filtered reads” in Table 1, suggesting that these libraries had already been trimmed. In addition, we found that read length varied within libraries. All this indicates the absence of raw reads in SRA.

To test this possibility, we tried to reproduce the trimming process explained in the paper. However, this was rather difficult as authors claimed to have performed two trimmings, one with Trimmomatic and another with the FASTx toolkit. In fact, the second step could have been obviated by modifying some Trimmomatic parameters. Therefore, methods in [1] read: *"... low quality reads were discarded, and adapters were trimmed using the Trimmomatic tool [69]. Specific filtering parameters were set in the commands according to requirements for removing adapters and poor quality reads as per the FastQC tool [70] report. Filtering of reads was performed by FASTx toolkit using parameters set to quality number 28 and percentage value 80 for alignments"*. Note that citations within sentences in italic letter, e.g. [69], are references in [1].

The parameters employed in [1] are in the following repository:

<https://github.com/farhan-phd/Integrative-genomic-analysis-reveals-the-gene-contents-repeats-landscapes-and-evolutionary-dynamics/blob/master/Steps-protocol-commands-scripts-B-chromome.txt> (accessed: 6 May 2021), but the order in which they perform these two trimmings in the script is the opposite to that claimed in the text, so it is impossible to know the actual order performed. As inferred from the following commands in the script:

$ fastq_quality_filter -q 28 -p 80 -i input-raw.fq -o ouput-filtered.fq -Q33

$ pairfq makepairs -f R1_001_filtered.fastq -r R2_001_filtered.fastq -fp R1_001.paired.fq -rp R2_001.paired.fq -fs R1_001.single.fq -rs R2_001.single.fq

$ java -jar trimmomatic-0.30.jar PE R1_001_filtered.fastq R2_001_filtered.fastq trimmed_forward_paired.fq trimmed_forward_unpaired.fq trimmed_reverse_paired.fq trimmed_reverse_unpaired.fq ILLUMINACLIP:TruSeq3-PE.fa:2:30:10 LEADING:3 TRAILING:3 SLIDINGWINDOW:4:15 MINLEN:36

they first used “fastq_quality_filter” from the FASTx toolkit, then selected the paired reads with “pairfq” (a step not included in paper's methods) and then used Trimmomatic.

Due to the difficulty to understand the two trimming steps made by authors and the doubt about whether they submitted to SRA already trimmed data, we opted for doing a conventional trimming of the reads with Trimmomatic, using the same parameters indicated by authors', hoping that all reads submitted by authors would meet the trimming criteria. However, some reads were filtered out and thus the number of reads remaining (497,573,030 and 381,021,840 in the 1B and 0B libraries, respectively) was lower than the corresponding figures in Table 1 of [1]. For this reason, to try to reproduce the paper results, we performed our mappings in the next section with two sets of reads: i) this trimmed collection, and ii) the reads downloaded from SRA without additional filtering. The results were essentially similar and we show here our mappings with the libraries as they are in SRA.

**Supplementary text note 4**

Table 3 in [1] shows the "*Results of mapping gDNA reads from 0B and 1(2) B of A. mexicanus, A. correntinus and A. flavolineata on transcriptome CDS*", and the corresponding full data is given in Sup_dataset_3. We found no correspondence between Table 3 and Sup_dataset_3 for the information about *A. mexicanus* and *A. correntinus*, since they are mistakenly interchanged, so that, if Sup_dataset_3 is correct, the *A. mexicanus* mappings in Table 3 actually correspond to *A. correntinus*, and *vice versa*, as can easily be verified by cross-searching transcript IDs. In addition, Sup_dataset_3 is not free from errors, such as, for instance, in the "2B reads" and "0B/1B" headings of the *A. correntinus* sheet, as they should read "1B reads" and "1B/0B", respectively.

**Supplementary text note 5**

We first performed the mappings using the same software used in [1] (Bowtie2),

with the same options indicated in <https://github.com/farhan-phd/Integrative-genomic-analysis-reveals-the-gene-contents-repeats-landscapes-and-evolutionary-dynamics/blob/master/Steps-protocol-commands-scripts-B-chromome.txt> (accessed: 6 May 2021):
$ bowtie2 --very-sensitive -x reference-genome-indexed.fa -1 0B_mysample_forward_paired.fq -2 0B_mysample_reverse_paired.fq -U 2B-unpaired_01.fastq,2B-unpaired_02.fastq, -p 64 -S 0B_verysens.sam

Note that since the *A. correntinus* library in SRA is completely paired, we did not use the -U option to include unpaired reads. See mapping results in Supplementary_file_1.

**Supplementary text note 6**

For comparison, we also performed mappings with the SSAHA2 version 2.5.5 software, with the options we usually apply to map genomic reads on a transcriptome reference, considering clipped reads which are avoided by the --very-sensitive option in Bowtie2 version 2.3.3.1 used in [1]. The advantage of clipping is that it allows for partial mapping, which is crucial for reads partly containing introns which are not represented in the reference sequences. We ran SSAHA2 using our script deposited in <https://github.com/fjruizruano/ngs-protocols/blob/master/ssaha2_run_multi.py>.

We expressed coverage mapping in two ways: "number of reads" (as in [1]) and "copy number" (as in [2]). In the first case, the figures were given either without normalizing for library size (as in [1]), using our own script (<https://github.com/fjruizruano/ngs-protocols/blob/master/count_reads_bam.py>), or normalizing them for library size in order to test the effect of this missing action in [1] calculations. In the second case, copy numbers were normalized per library and genome sizes, following [2].

**Supplementary text note 7**

During our reading of [1], we noticed other errors and unsupported claiming:

1) The first sentence in the Background section (page 2) says: *"B chromosomes (Bs) are additional and non-essential extra chromosomes, which show non-Mendelian inheritance and lack the ability of meiotic pairing unlike the normal A chromosomes [1, 2]."* In our opinion, it is wrong to say that B chromosomes "lack the ability of meiotic pairing". B-bivalents have commonly been reported in many B chromosome systems, e.g., in *Locusta migratoria* (see an example in [9]). It is however true that B chromosomes do not pair with A chromosomes, that the number of B chromosomes is not fixed to two per genome (as A chromosomes), that they do not always form bivalents, and that this impedes their regular segregation. Nevertheless, B chromosomes actually have the ability of meiotic pairing as also shown by the formation of synaptonemal complexes, including for auto-pairing iso-B-chromosomes [10, 11].

2) Some references are not appropriately used. For instance, on the right column of page 2, first paragraph, it says: *"However, the knowledge obtained about the molecular composition of B chromosomes in this group of species [referring to grasshoppers], focus on the characterization of B repetitive genomic content [24–26]"*. However, reference 26 corresponds to our group’s paper analyzing the satellitome of the grasshopper *Pyrgomorpha* *conica* [12], a species lacking B chromosomes. In the same vein, most of the literature cited after the sentence *"Several studies have previously found that Bs are generally enriched with TEs [4, 24, 33, 53–59]"*, on page 16, does not actually deal with TE analyses on B chromosomes. For instance, two of them are authored by our research group, but one is about histone genes on B chromosomes in *Locusta migratoria* [13]*,* and the other about rDNA and a satellite DNA on the B chromosomes of the grasshopper *Eyprepocnemis plorans* [14].

3) The null hypothesis enounced on page 2 is invalid: *"Here, we sequenced and analyzed the B carrier genomes of the insect A. flavolineata and the fishes A. correntinus and A. mexicanus to reveal their B-linked repetitive and gene content, to test the hypothesis that the B chromosome accumulates sequences from its host genome for its selfish transmission."* In our opinion, the fact that selfish transmission has not been shown in any of the three species analyzed in this paper invalidates this null hypothesis.

4) Claiming for conclusions unsupported by the available evidence is evident on page 2, where it reads *"We found evidences that considerable amount of genomic portions have been migrated from A chromosomes to B via transpositions, duplications and rearrangements events"*. However, findings in [1] only showed that B chromosomes are enriched in transposons (which is not new) and that some B blocks may show indels or other rearrangements (which is expected for dispensable genomic material plenty of repetitive DNA), but we see no evidence supporting that some genomic portions other than TEs have migrated from A to B chromosomes. In addition, they should have also mentioned that Martis et al. [15] already suggested that B chromosomes in rye contain A-derived DNA sequences and acknowledge that their model in Figure 9 is partly based on this idea.

5) On page 3, it reads: *"It seems that B chromosomes tend to gain sequences that are crucial for their own establishment inside the cell"*. This is an anti-Darwinian post-adaptive statement leading to the unsupported conclusion that *"Besides the genes that may give transmission advantage to Bs, there are others coding for many important biological processes"*. However, the mere presence of genes on B chromosomes does not grant that they are crucial for them or the host genome. It is necessary to identify B-derived transcripts and show that they are translated to polypeptides that alter some metabolic reactions increasing B transmission, which is still far from being shown in any species (see [16]). Of course, it can only be tested in species where B chromosome drive and fitness effects have been analyzed, which is not the case for the three species analyzed in [1]. In this respect, it is overselling to proclaim -on page 4, first paragraph- that the three study species are "model species". That could be true for *A. mexicanus*, but it is highly doubtful for the two other species.

6) The paper includes inappropriate data from microdissected B chromosomes and reaches strong but unsupported conclusions on B chromosome gene content: On page 2 (right column), it reads *"the genetic composition of isolated Bs in different species was facilitated by flow-sorting and micro-dissection techniques [34]. However, these techniques provide limited material and thus do not fully reveal the relationship of homologous sequences between A and B chromosomes and the complete gene content of Bs".* This is contradictory with using the results of Illumina sequencing of microdissected B chromosomes in several organisms to reach some of the main conclusions of the paper (see below). Also, at the end of page 16, it reads that *"These commonly enriched functions shared among the Bs of different species suggest that B chromosomes exhibit a conserved behavior to acquire a certain role, although their genetic makeup in may vary across different taxa"*. Although it is tough to extract some meaning from this cryptic sentence, the claiming for “a conserved behavior to acquire a certain role” is teleological and unsupported by all current knowledge about B chromosomes.

In addition, we find it inappropriate the use of Illumina reads obtained from microdissected B chromosomes, with extremely low coverage and clear bias towards repetitive sequences, to infer GO functions without specifying the actual number of genes which they are based on. At the end of page 13, it says: *"The gene annotation recorded for all microdissected Bs revealed several genes overlapping with their reference annotations. Due to the low coverage of sequencing data, the number of genes in microdissected B sequences can be underrepresented. Interestingly, the GO enrichment analysis of the Bs in different organisms shared some common over represented functions such as metabolism, development and morphogenesis (Fig. 8b, c, d; Supplementary Table S4; Supplementary dataset 7)"*. If genes are underrepresented in the Illumina sequences obtained from microdissected B chromosomes, we believe that the subsequent GO analysis performed by authors is not reliable. In any case, the names of the genes from which these GO terms were inferred should have been given in any of the tables or datasets, as its omission impedes readers to assess how many genes actually yielded the 21 GO terms shown in dataset_7 for *Eyprepocnemis plorans*, 25 for *Apodemus flavicolis*, 3 for *A. peninsuale*, and 6 for *Lates calcarifer*. It is thus difficult to know whether the conclusion, at the end of page 17 and beginning of page 18, that *"this paper offers contributions about the genomic composition, evolutionary and functional aspects of multiple B chromosomes in different species”* is supported by a sufficient number of genes.

7) The suggestion (in the beginning of page 17) that *"Bs might have played some role in shaping the genome evolution for effective adaptation in cave environment”* in the case of *A. mexicanus* is not supported by this paper results, and more when the list of B-genes for this species reported in [1] coincides only in one gene (*ncaph2*) with the list of B-genes recently reported by Imarazene et al. [17]. Even though both analyses would have dealed with different B chromosomes, and bearing also in mind our comments on point no. 6, we consider that Ahmad et al. hypothesis (in [1]) that *"B chromosomes plays a role in adaptation acting on metabolisms"* is untenable.

8) Finally, we find highly inappropriate converting the title of our 2019 bioRxiv preprint [5] literally into an “emerging hypothesis” without mentioning the source. On page 16, column on the right, it reads: *"In addition to the fragmented genes, there are complete genes that have remained intact, possibly due to their role in the evolutionary survival of the B chromosome. These findings support the emerging hypothesis reporting B-localized genes [36, 37] according to which B chromosomes accumulate cell cycle genes that might play an important role in their transmission"*. This emerging hypothesis was first suggested as a conjecture in *Astatotilapia latifasciata* [18], even though no evidence for B drive had been shown in this species. Three years later, we provided evidence for this hypothesis in the grasshopper *Eyprepocnemis plorans*, a species where B chromosomes show drive during oogenesis [19] and carry several cell cycle genes which are transcribed [4]. In this last paper, we specifically suggested that the secret for B chromosome success may lie on gene content. In line with our suggestion, Makunin et al., after analyzing gene content of B chromosomes in several mammal species (where B drive is unknown), claimed that "the fixation of Bs within a population may in fact depend on their own genes" [20], without any mention to [4]. In addition, on June 27, 2019 (i.e., one month before the Fourth B chromosome Conference organized by the corresponding author of [1]), we posted in bioRxiv a preprint entitled "**Evolutionary success of a parasitic B chromosome rests on gene content**" where we demonstrated that the *apc1* gene paralog located on the B chromosome in *Locusta migratoria* is transcribed more intensely than the copies on the A chromosomes of the same B-carrying ovaries and, in this species, B chromosomes are also able to drive during oogenesis [21]. It is thus surprising that, without mentioning our bioRxiv preprint, a few lines below authors claim that *"Remarkably, the GO enrichment analyses of different microdissected Bs in different species revealed similar patterns of functions, thus providing evidence to corroborate the emerging hypothesis that the* ***evolutionary success of the B chromosome lies on its gene contents.****"* Here, authors (almost literally) write the title of our 2019 bioRxiv preprint without including it in the reference list.

**References**

[1] Ahmad SF, Jehangir M, Cardoso AL, Wolf IR, Margarido VP, Cabral-de-Mello DC et al. B chromosomes of multiple species have intense evolutionary dynamics and accumulated genes related to important biological processes. BMC Genomics. 2020; 21: 656. <https://doi.org/10.1186/s12864-020-07072-1>

[2] Silva DMZA, Ruiz-Ruano FJ, Utsunomia R, Martín-Peciña M, Castro JP, Freire PP et al. Long-term persistence of supernumerary B chromosomes in multiple species of *Astyanax* fish. BMC Biology. 2021;19:52. <https://doi.org/10.1186/s12915-021-00991-9>

[3] Piscor D, Pozzobon APB, Fernandes CA, Centofante L, Parise-Maltempi PP. Molecular clock as insight to estimate the evolutionary history and times of divergence for 10 nominal *Astyanax* species (Characiformes, Characidae): An evolutionary approach in species with 2n = 36, 46, 48, and 50 chromosomes. Zebrafish. 2019; 16: 98-105. <https://doi.org/10.1089/zeb.2018.1647>

[4] Navarro-Domínguez B, Ruiz-Ruano FJ, Cabrero J, Corral JM, López-León MD, Sharbel TFet al. Protein-coding genes in B chromosomes of the grasshopper *Eyprepocnemis plorans*. Sci. Rep. 2017; 7: 45200. <https://doi.org/10.1038/srep45200>

[5] Ruiz-Ruano FJ, Navarro-Domínguez B, López-León MD, Cabrero J, Camacho JPM. Evolutionary success of a parasitic B chromosome rests on gene content. bioRxiv. 2019; <https://doi.org/10.1101/683417>

[6] Guindon S, Dufayard JF, Lefort V, Anisimova M, Hordijk W, Gascuel O. New algorithms and methods to estimate maximum-likelihood phylogenies: assessing the performance of PhyML 3.0. Systematic biology. 2010;59:307-21.

[7] Terán GE, Benitez MF, Mirande JM. Opening the Trojan horse: phylogeny of *Astyanax*, two new genera and resurrection of *Psalidodon* (Teleostei:Characidae). Zool. J. Linn. Soc. 2020; XX: 1–18. <https://doi.org/10.1093/zoolinnean/zlaa019>

[8] Song H, Am C, Cigliano MM, Desutter L, Heads SW, Huang Y et al. 300 million years of diversification: elucidating the patterns of orthopteran evolution based on comprehensive taxon and gene sampling. Cladistics. 2015; 31: 621–51. <https://doi.org/https://doi.org/10.1111/cla.12116>

[9] Cabrero J, Viseras E, Camacho JPM The B-chromosomes of *Locusta migratoria* I. Detection of negative correlation between mean chiasma frequency and the rate of accumulation of the B’s; a reanalysis of the available data about the transmission of these B-chromosomes. Genetica. 1984; 64: 155–164. <https://doi.org/10.1007/BF00115339>

[10] Mestriner C, Galetti P, Valentini S, Ruiz I., Abel L, Moreira-Filho O et al. Structural and functional evidence that a B chromosome in the characid fish *Astyanax scabripinnis* is an isochromosome. Heredity, 85: 1–9. <http://www.ncbi.nlm.nih.gov/pubmed/10971685>

[11] Kichigin IG, Lisachov AP, Giovannotti M, Makunin AI, Kabilov MR, O’Brien PCM et al. First report on B chromosome content in a reptilian species: the case of *Anolis carolinensis*. Mol. Genet. Genomics. 2019; 294: 13–21. <https://doi.org/10.1007/s00438-018-1483-9>

[12] Ruiz-Ruano FJ, Castillo-Martínez J, Cabrero J, Gómez R, Camacho JPM, López-León MD High-throughput analysis of satellite DNA in the grasshopper *Pyrgomorpha conica* reveals abundance of homologous and heterologous higher-order repeats. Chromosoma. 2018; 127: 323–40. <https://doi.org/10.1007/s00412-018-0666-9>

[13] Teruel M, Cabrero J, Perfectti F, Camacho JPM. B chromosome ancestry revealed by histone genes in the migratory locust. Chromosoma. 2010; 119: 217–225. <https://doi.org/10.1007/s00412-009-0251-3>

[14] Cabrero J, Bakkali M, Bugrov A, Warchalowska-Sliwa E, López-León MD, Perfectti F et al. Multiregional origin of B chromosomes in the grasshopper *Eyprepocnemis plorans*. Chromosoma. 2003;112:207–11. https://doi.org/10.1007/s00412-003-0264-2

[15] Martis MM, Klemme S, Banaei-Moghaddam AM, Blattner FR, Macas J, Schmutzer T et al. Selfish supernumerary chromosome reveals its origin as a mosaic of host genome and organellar sequences. Proc. Nat. Acad. Sci. USA. 2012; 109: 13343–46. <https://doi.org/10.1073/pnas.1204237109>

[16] Benetta ED, Akbari OS, Ferree PM Sequence Expression of Supernumerary B Chromosomes: Function or Fluff? Genes. 2019; 10: 123. <https://doi.org/10.3390/GENES10020123>

[17] Imarazene B, Du K, Beille S, Jouano E, Feron R, Pan Q. A supernumerary “B-Sex” chromosome drives male sex determination in the pachón cavefish, *Astyanax mexicanus*. Current Biology (in press).

[18] Valente GT, Conte MA, Fantinatti BEA, Cabral-De-Mello DC, Carvalho RF, Vicari MR et al. Origin and evolution of B chromosomes in the cichlid fish *Astatotilapia latifasciata* based on integrated genomic analyses. Mol. Biol. Evol. 2014; 31: 2061–2072. <https://doi.org/10.1093/molbev/msu148>

[19] Zurita S, Cabrero J, López-León M, Camacho JPM. Polymorphism regeneration for a neutralized selfish B chromosome. Evolution. 1998; 52: 274–77.

<https://doi.org/10.1111/j.1558-5646.1998.tb05163.x>

[20] Makunin, A. I., Romanenko, S. A., Beklemisheva, V. R., Perelman, P. L., Druzhkova, A. S., Petrova, K. O et al. Sequencing of supernumerary chromosomes of red fox and raccoon dog confirms a non-random gene acquisition by B chromosomes. Genes. 2018; 9, 1–14. https://doi.org/10.3390/genes9080405

[21] Pardo MC, López-León MD, Cabrero J, Camacho JPM. Transmission analysis of mitotically unstable B chromosomes *in Locusta migratoria*. Genome. 1994; 37: 1027–34. <https://doi.org/10.1139/g94-146>
